# Supplementary material for: Functional vagotopy in the cervical vagus nerve of the domestic pig: implications for the study of vagus nerve stimulation
Source: J Neural Eng. Author manuscript; Available in PMC 2020 Jun 21. (PMC7306215; doi:10.1088/1741-2552/ab7ad4)
Supplement: supplementary information 1 [file NIHMS1594881-supplement-supplementary_information_1.pdf]

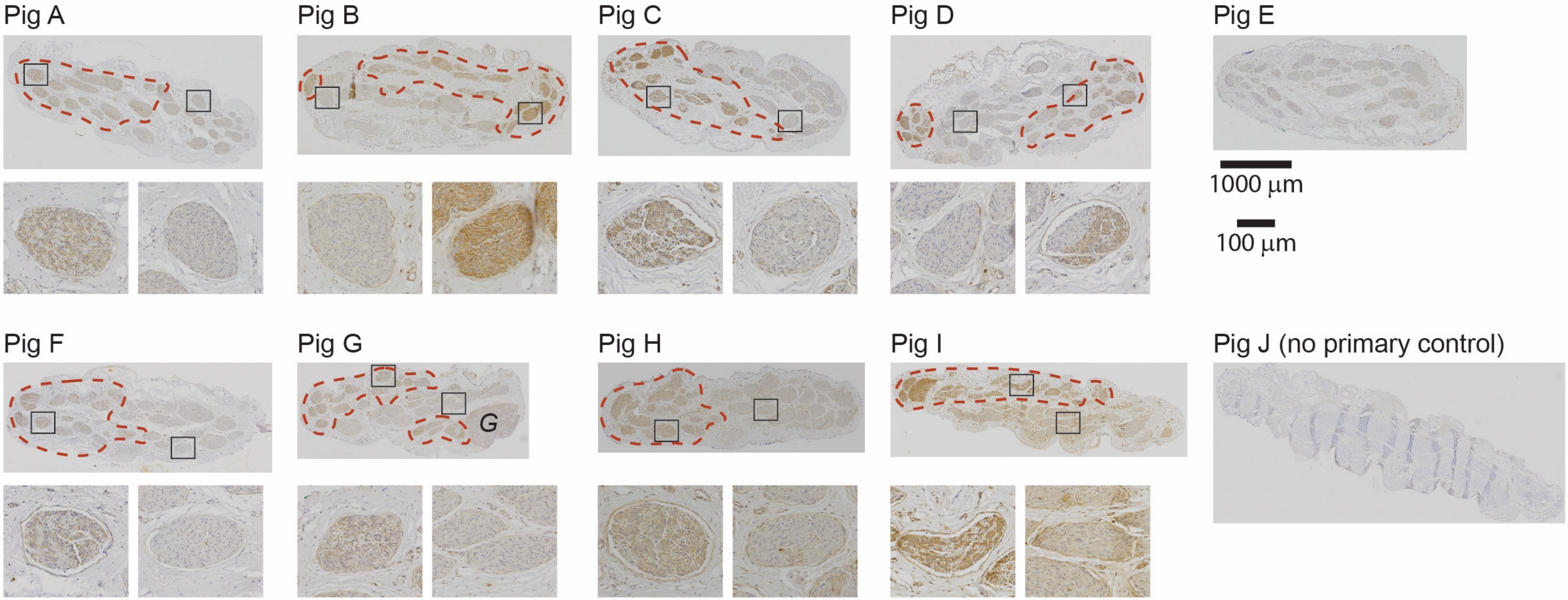

Supplemental Figure 4. Immunohistological cross sections of pig cervical vagus nerve labeled with an antibody against choline acetyltransferase (ChAT), indicated by the brown chromogen (DAB). The red dashed lines approximately delineate the regions with ChAT+ fibers. The black boxes indicate the two zoomed regions for each sample, one showing a fascicle with ChAT+ fibers and the other showing a fascicle without ChAT+ fibers, except for the right-hand zoomed fascicle for pig D, showing half of the fibers as ChAT+. The ChAT labeling in pig E was too pale for analysis. Pig J served as a control, where the sample underwent the same processing as the other samples, with the exclusion of the primary antibody; the vertical purple lines are wrinkles in the section. The “G” on the cross section from pig G indicates a ganglion. All full cross sections correspond to the 1000 μm scale bar; all zoomed views of single fascicle correspond to the 100 μm scale bar.

Subject ID	Age (weeks)	Sex	Body mass (kg)	Use/treatment prior to post-mortem dissection	Distance from carotid bifurcation (cm)	Distance from bottom of jaw to top of sternum (cm)	Time from death to collection (hr)	Days from collection to paraffin embedding
Pig A	13	Female	32.8	Laparotomy training	Not recorded	13	2.25	7
Pig B	13	Female	28.2	Laparotomy training	5	13	2.5	7
Pig C	13	Female	28.7	Laparotomy training	4.25	13	4	7
Pig D	15	Female	45	Laparotomy training	6.25	14	1.25	5
Pig E	14	Female	38.5	Laparotomy training	8	15	1	5
Pig F	14	Female	39.5	Laparotomy training	7.25	16	1.5	5
Pig G	10.5	Male	24.4	Heparinized & perfused with saline (6L) + heparin (10000 units per 10 mL / 1L saline)	6	15	3	5
Pig H	10.5	Male	23.4	4 days with paclitaxel (chemo drug); 0.5 mL/kg each day (clinical dosage); Heparinized & perfused with saline (5L) + heparin (10000 units per 10 mL / 1L saline)	4.75	13	1.5	5
Pig I	10.5	Male	23.8	4 days with paclitaxel (chemo drug); 0.5 mL/kg each day (clinical dosage) + stem cell treatment in intrathecal space (500000 bone marrow stromal cells in 5 mL of saline) 1 day before euthanasia; Heparinized & perfused with saline (5L) + heparin (10000 units per 10 mL / 1L saline)	6	13	1.5	5
Pig J	13	Female	29.6	Trauma training	8.5	13	3.5	8

Supplemental Table 1. Metadata for pig vagus nerve samples used for immunohistochemistry. The "Distance from carotid bifurcation" column provides the distance from the center of the sample (from which we sectioned) to the "valley" of the carotid bifurcation. See Methods for the description of the "Distance from bottom of jaw to top of sternum".
