# Supplementary material for: Functional vagotopy in the cervical vagus nerve of the domestic pig: implications for the study of vagus nerve stimulation
Source: J Neural Eng. Author manuscript; Available in PMC 2020 Jun 21. (PMC7306215; doi:10.1088/1741-2552/ab7ad4)
Supplement: supplementary information 2 [file NIHMS1594881-supplement-supplementary_information_2.pdf]

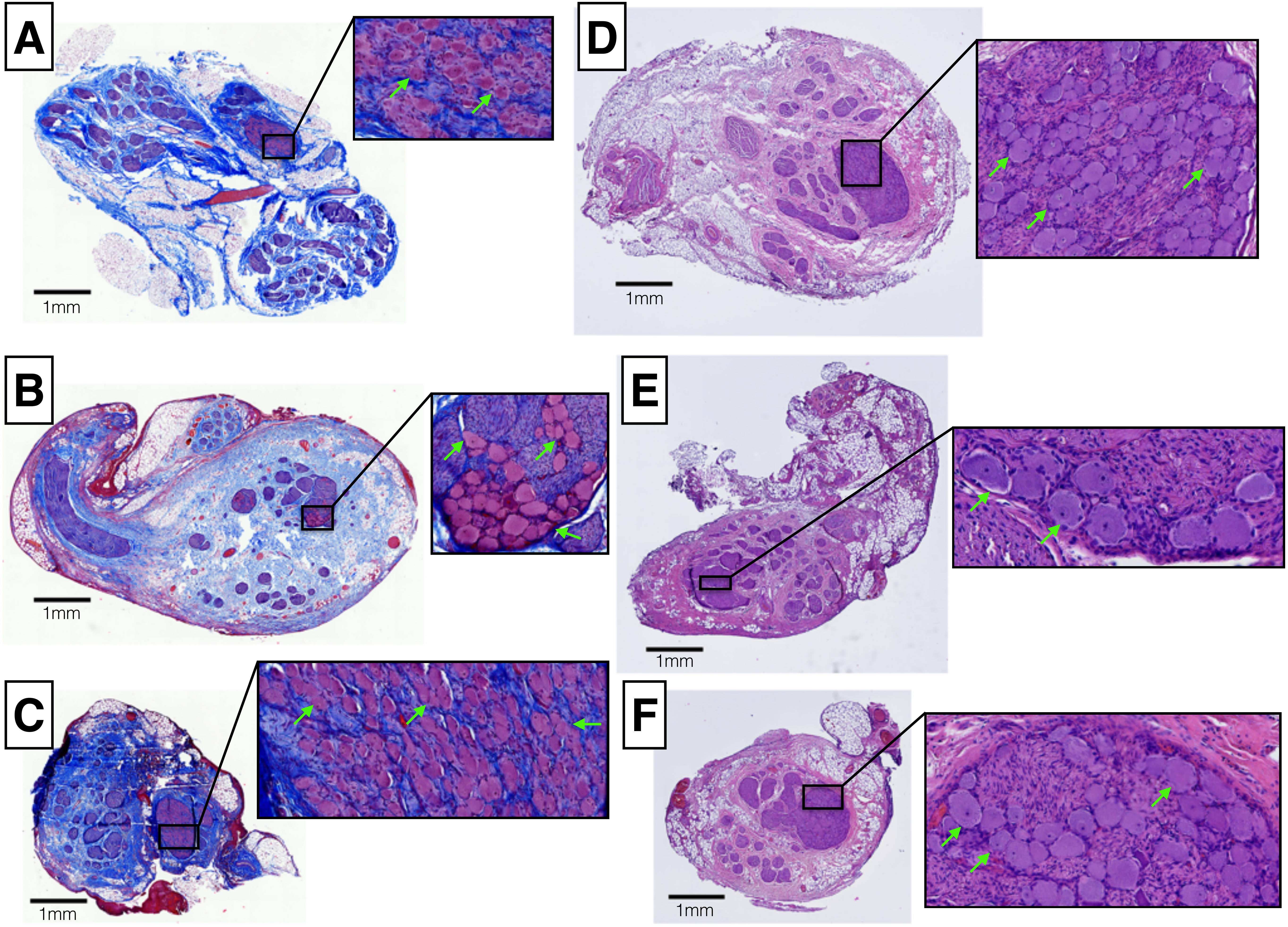
A
1mm
D
1mm
B
1mm
E
1mm
C
1mm
F
1mm

Supplemental Figure 1: Six subjects had vagal nerve sections removed as discussed in methods. These subjects were more sparsely sampled and therefore the pseudo-unipolar cells are visible, but the aggregated plane with the vast majority were not identified. Subjects A, B and C were stained with Gomori's trichrome. Subjects D, E, and F were completed during pilot studies, and stained with H & E. Green arrows indicate pseudo-unipolar cells with surrounding satellite cells.

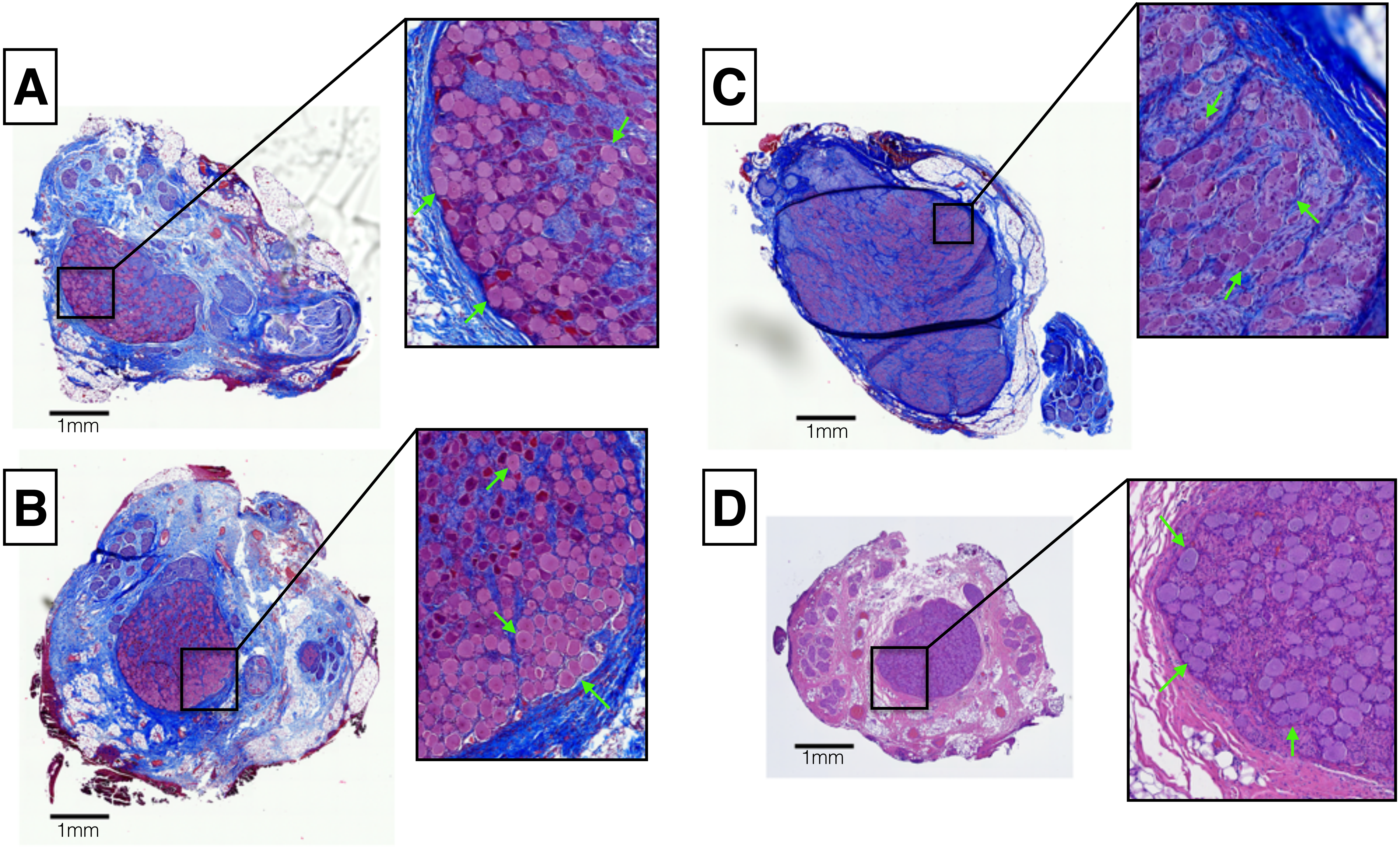
A
1mm
B
1mm
C
1mm
D
1mm

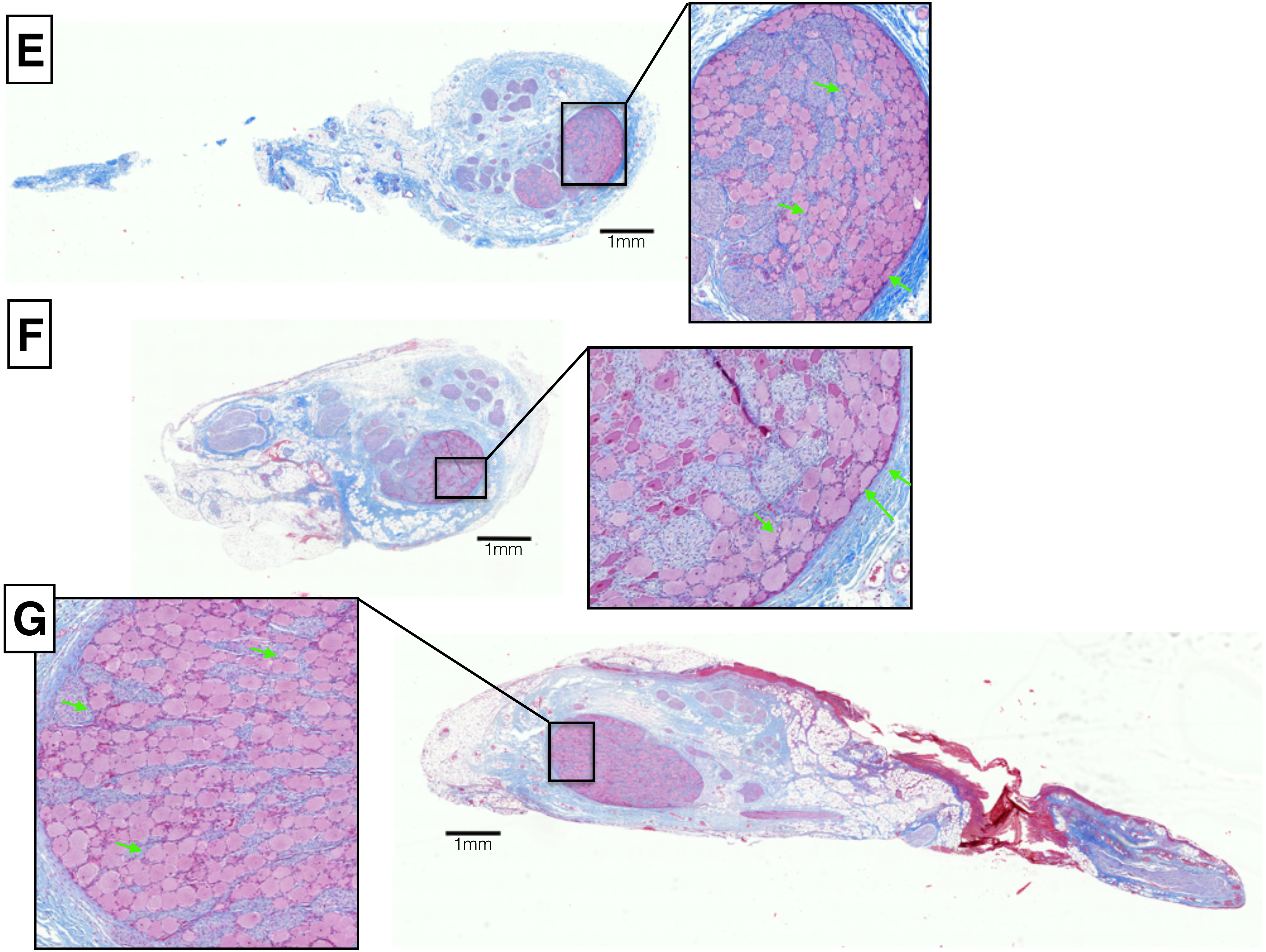
E
1mm
F
1mm
G
1mm

Supplemental Figure 2: Six subjects (in addition to those presented in the manuscript) had vagal nerve sections removed as discussed in methods. In these subjects we were able to locate the pseudo-unipolar cells in an aggregated plane. Subjects A, B, C, E and F were stained with Gomori's trichrome. Subject D was completed during pilot studies, and stained with Hematoxylin & Eosin. Green arrows indicate pseudo-unipolar cells with surrounding satellite cells.

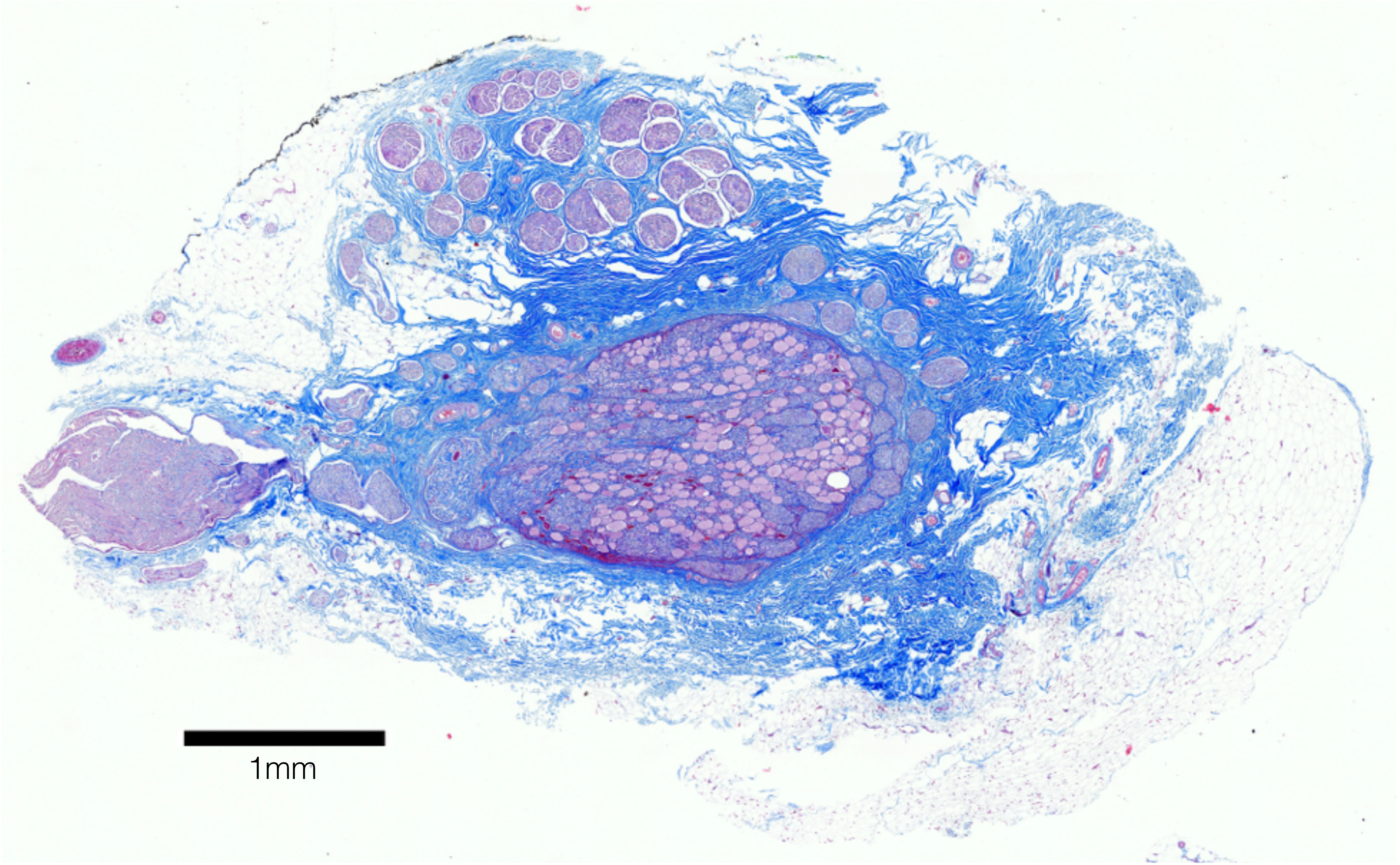

Supplemental Figure 3: As shown in this representative animal, current vagal nerve studies extending to miniature pigs have shown similar organization in the aggregation of pseudo-unipolar cells at the level of the nodose ganglia. Data has been collected in an additional five miniature pigs, and is currently being analyzed.
