# Supplementary material for: Functional vagotopy in the cervical vagus nerve of the domestic pig: implications for the study of vagus nerve stimulation
Source: J Neural Eng. Author manuscript; Available in PMC 2020 Jun 21. (PMC7306215; doi:10.1088/1741-2552/ab7ad4)
Supplement: supplementary information 3 [file NIHMS1594881-supplement-supplementary_information_3.pdf]

Scale: 1894 pixels/mm

Subject 2 Record_P818

Nodose

P818_LV4

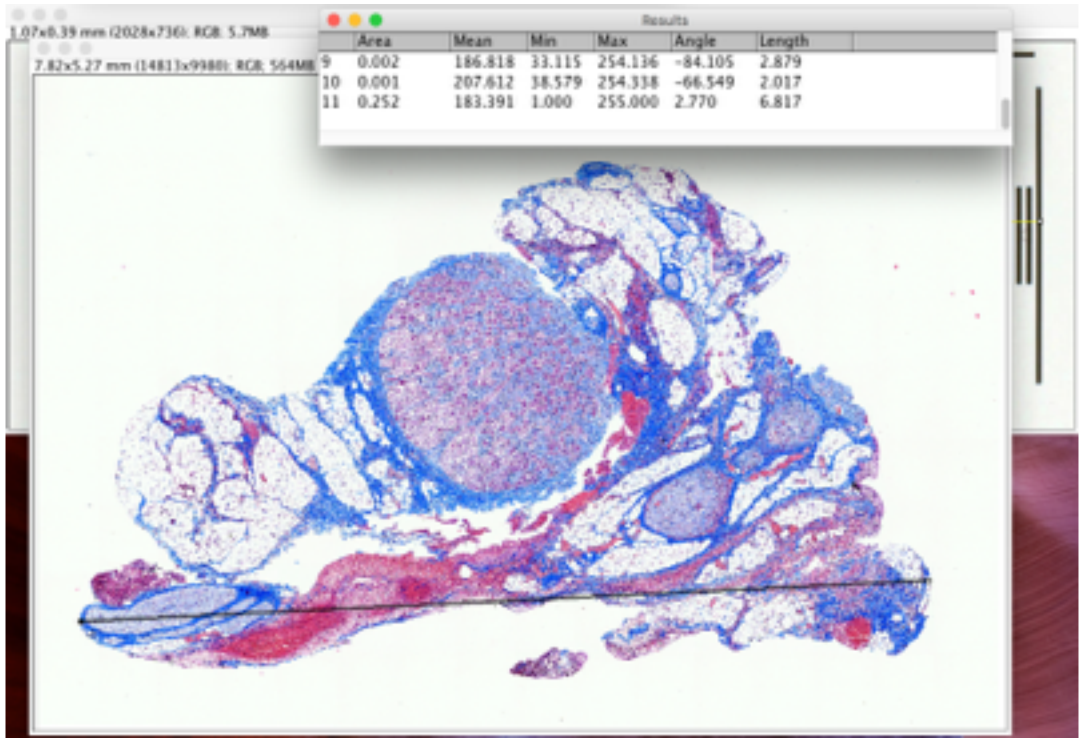

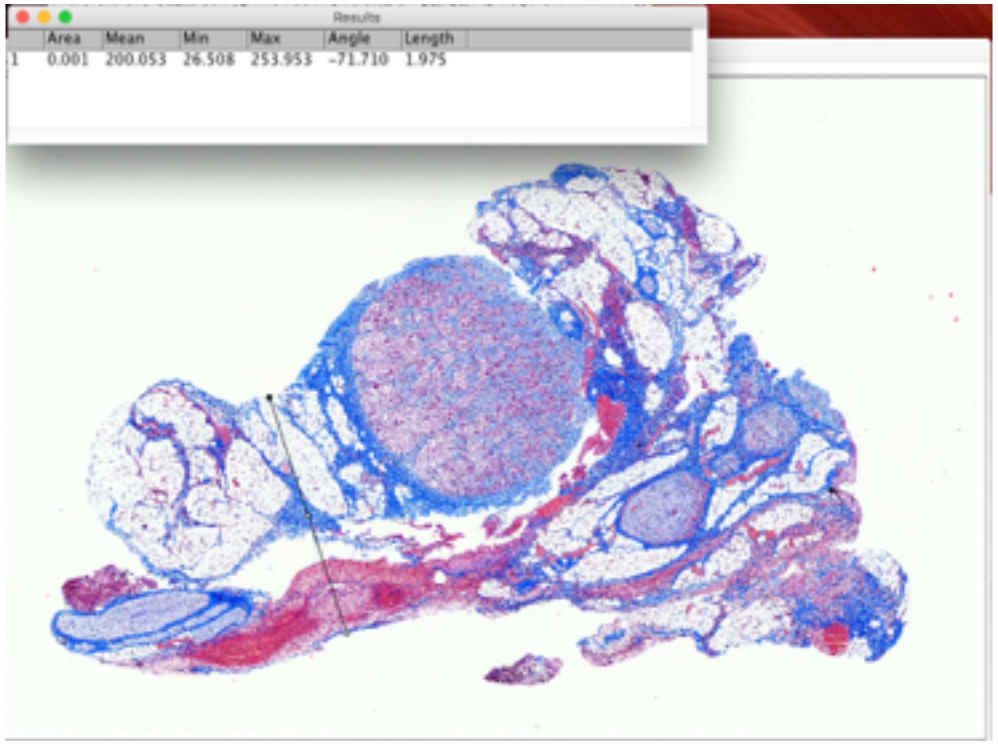

widest and narrowest diameter

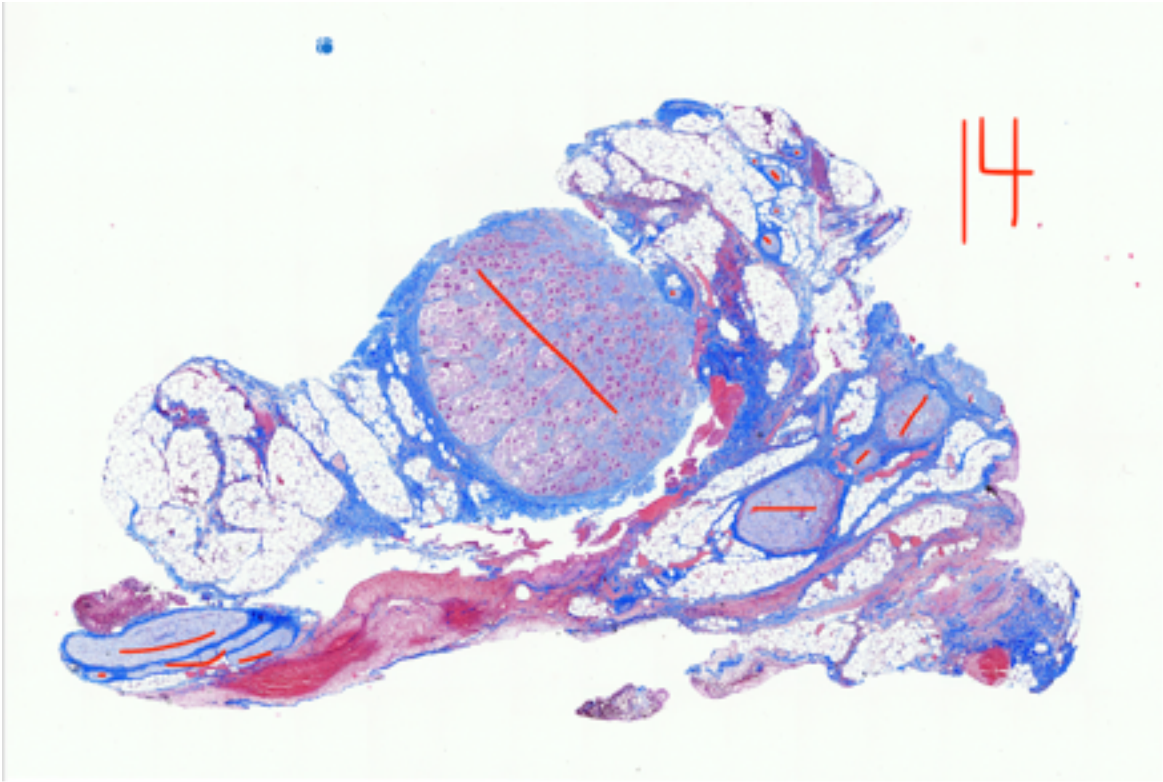

Fascicle count, 14

Nodose

P818_LV4

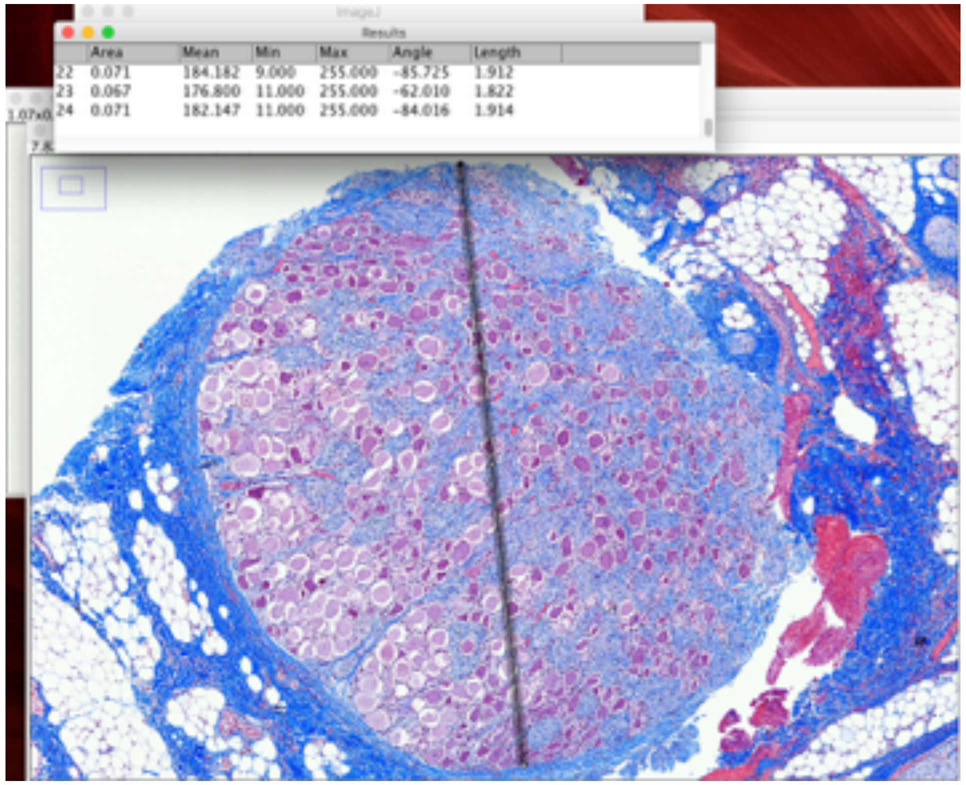

	Area	Mean	Min	Max	Angle	Length
22	0.071	184.182	9.000	255.000	-85.725	1.912
23	0.067	176.800	11.000	255.000	-62.010	1.822
24	0.071	182.147	11.000	255.000	-84.016	1.914

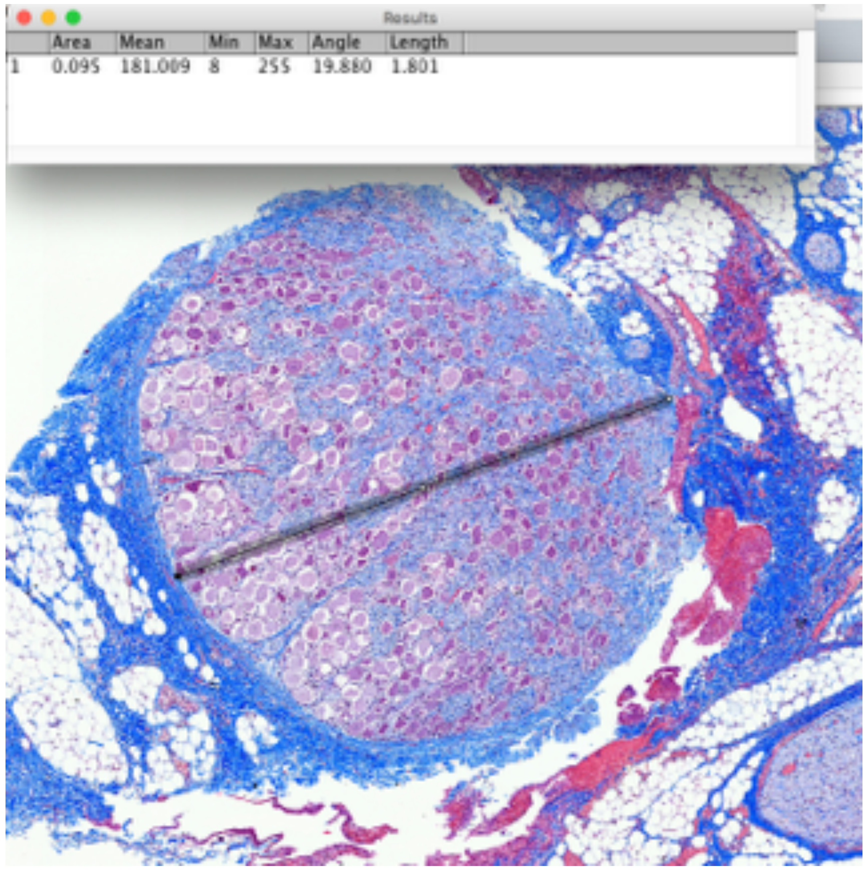

	Area	Mean	Min	Max	Angle	Length
1	0.095	181.009	8	255	19.880	1.801

Largest Fascicle_widest and narrowest diameter

Mid-VN

P818_LV5b

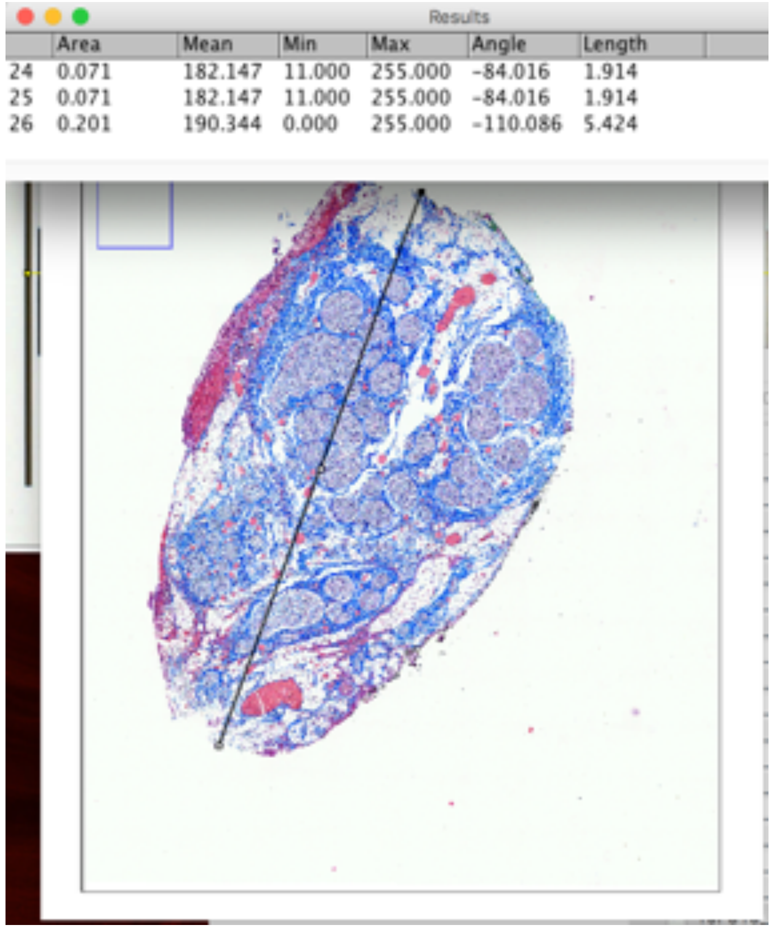

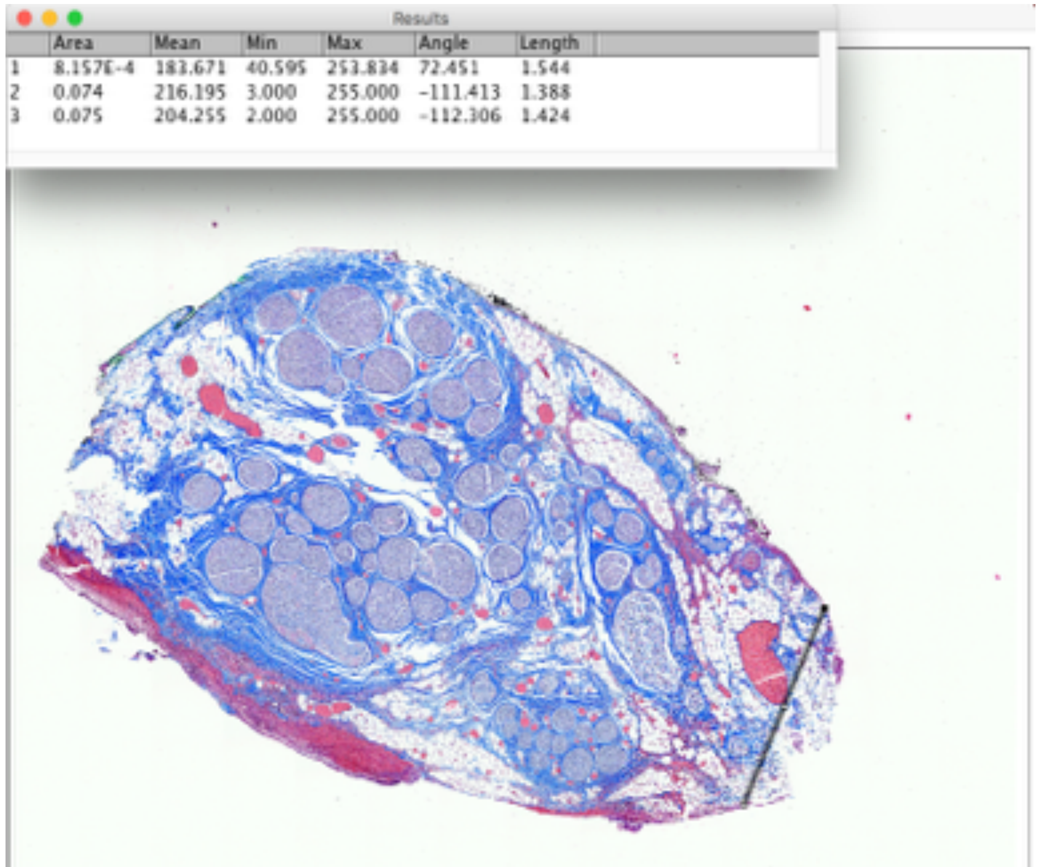

widest and narrowest diameter

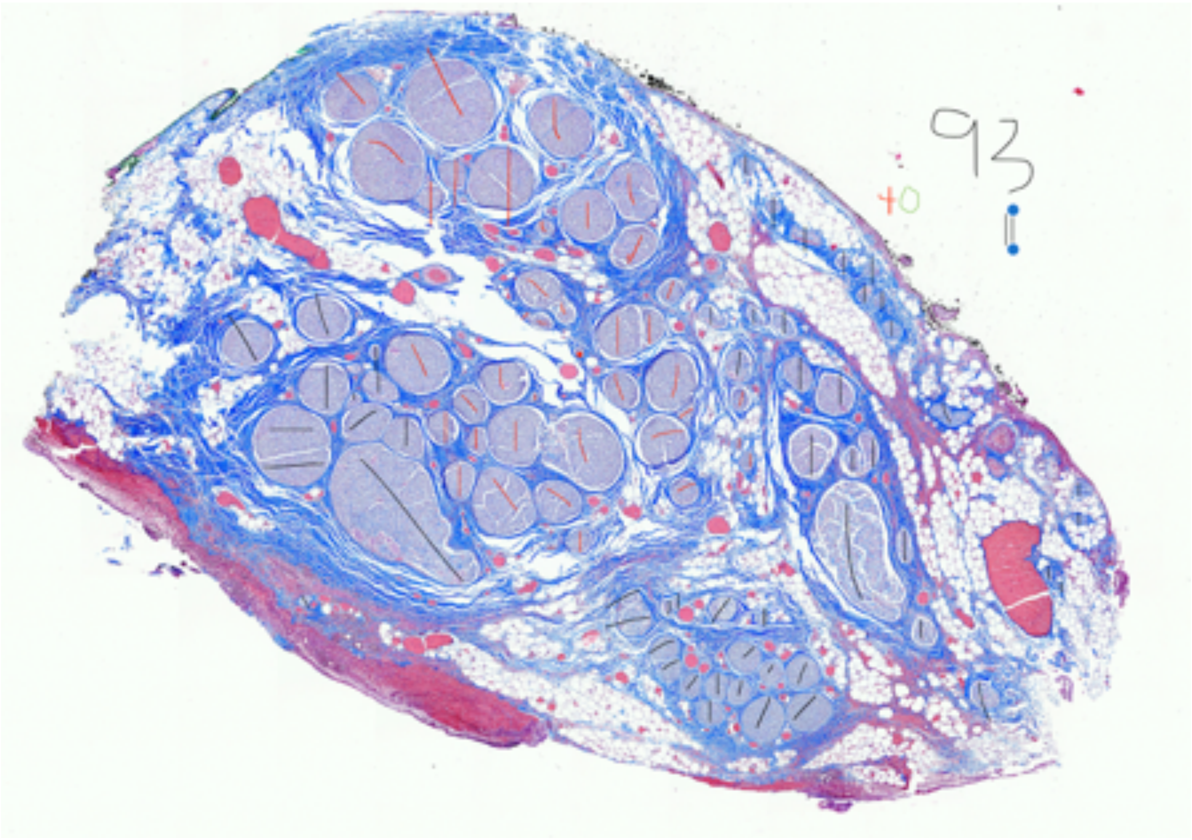

Fascicle count, 95

Mid-VN

P818_LV5b

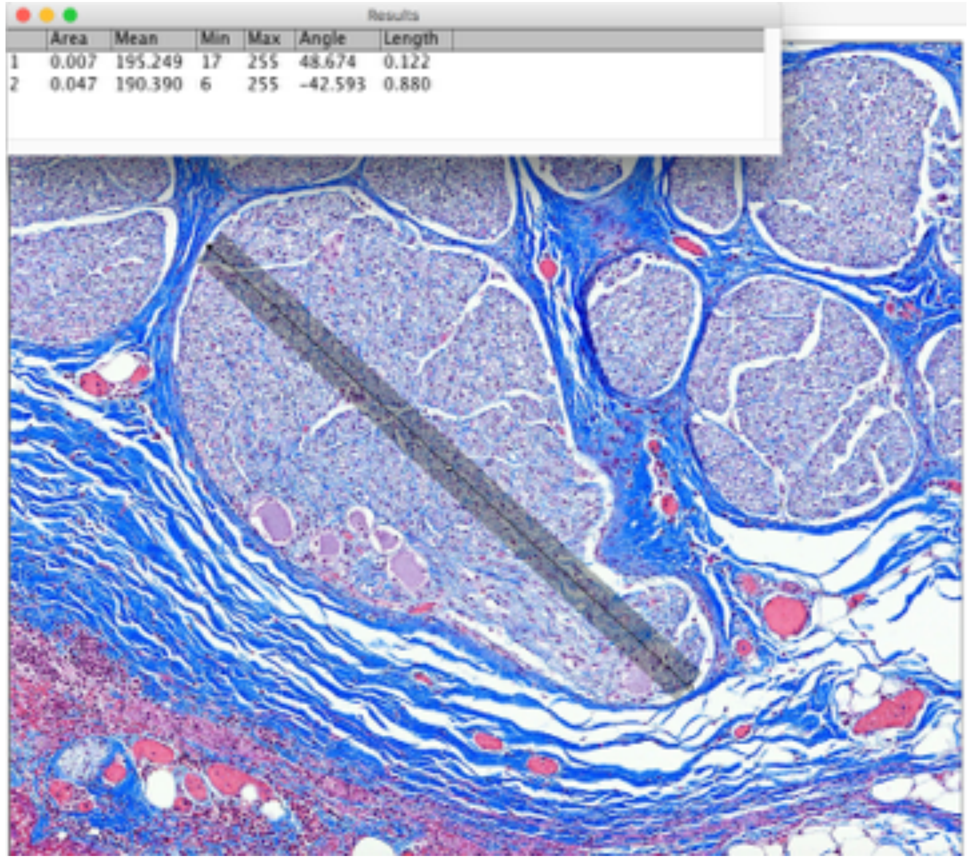

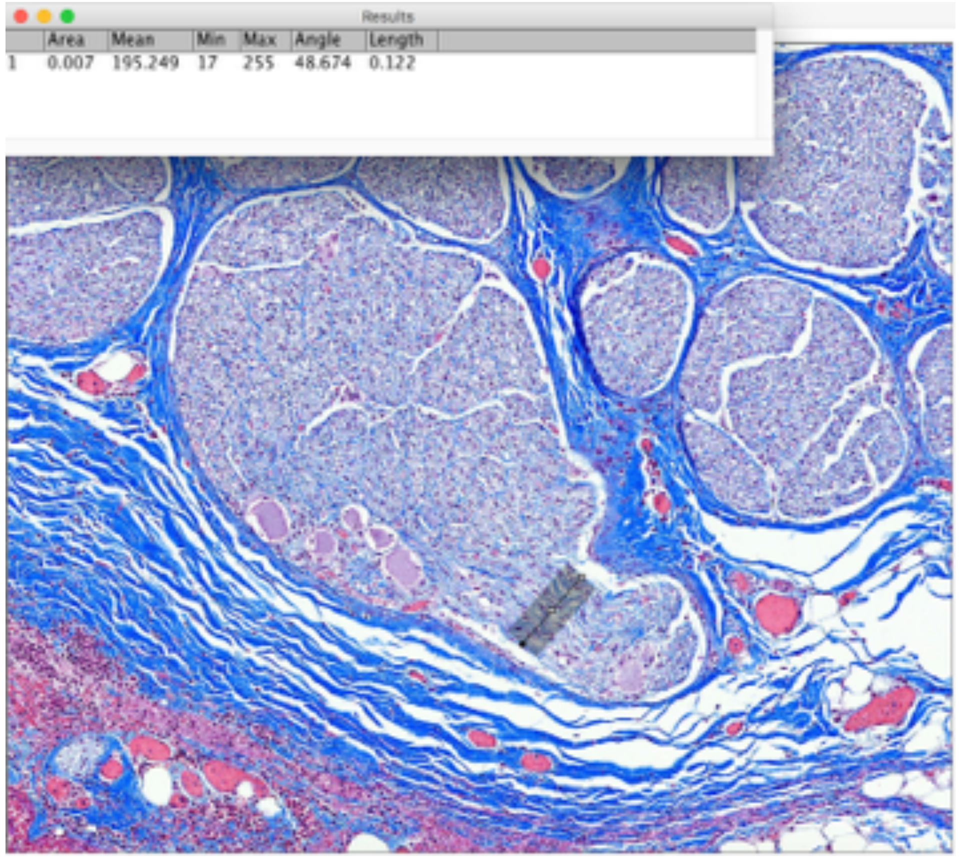

Largest Fascicle_widest and narrowest diameter

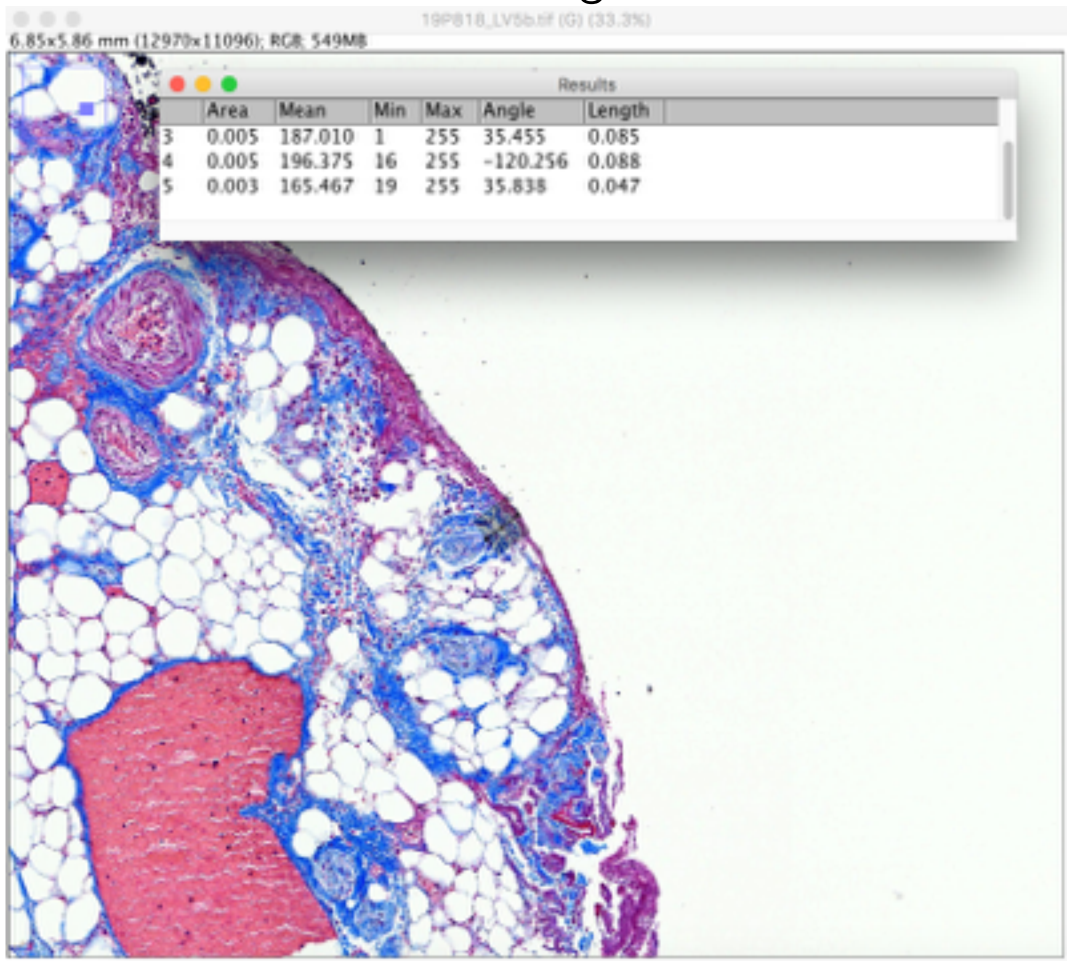

fascicle depth

Subject 6_P839

Pre-Nodose

P839_Nodose_4

Widest and narrowest diameters

Results

	Area	Mean	Min	Max	Angle	Length
31	0.206	203.180	30.000	255.000	-105.933	5.508
32	0.110	180.568	28.000	255.000	-9.408	2.946
33	0.166	163.476	0.000	255.000	-98.655	4.435

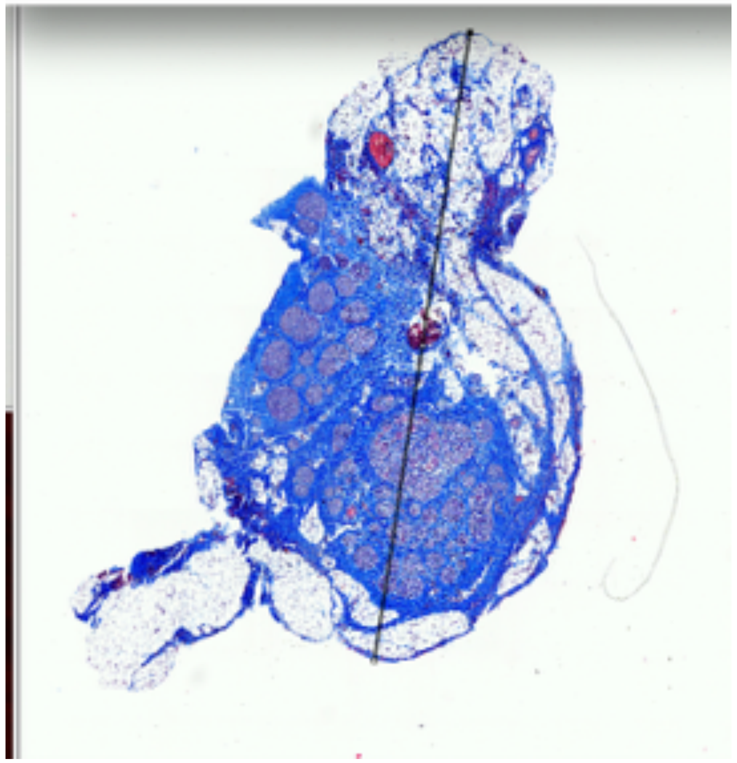

Results

	Area	Mean	Min	Max	Angle	Length
4	0.005	196.375	16	255	-120.256	0.088
5	0.003	165.467	19	255	35.838	0.047
6	0.072	148.915	3	255	-0.796	1.369

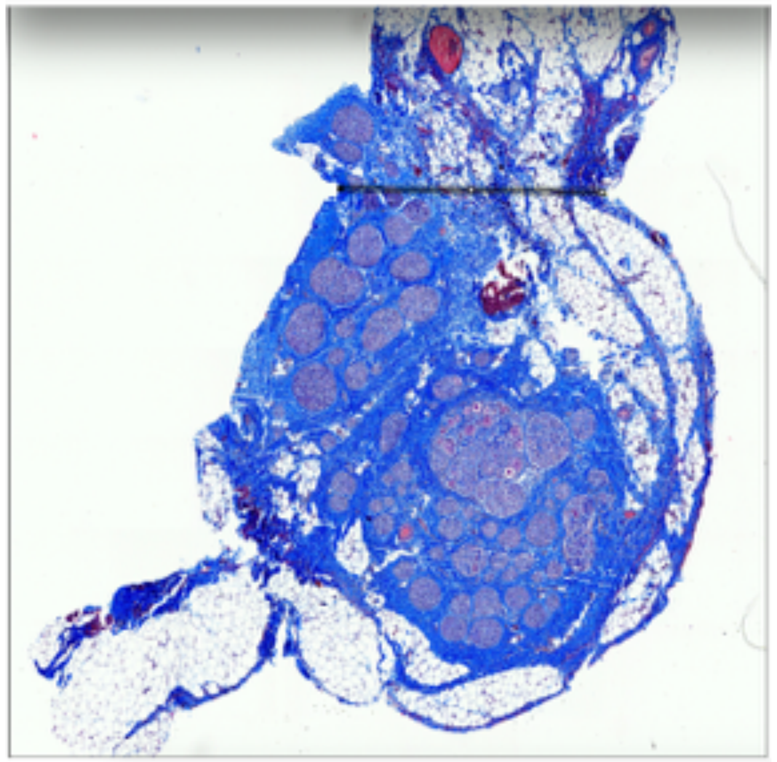

Largest fascicle _widest and narrowest diameters

Results

	Area	Mean	Min	Max	Angle	Length
33	0.166	163.476	0.000	255.000	-98.655	4.435
34	0.095	140.053	7.000	255.000	-27.154	2.573
35	0.019	151.138	7.000	255.000	47.222	0.520

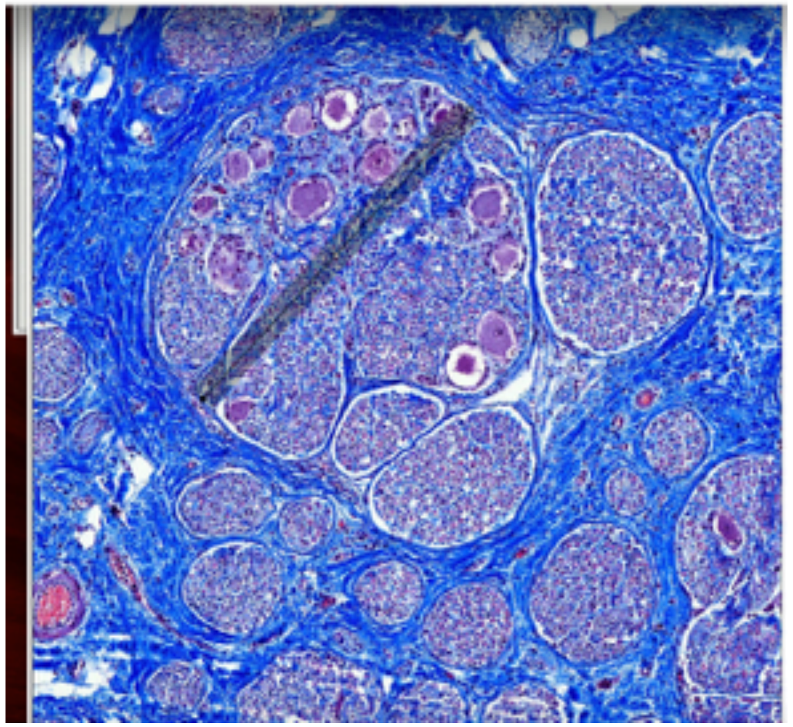

Results

	Area	Mean	Min	Max	Angle	Length
2	0.011	154.088	3	255	-48.180	0.215
3	0.011	154.088	3	255	-48.180	0.215
4	0.018	146.596	15	255	-71.346	0.350

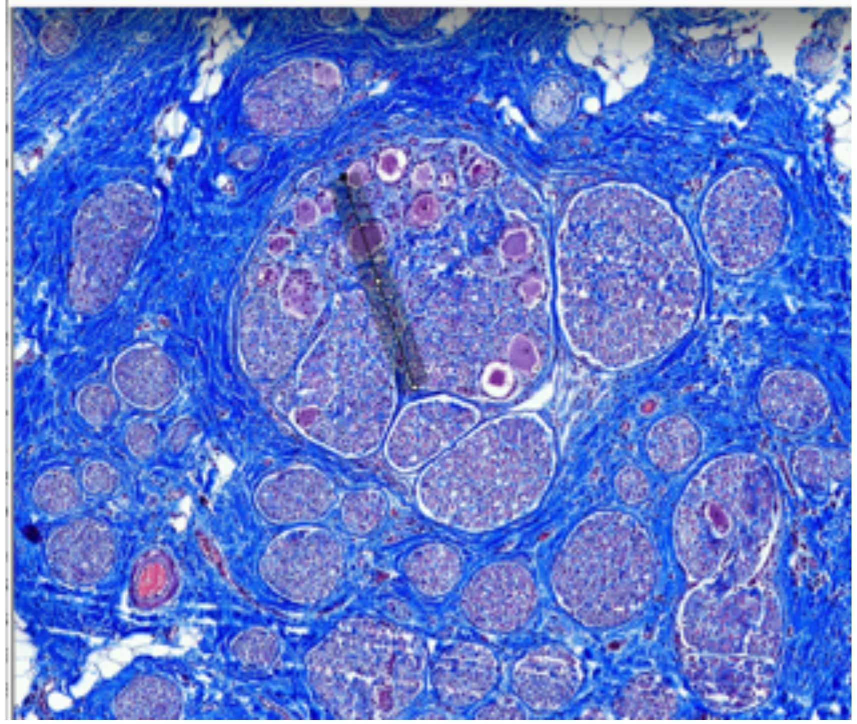

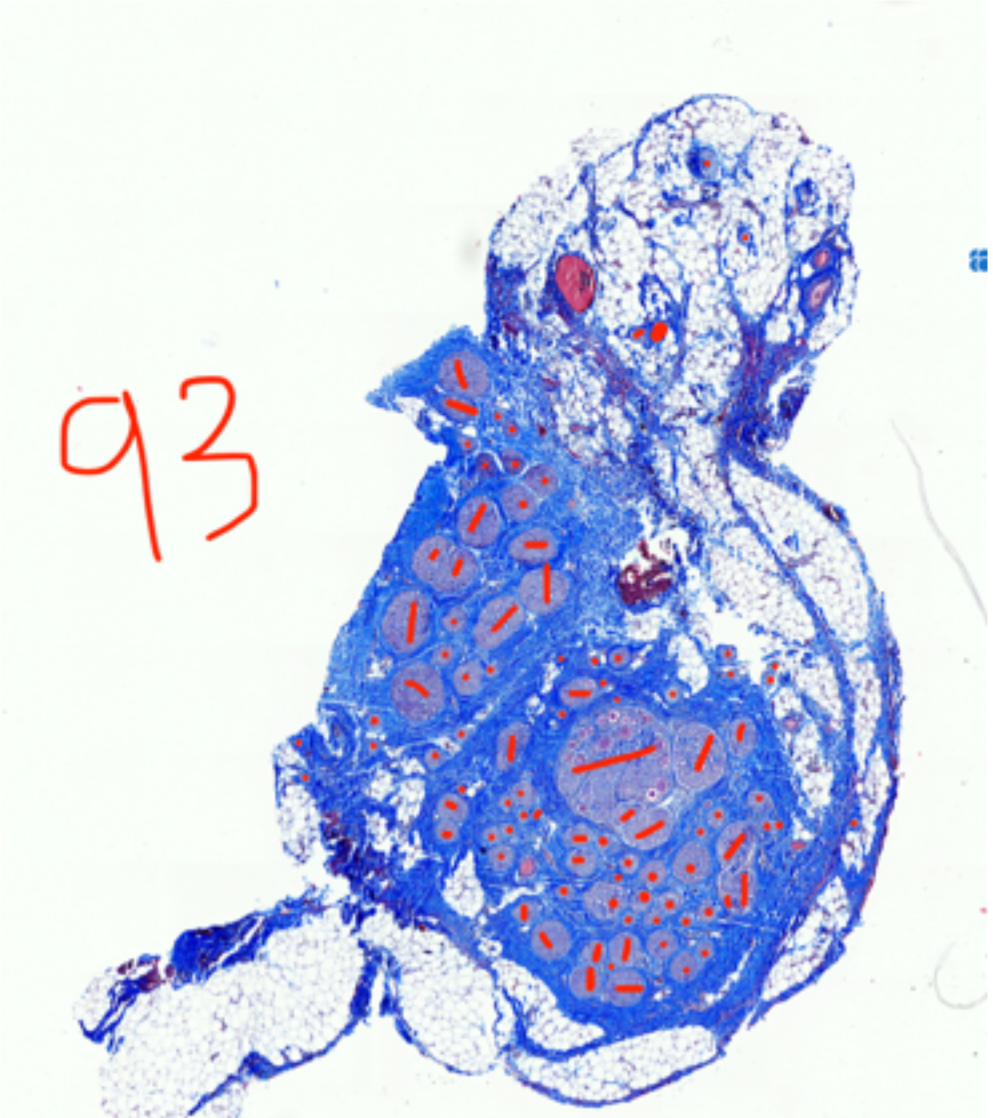

Fascicle count, 93

Widest and narrowest diameters

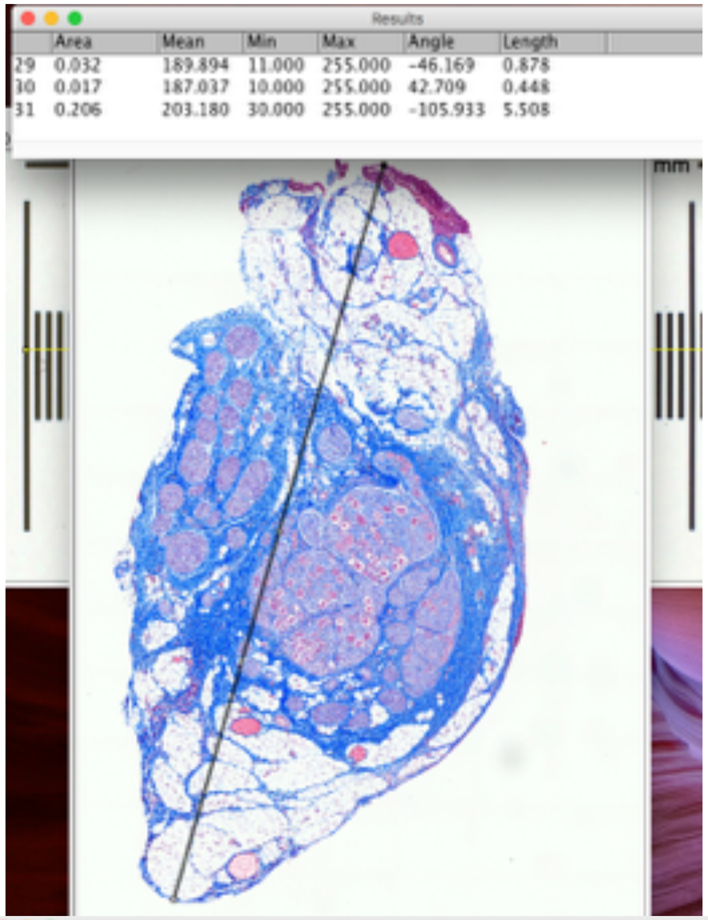

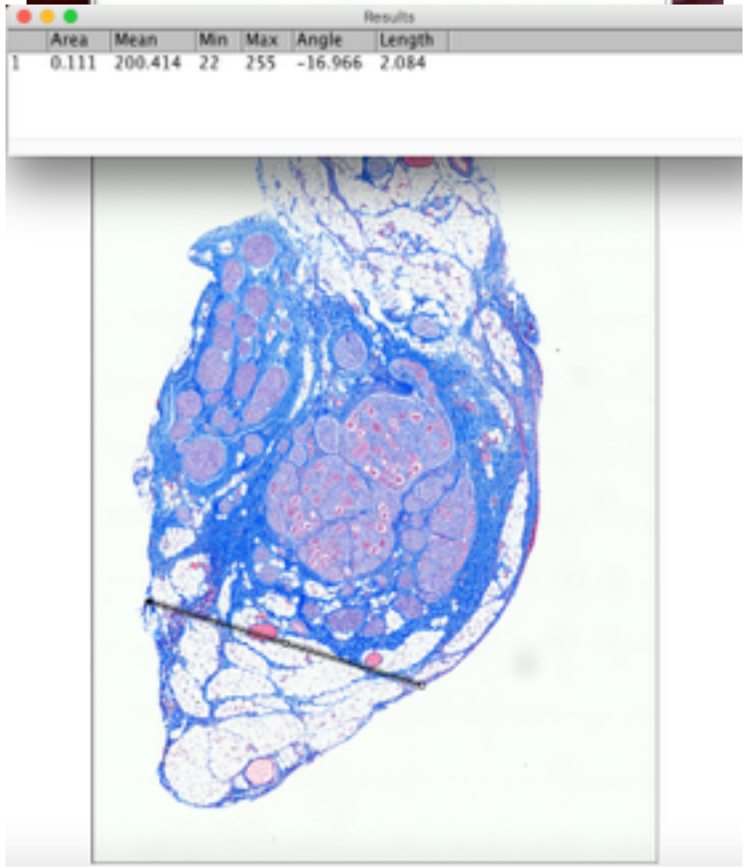

Largest Fascicle_Widest and narrowest diameters

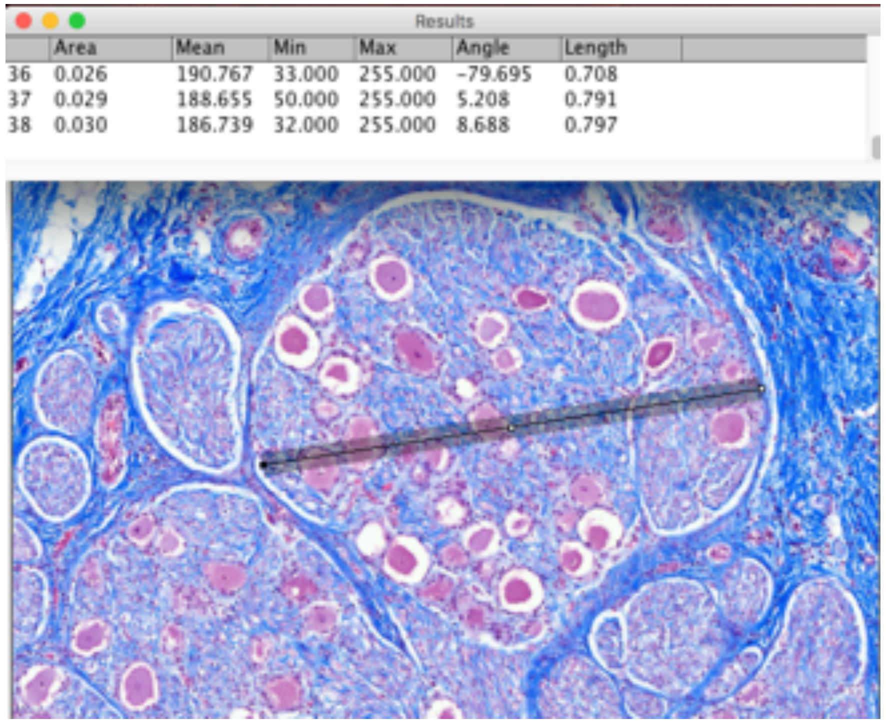

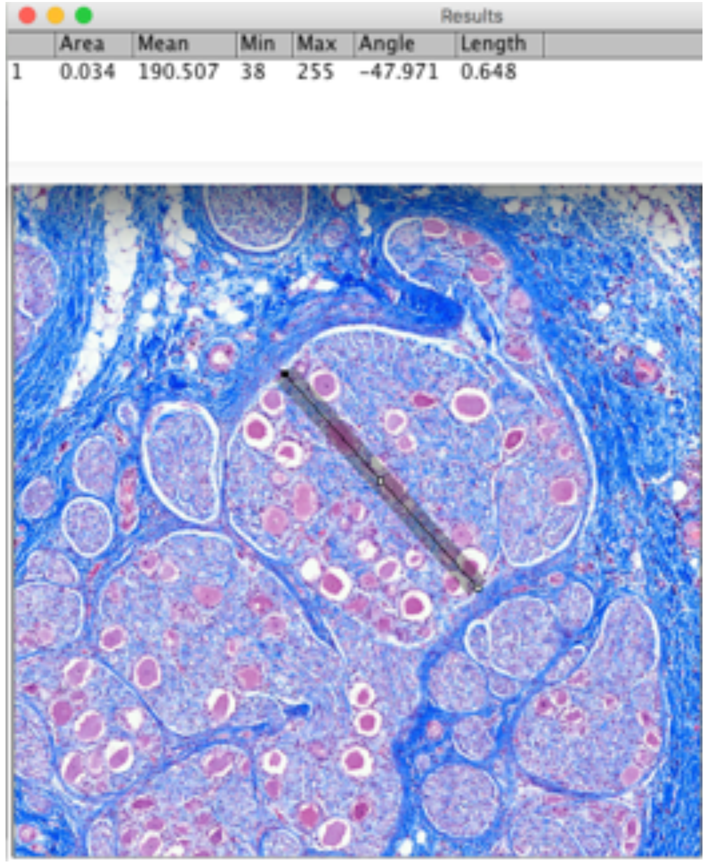

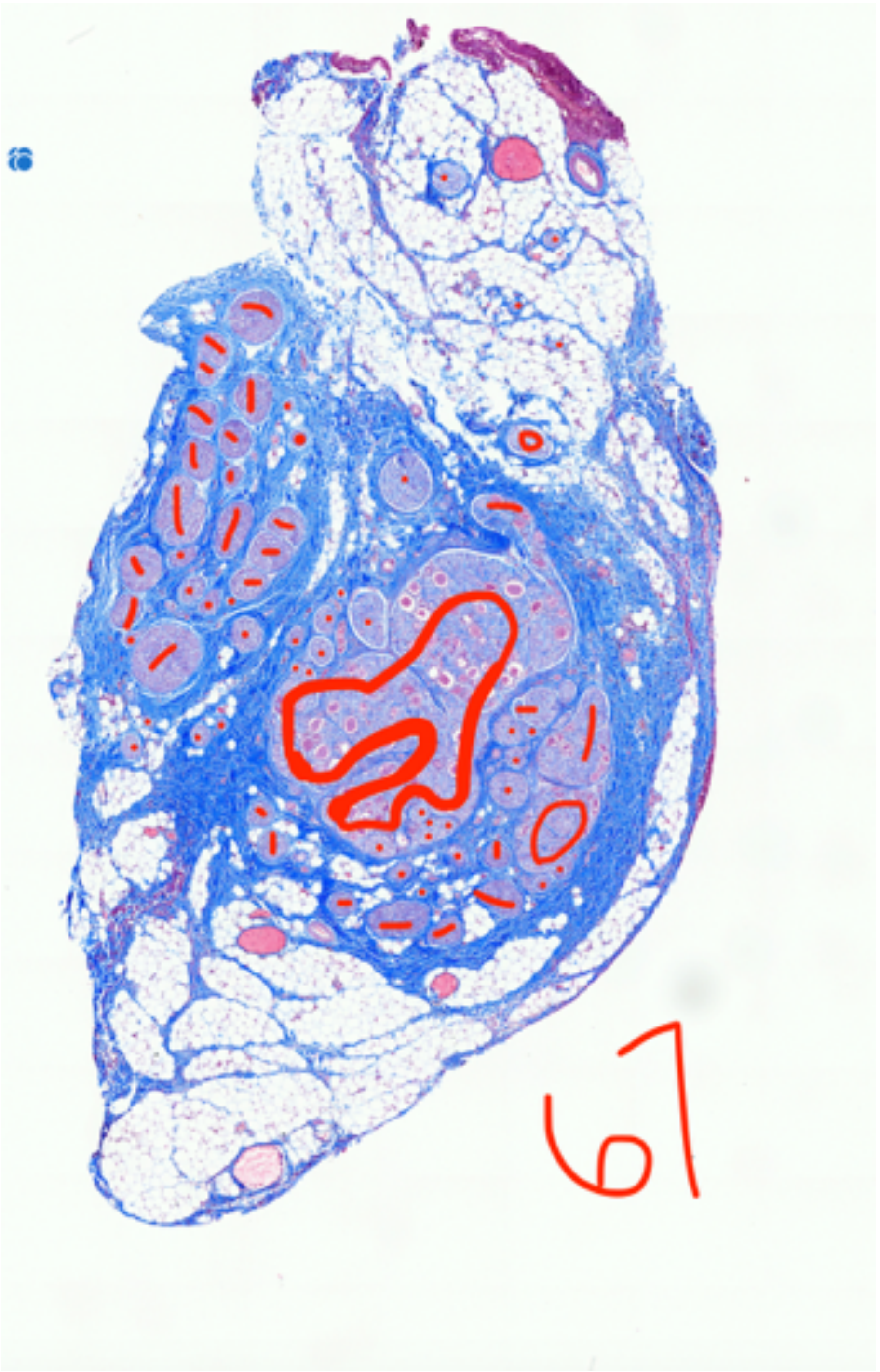

Fascicle count, 67

Subject 7_P845

Pre-nodose

P845_Nodose_SL_16

widest and narrowest diameters

Results

	Area	Mean	Min	Max	Angle	Length
39	0.011	179.848	8.000	255.000	-83.949	0.301
40	0.015	174.962	8.000	255.000	19.592	0.397
41	0.125	167.558	8.000	255.000	-95.077	3.365

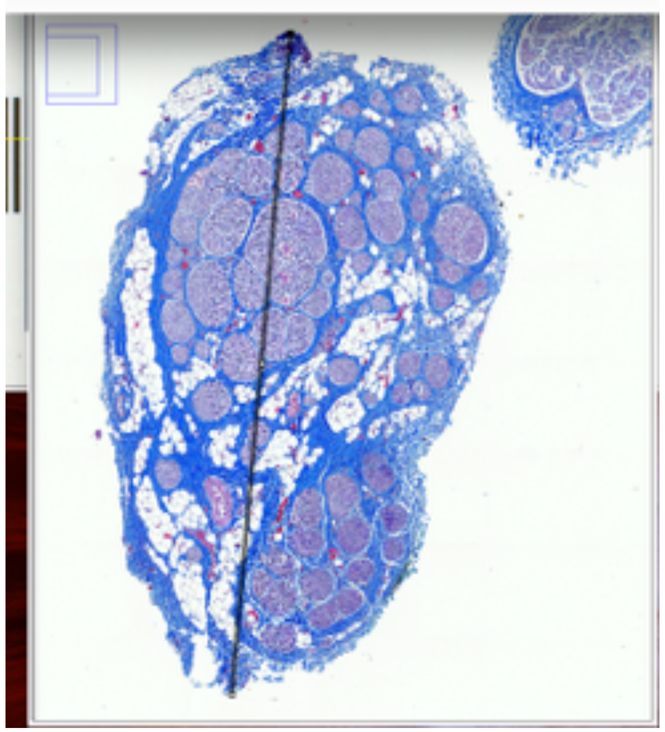

Results

	Area	Mean	Min	Max	Angle	Length
1	0.081	168.558	6	255	-2.369	1.533

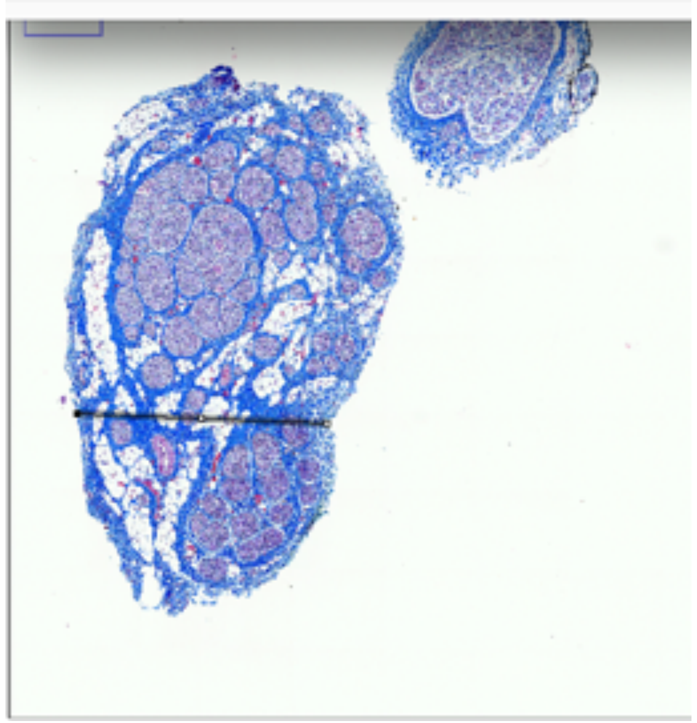

Fascicle count, 94

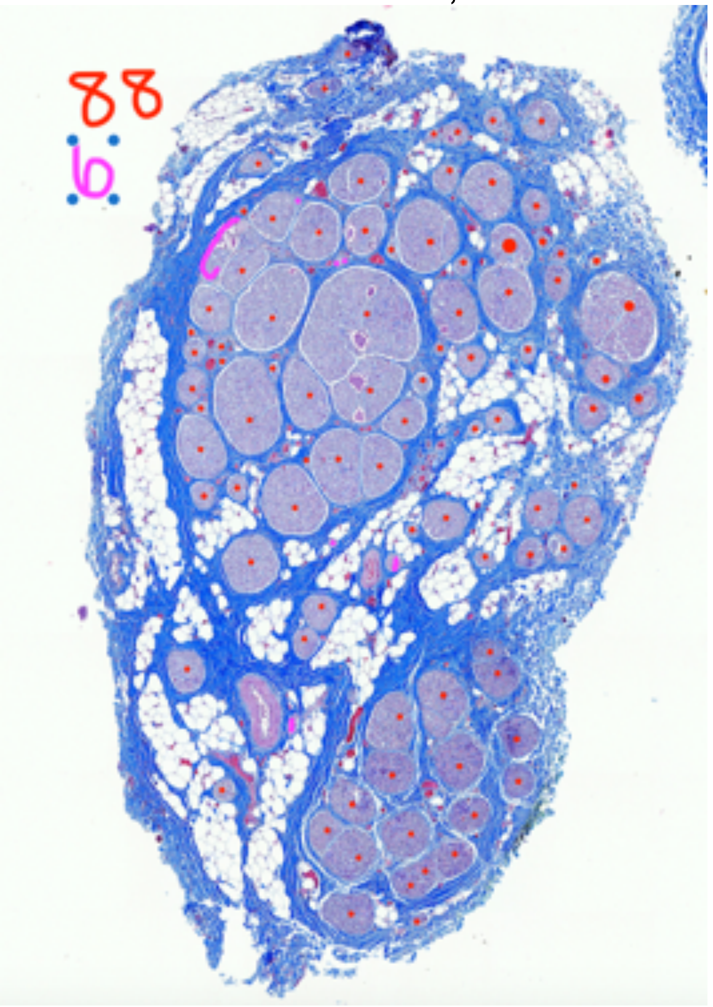

Results

	Area	Mean	Min	Max	Angle	Length
37	0.029	188.655	50.000	255.000	5.208	0.791
38	0.030	186.739	32.000	255.000	8.688	0.797
39	0.011	179.848	8.000	255.000	-83.949	0.301

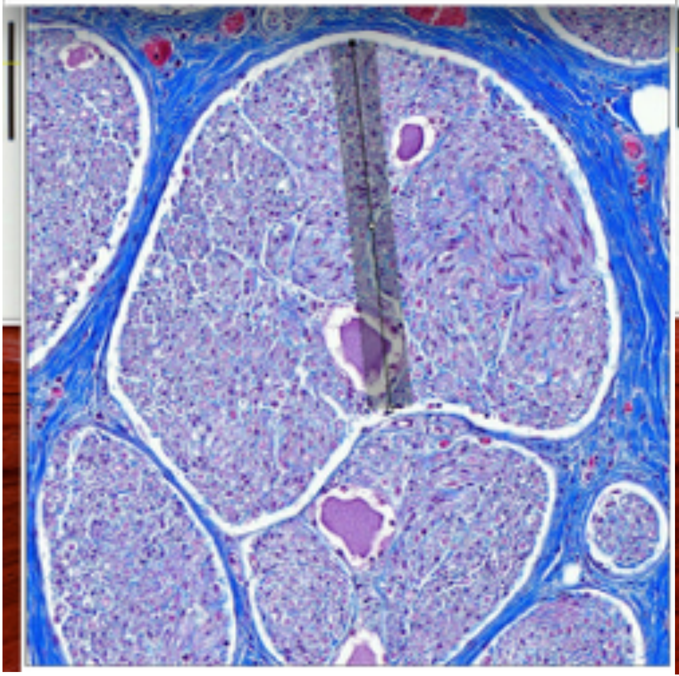

Results

	Area	Mean	Min	Max	Angle	Length
38	0.030	186.739	32.000	255.000	8.688	0.797
39	0.011	179.848	8.000	255.000	-83.949	0.301
40	0.015	174.962	8.000	255.000	19.592	0.397

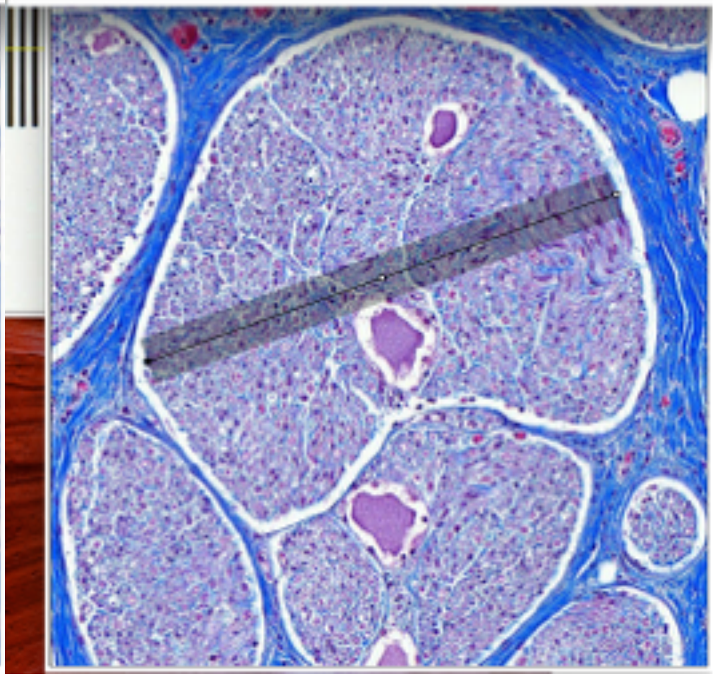

Largest fascicle_widest and narrowest diameters

Mid-VN

P845_VagusA_3_Cranial contact

widest and narrowest diameters

Fascicle Count, 52

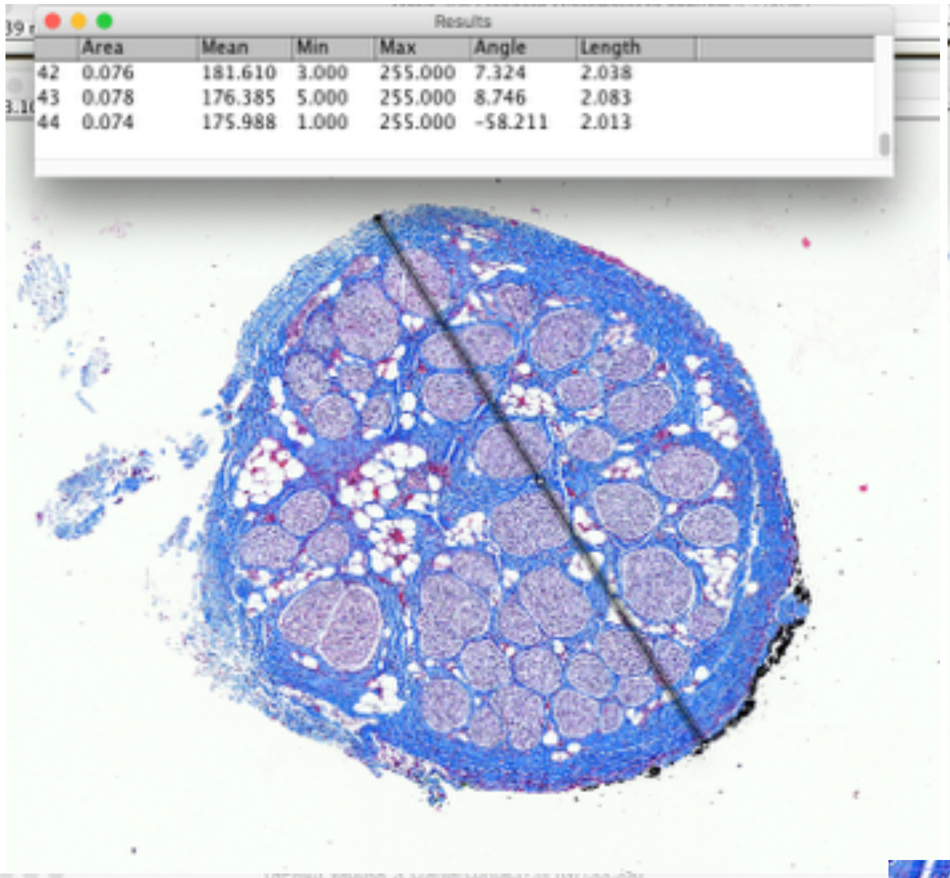

	Area	Mean	Min	Max	Angle	Length
42	0.076	181.610	3.000	255.000	7.324	2.038
43	0.078	176.385	5.000	255.000	8.746	2.083
44	0.074	175.988	1.000	255.000	-58.211	2.013

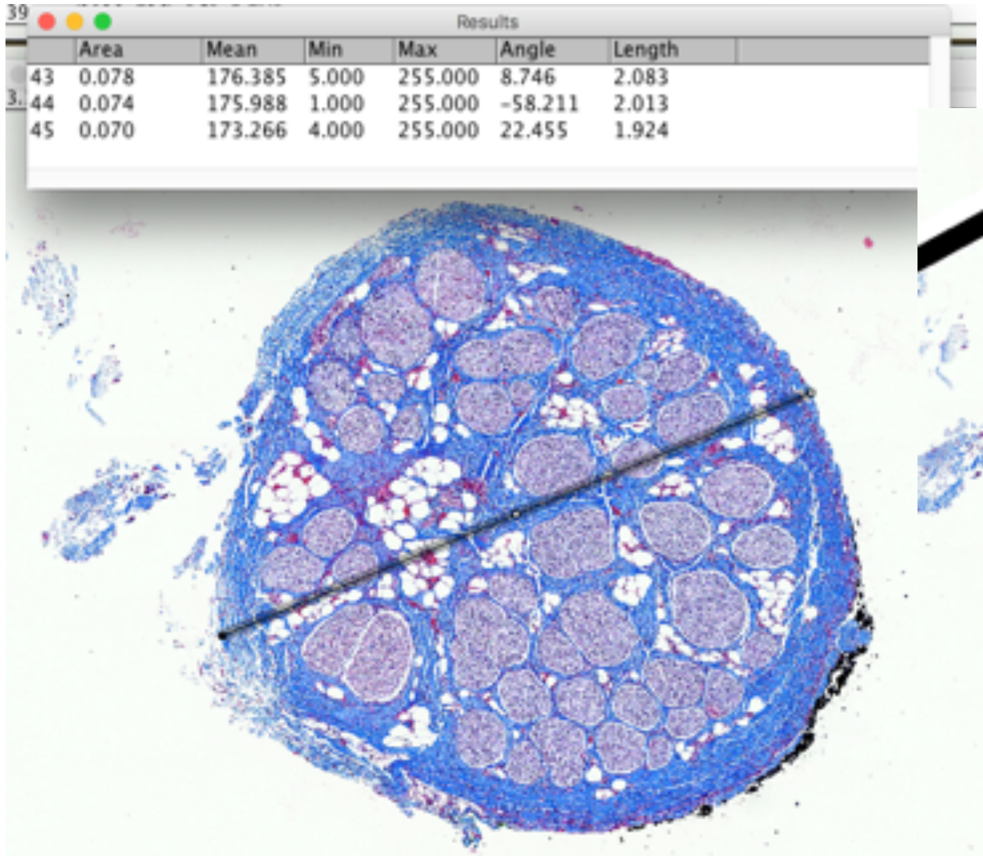

	Area	Mean	Min	Max	Angle	Length
43	0.078	176.385	5.000	255.000	8.746	2.083
44	0.074	175.988	1.000	255.000	-58.211	2.013
45	0.070	173.266	4.000	255.000	22.455	1.924

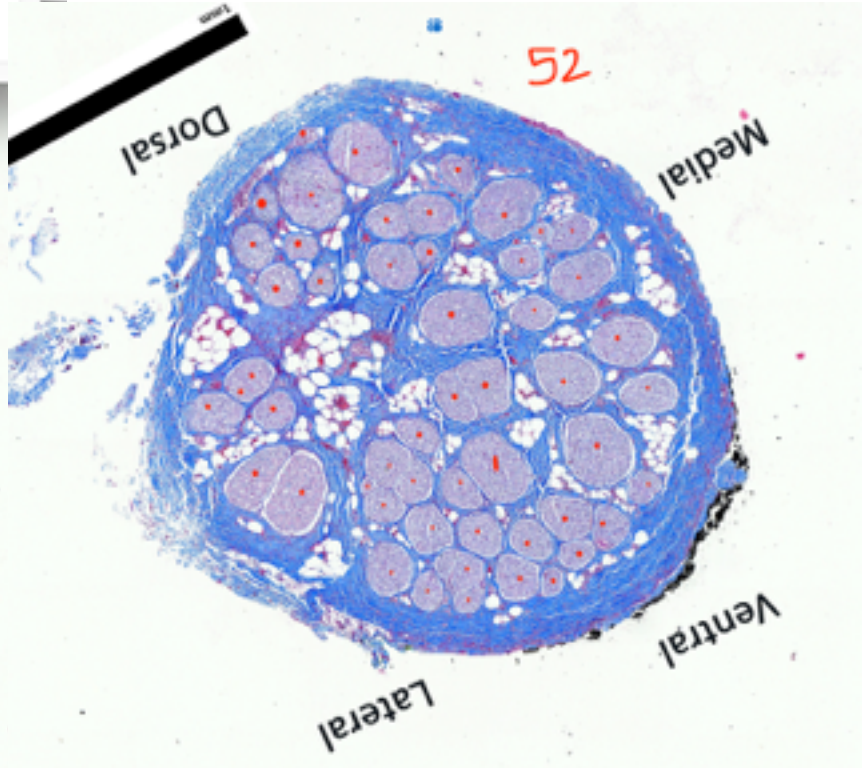

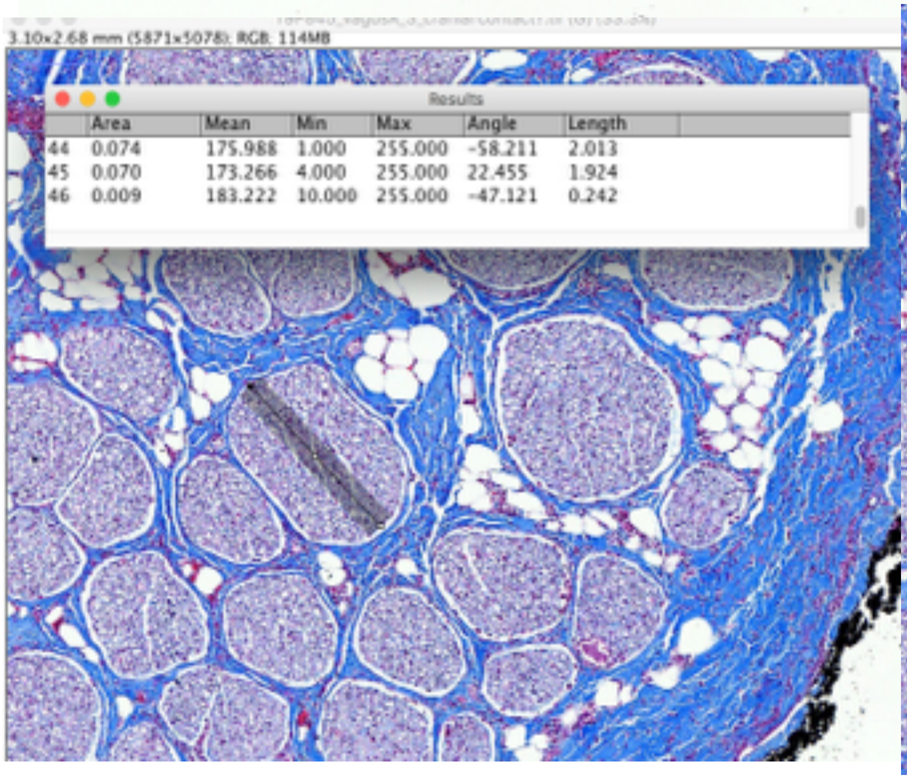

	Area	Mean	Min	Max	Angle	Length
44	0.074	175.988	1.000	255.000	-58.211	2.013
45	0.070	173.266	4.000	255.000	22.455	1.924
46	0.009	183.222	10.000	255.000	-47.121	0.242

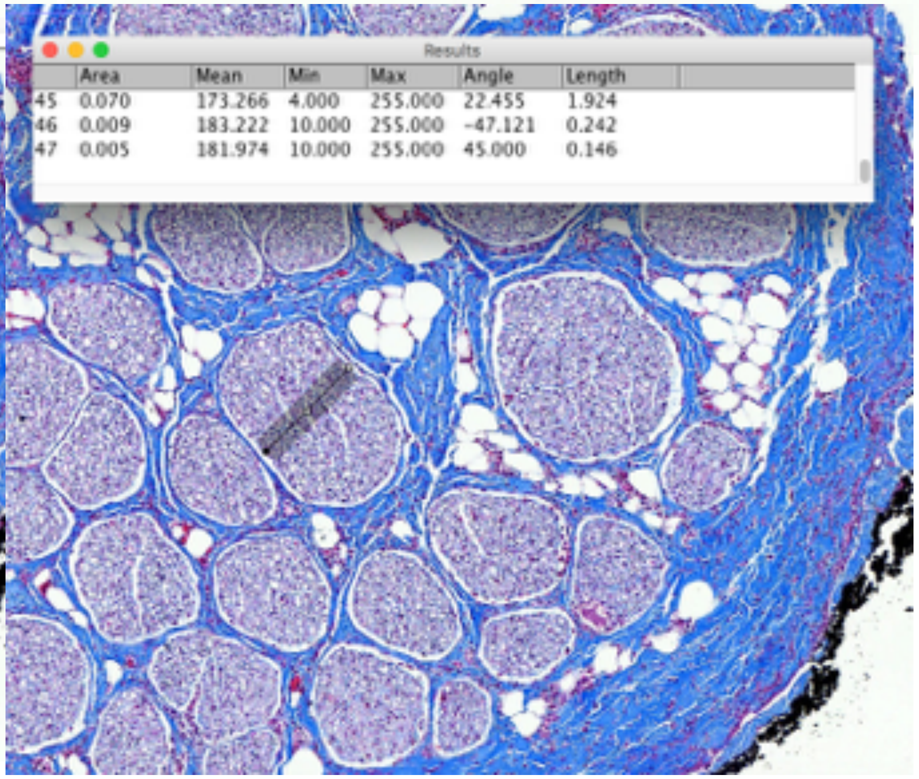

	Area	Mean	Min	Max	Angle	Length
45	0.070	173.266	4.000	255.000	22.455	1.924
46	0.009	183.222	10.000	255.000	-47.121	0.242
47	0.005	181.974	10.000	255.000	45.000	0.146

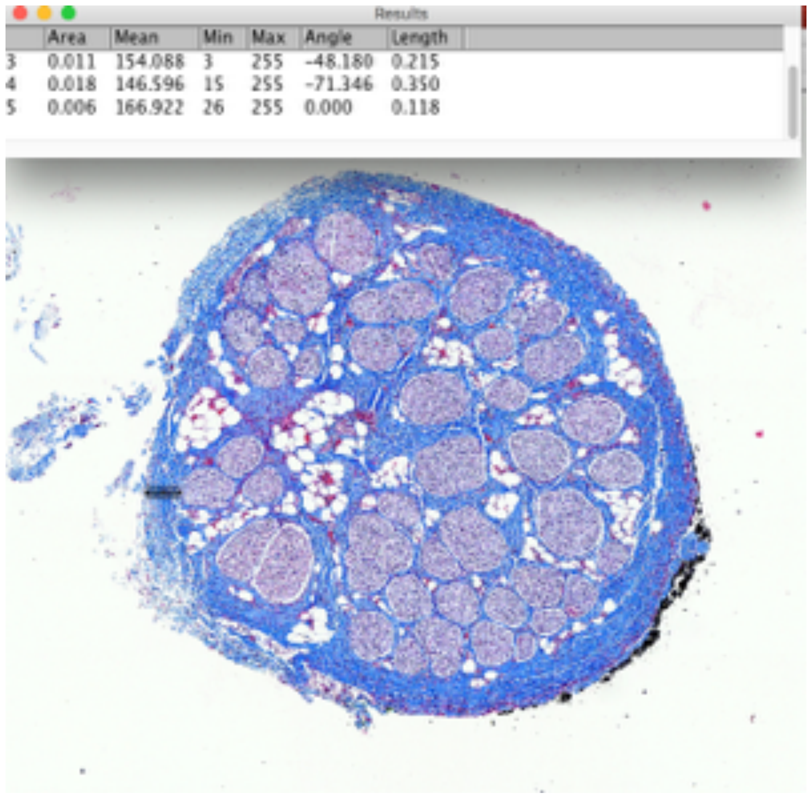

	Area	Mean	Min	Max	Angle	Length
3	0.011	154.088	3	255	-48.180	0.215
4	0.018	146.596	15	255	-71.346	0.350
5	0.006	166.922	26	255	0.000	0.118

largest fascicle_widest and narrowest diameters

Fascicle depth

RL widest and narrowest diameter

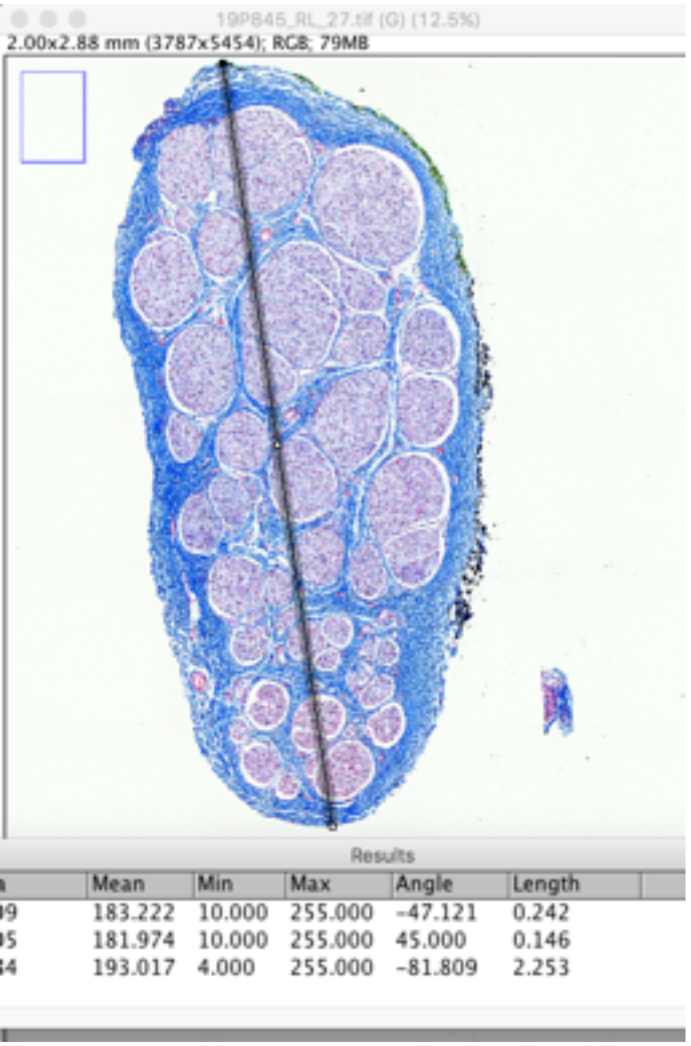

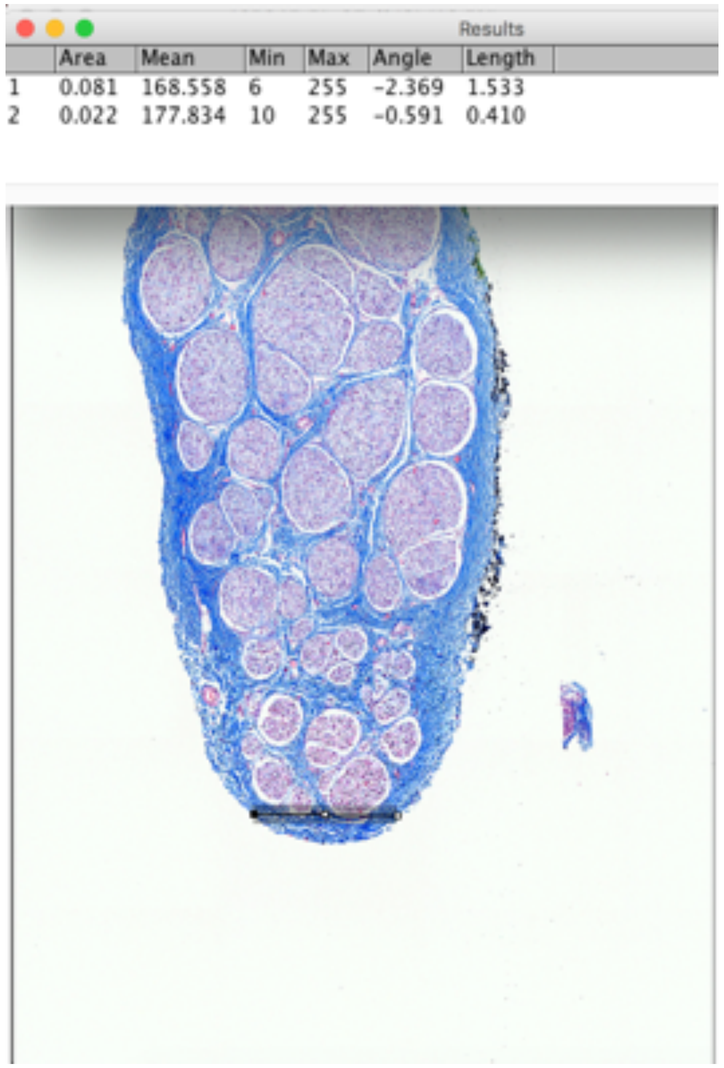

Fascicle count, 47

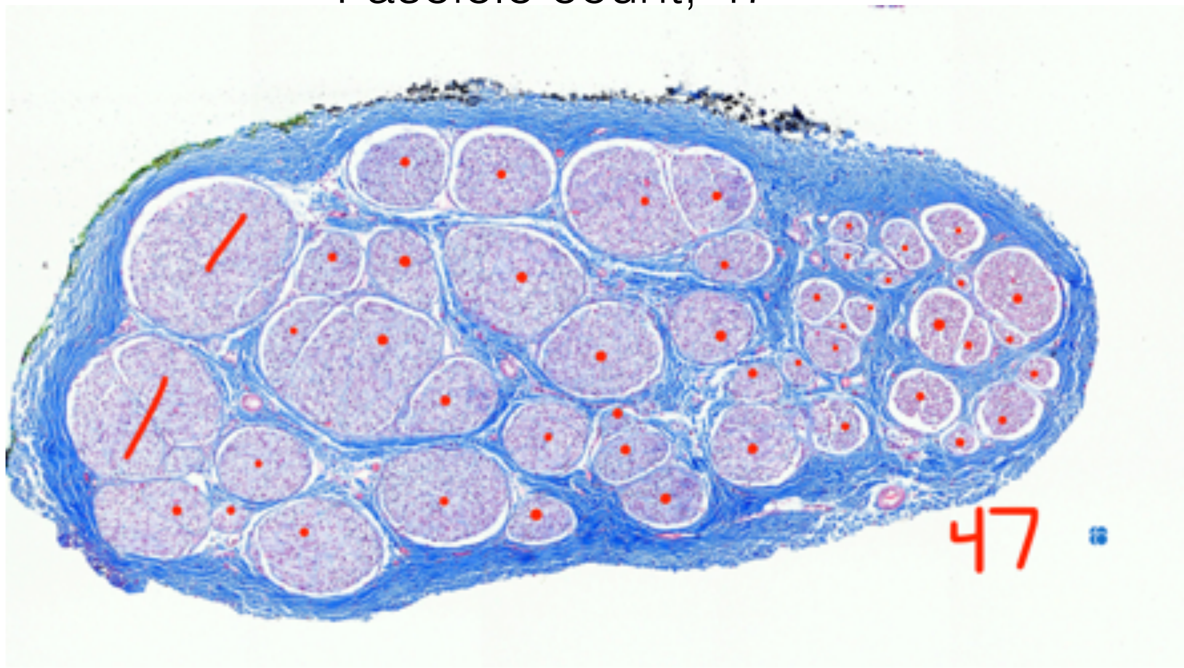

Largest fascicle_widest and narrowest diameter

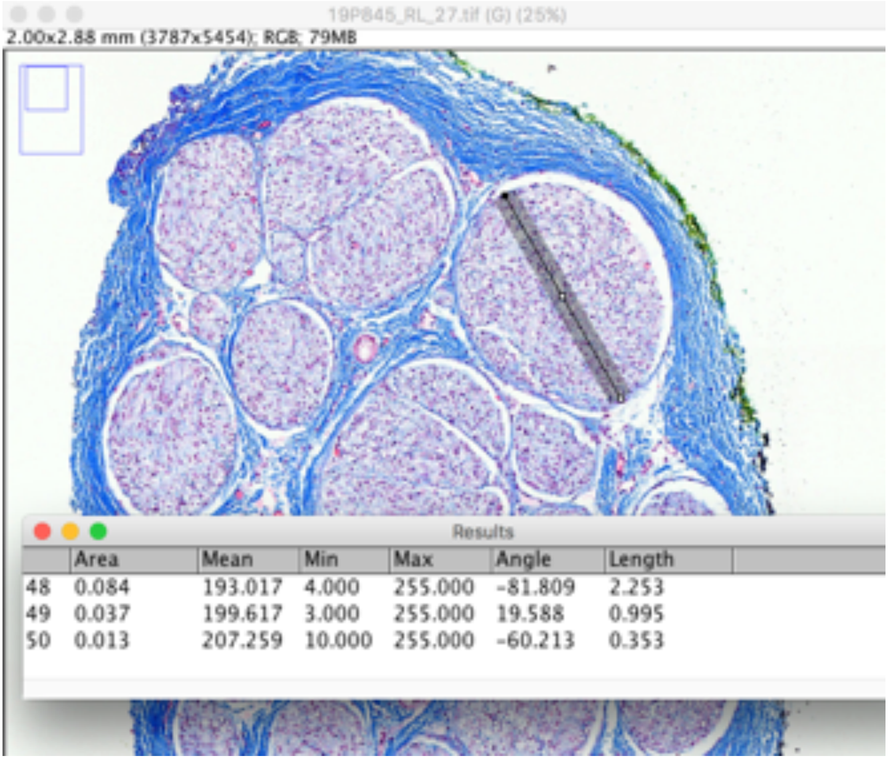

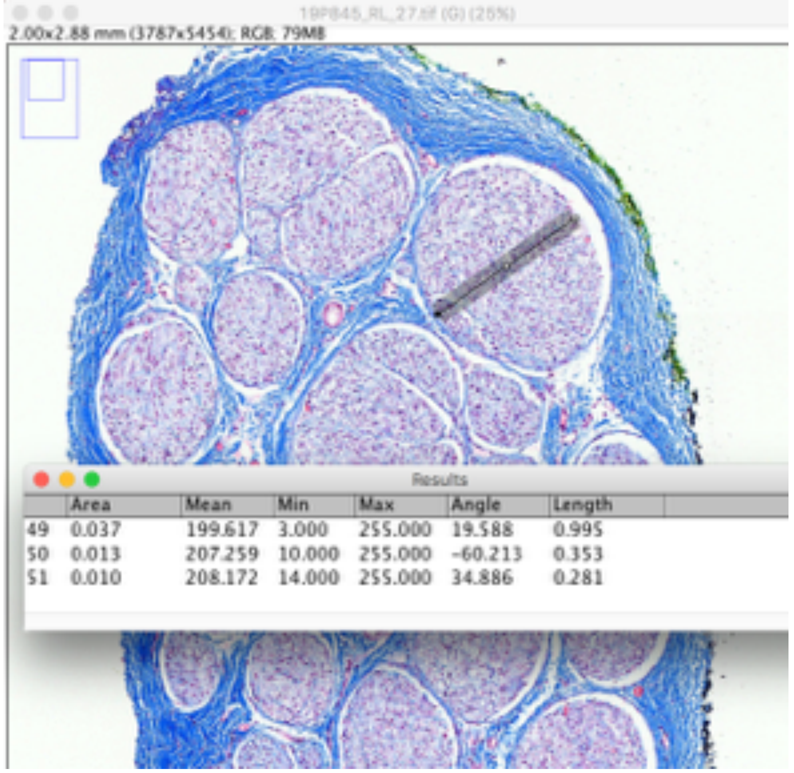

Subject 11_P853

Mid-VN

P853_VNb_4

Widest and narrowest diameter

Results

	Area	Mean	Min	Max	Angle	Length
1	0.107	185.115	13	255	58.499	2.898

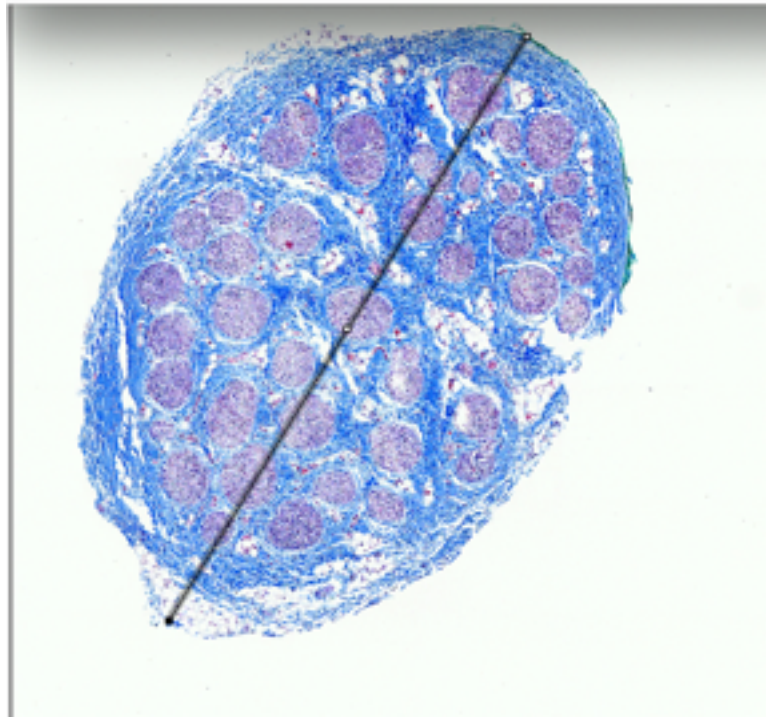

Results

	Area	Mean	Min	Max	Angle	Length
1	0.049	172.943	3	255	-35.446	0.918

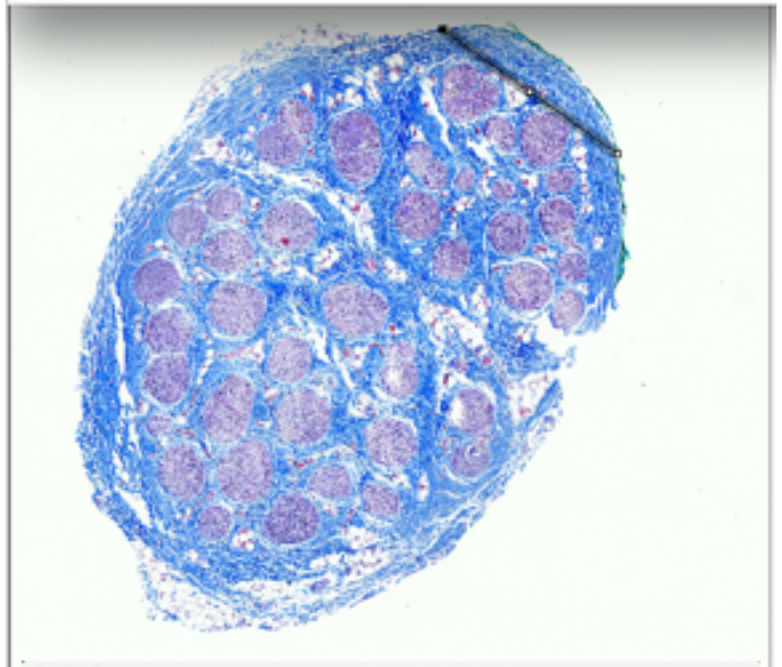

19P853_VNb_4.tif (G) (50%)

Results

	Area	Mean	Min	Max	Angle	Length
1	0.049	172.943	3	255	-35.446	0.918
2	0.003	193.766	32	255	-15.662	0.117

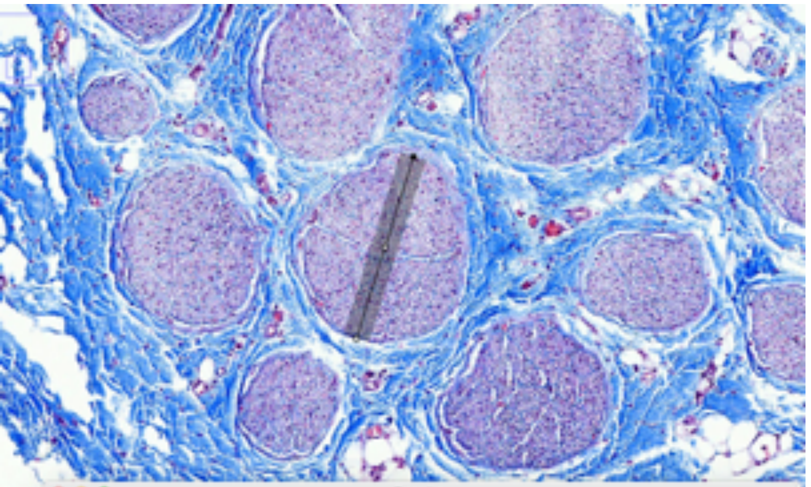

Results

	Area	Mean	Min	Max	Angle	Length
2	0.087	190.764	14	255	-73.196	2.323
3	0.075	187.509	10	255	-34.240	2.016
4	0.010	190.975	22	255	-107.457	0.264

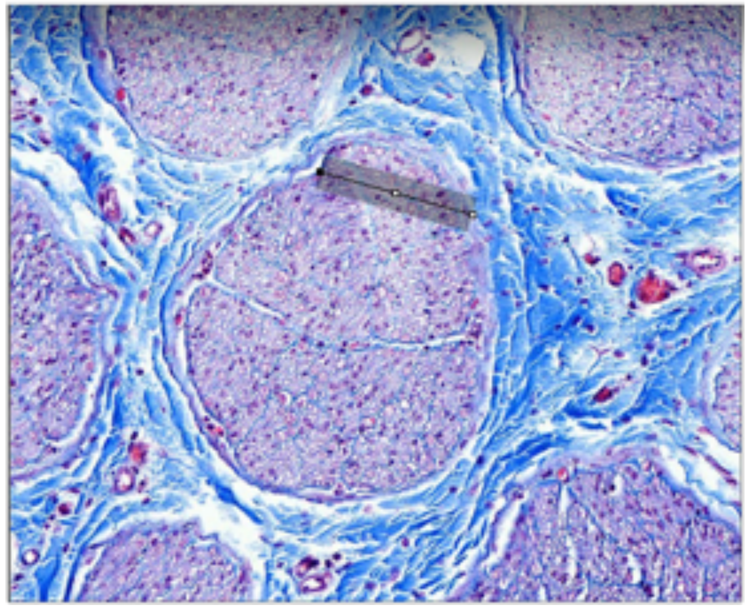

Largest fascicle_Widest and narrowest diameter

Fascicle count, 49

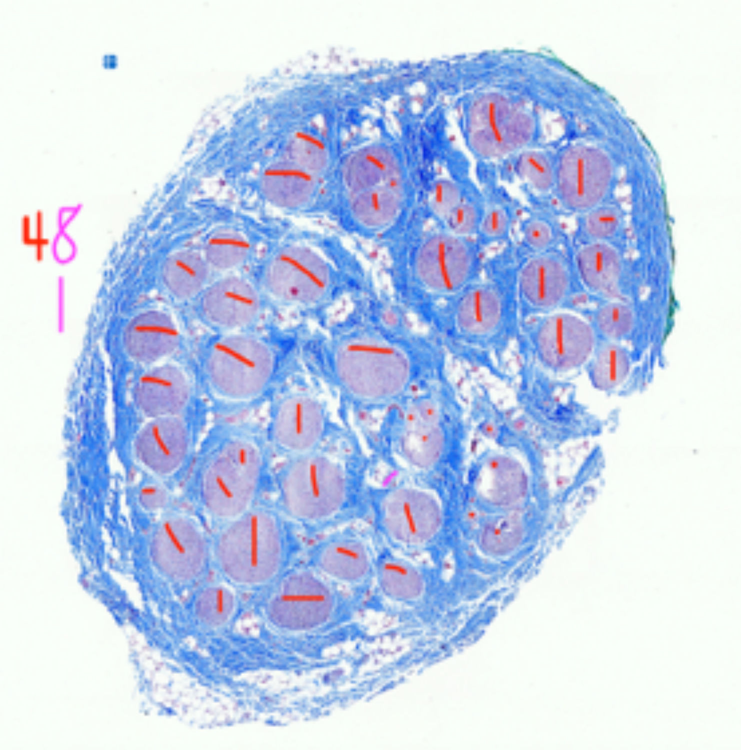

Results

	Area	Mean	Min	Max	Angle	Length
4	0.018	146.596	15	255	-71.346	0.350
5	0.006	166.922	26	255	0.000	0.118
6	0.006	185.318	16	255	-36.048	0.110

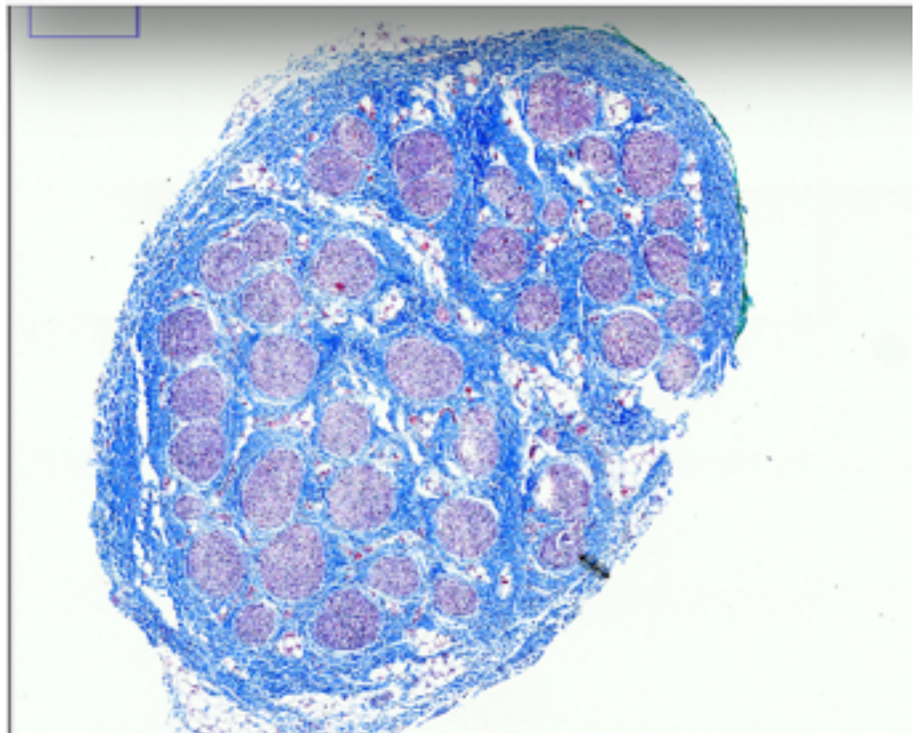

Fascicle depth

Subject 1_P786

Pre-nodose

Widest and narrowest diameter

19P786_LV2b_cranial nodose

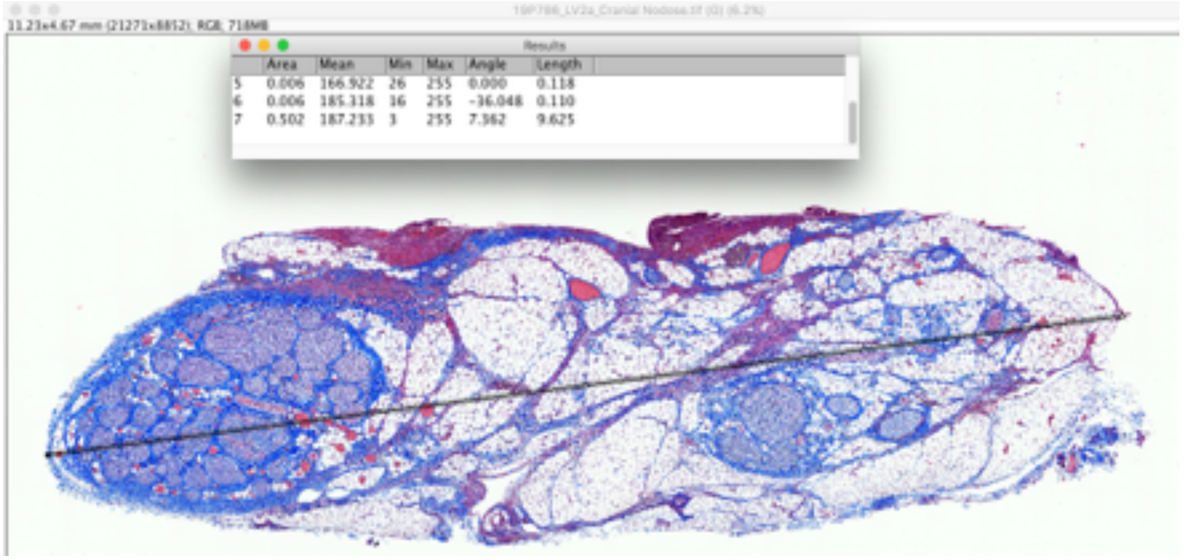

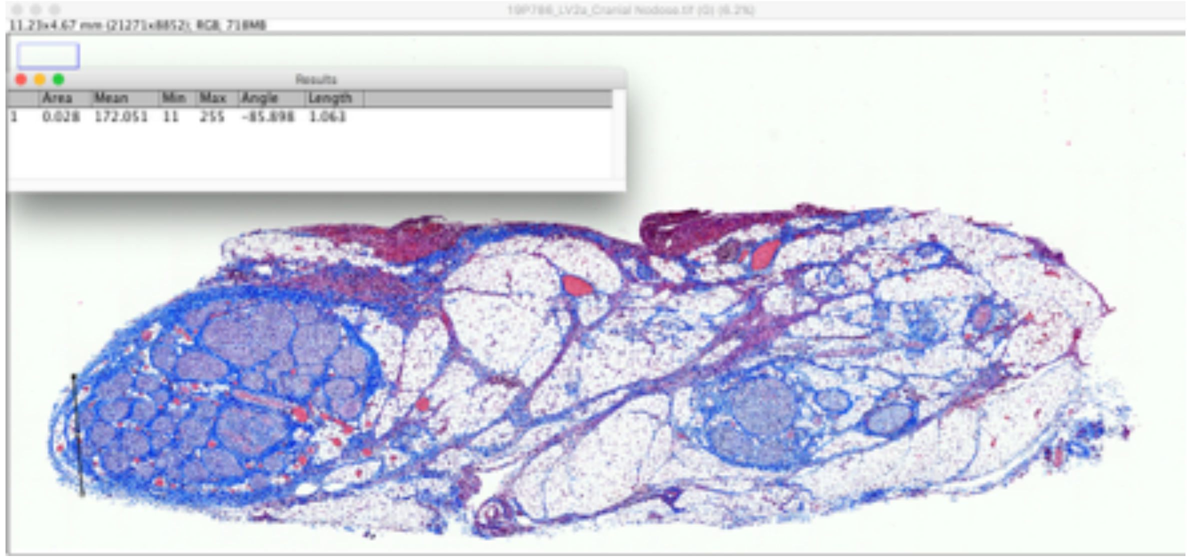

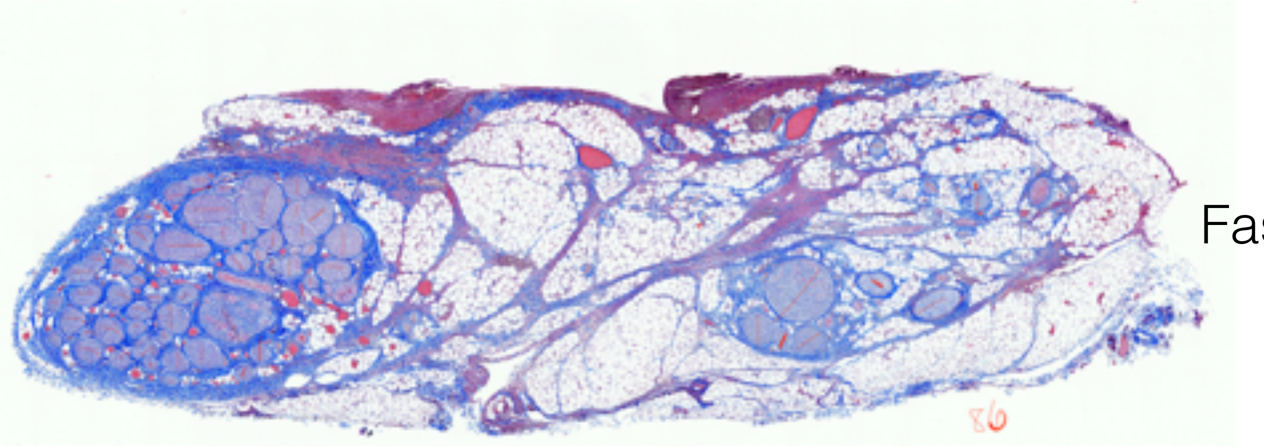

Fascicle count, 86

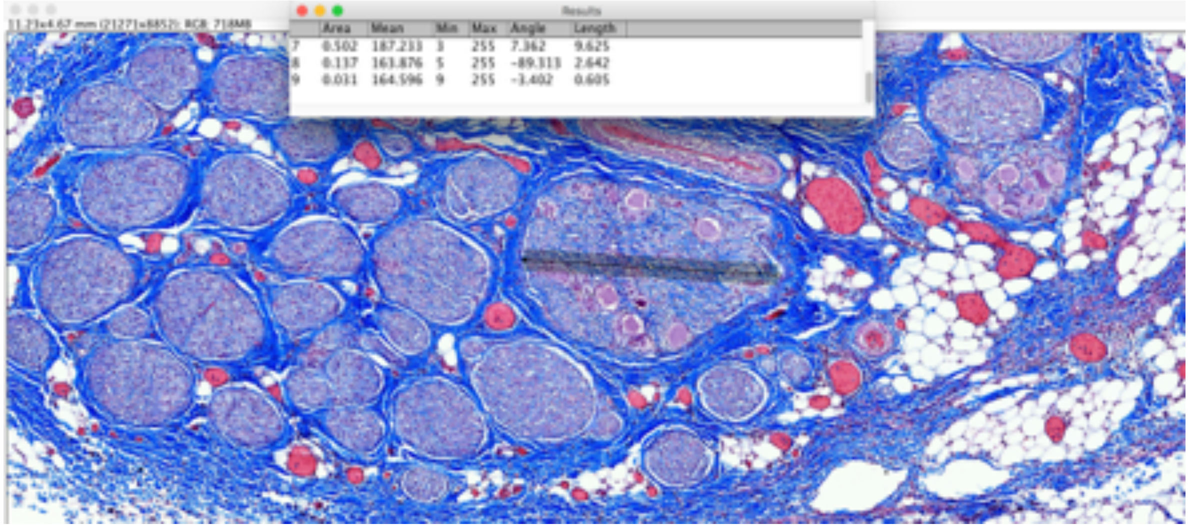

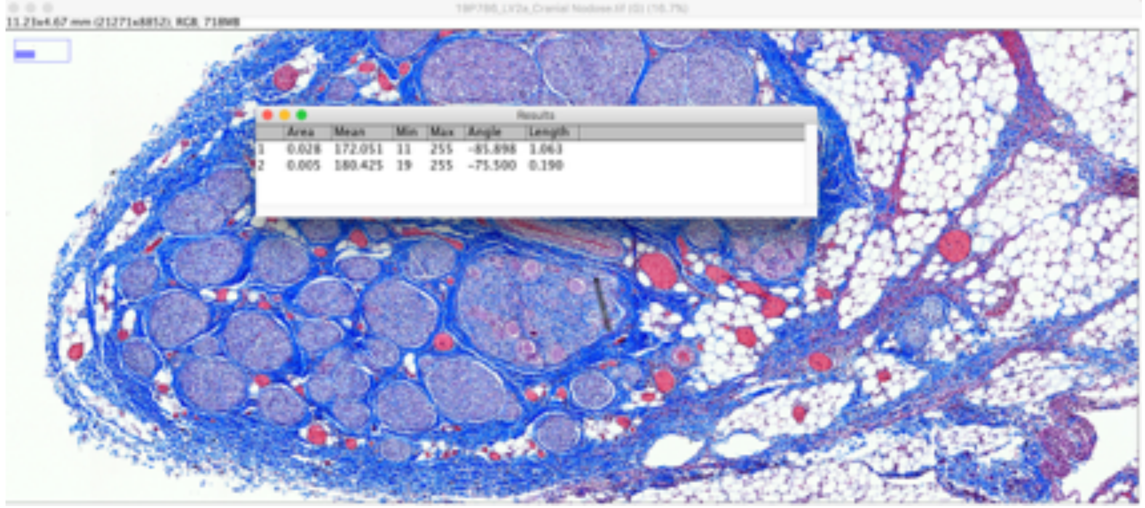

Largest fascicle_widest and narrowest diameter

P786_LV1b_LivaNova

Mid-VN

	Area	Mean	Min	Max	Angle	Length
4	0.010	190.975	22	255	-107.457	0.264
5	0.008	186.969	11	255	-24.341	0.219
6	0.145	190.955	3	255	-103.529	3.936

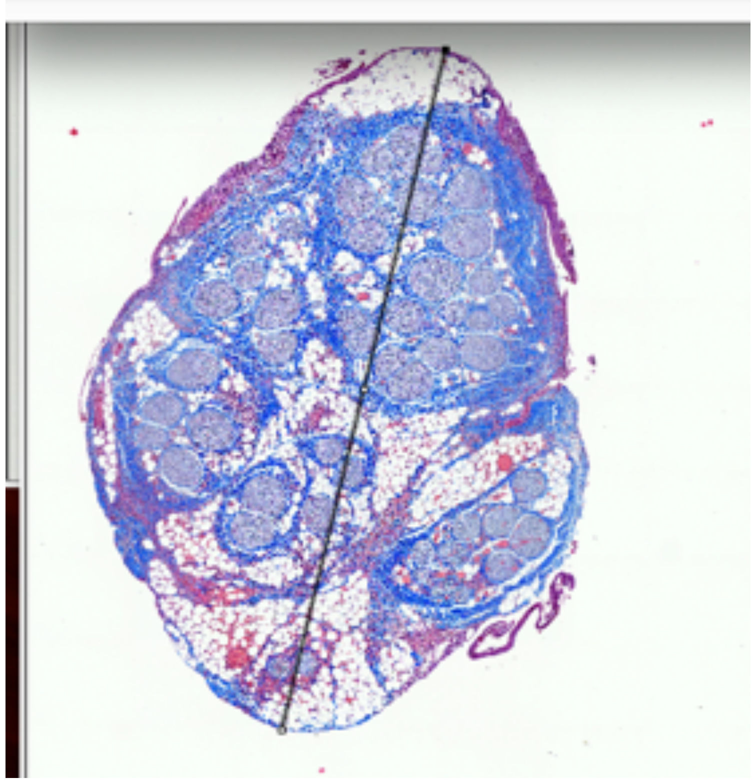

Widest and narrowest diameter

	Area	Mean	Min	Max	Angle	Length
1	0.028	172.051	11	255	-85.898	1.063
2	0.005	180.425	19	255	-75.500	0.190
3	0.034	165.655	9	255	-9.273	1.258

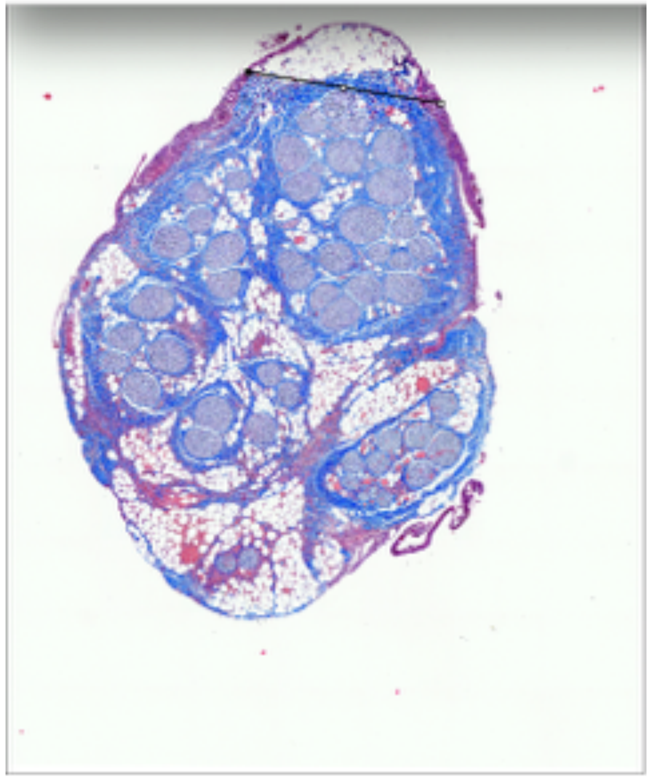

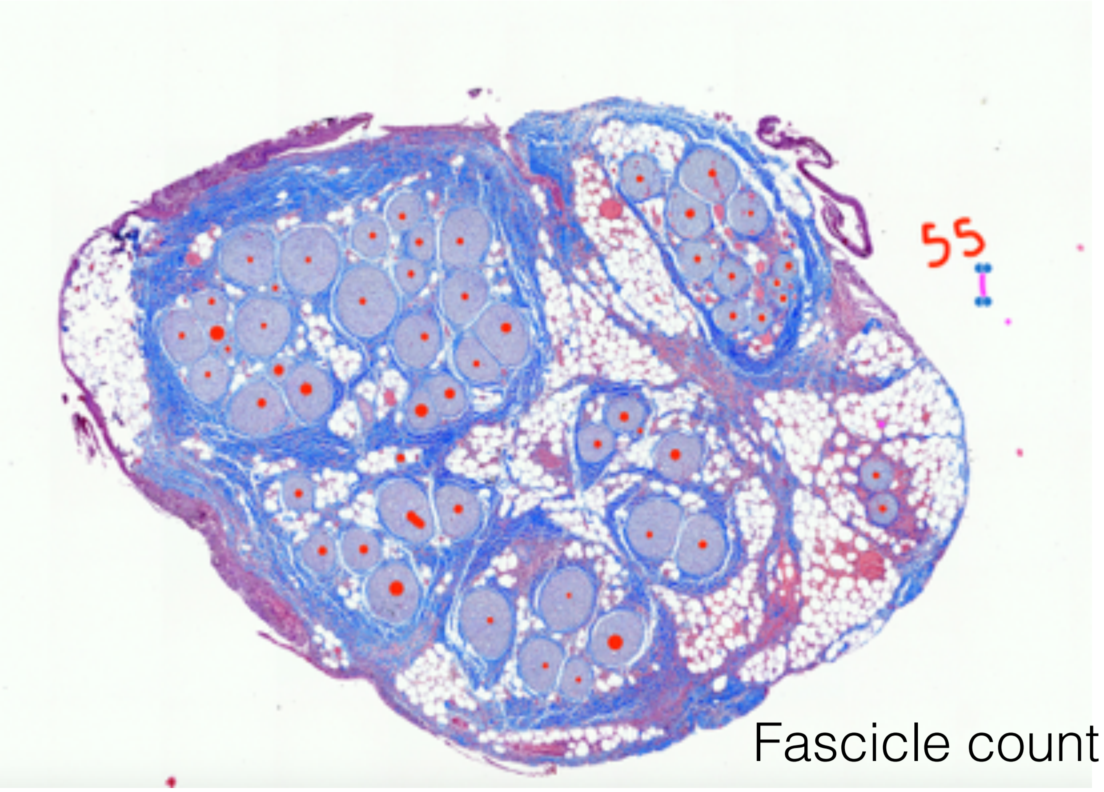

Fascicle count, 55

Largest fascicle_widest and narrowest diameter

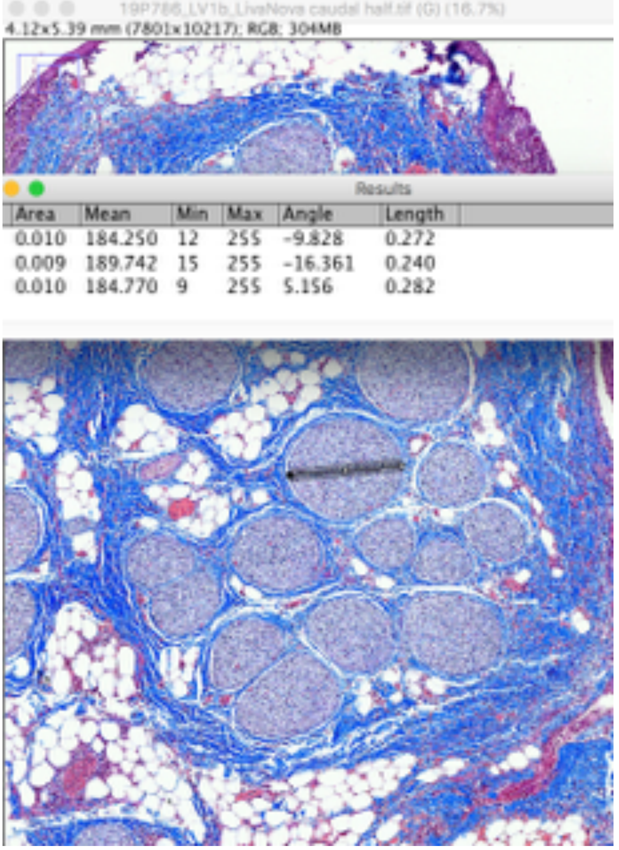

Area	Mean	Min	Max	Angle	Length
0.010	184.250	12	255	-9.828	0.272
0.009	189.742	15	255	-16.361	0.240
0.010	184.770	9	255	5.156	0.282

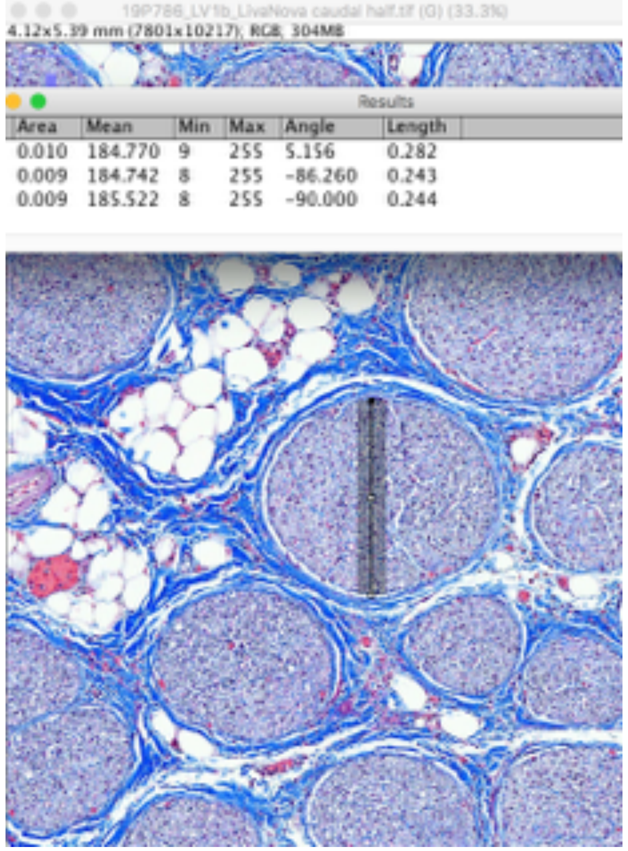

Area	Mean	Min	Max	Angle	Length
0.010	184.770	9	255	5.156	0.282
0.009	184.742	8	255	-86.260	0.243
0.009	185.522	8	255	-90.000	0.244

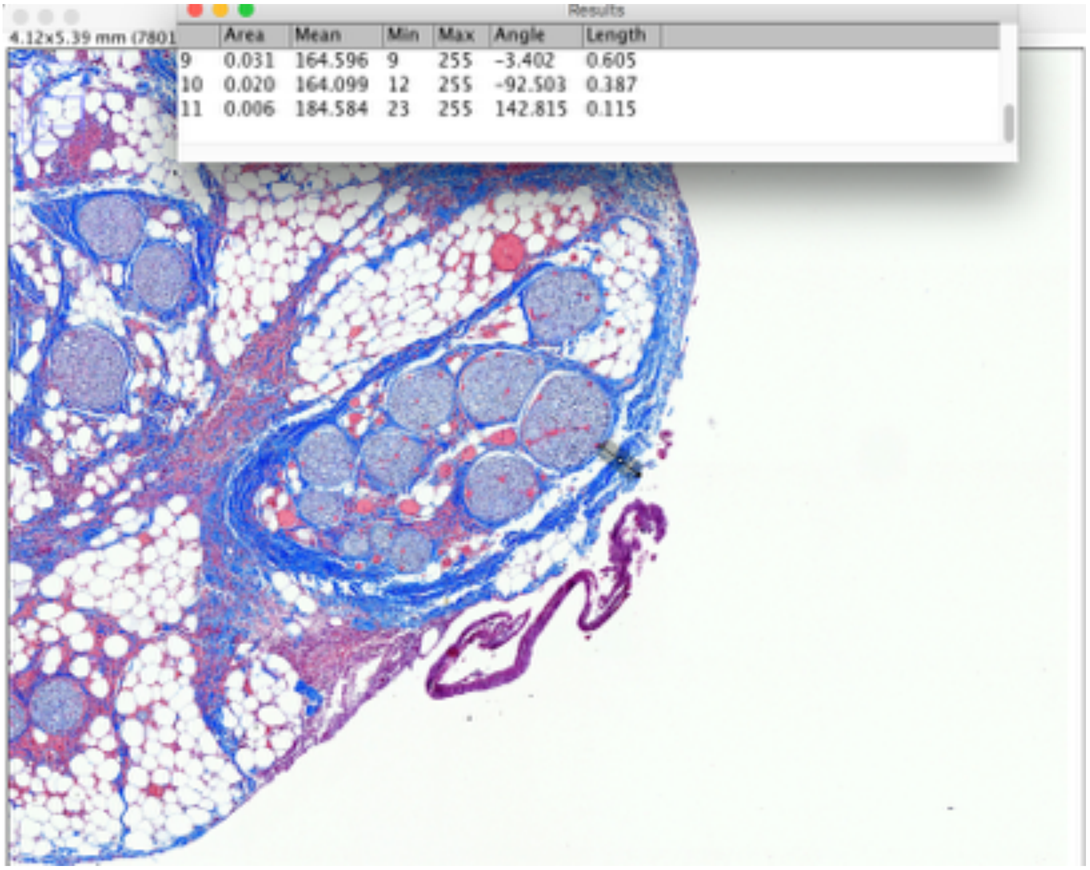

	Area	Mean	Min	Max	Angle	Length
9	0.031	164.596	9	255	-3.402	0.605
10	0.020	164.099	12	255	-92.503	0.387
11	0.006	184.584	23	255	142.815	0.115

Fascicle depth

Subject 3_P824

Nodose Widest and narrowest diameter P824_nodose_a_2, nodose

Results

Area	Mean	Min	Max	Angle	Length
0.009	184.742	8	255	-86.260	0.243
0.009	185.522	8	255	-90.000	0.244
0.210	173.693	3	255	-66.517	5.692

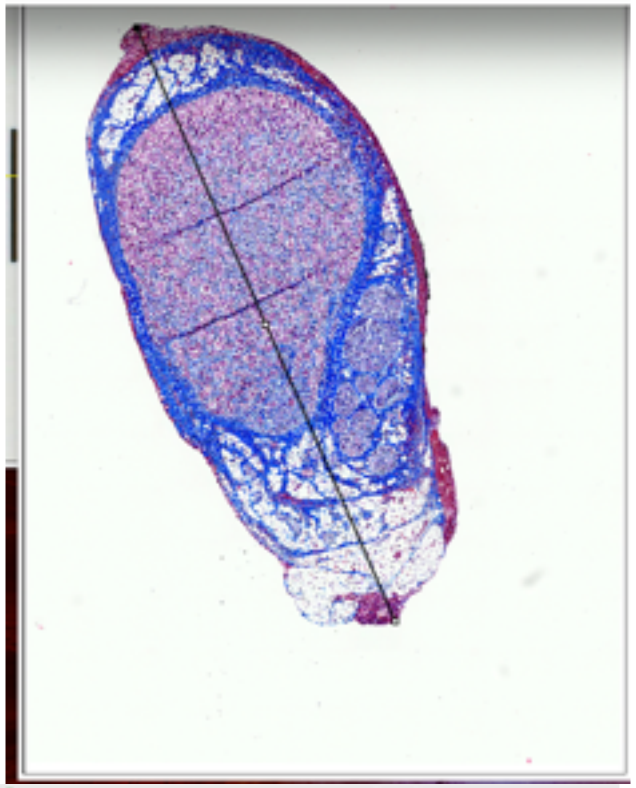

Results

	Area	Mean	Min	Max	Angle	Length
1	0.052	139.610	5	255	21.444	2.010

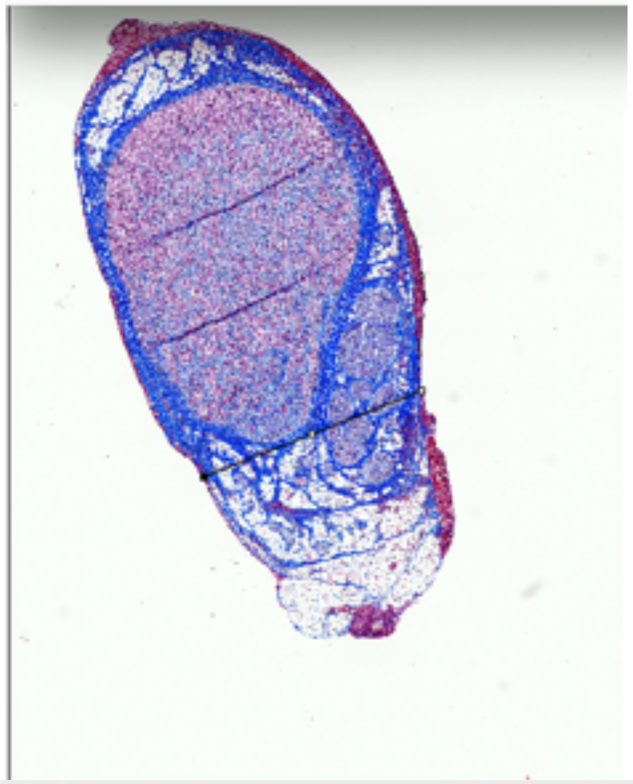

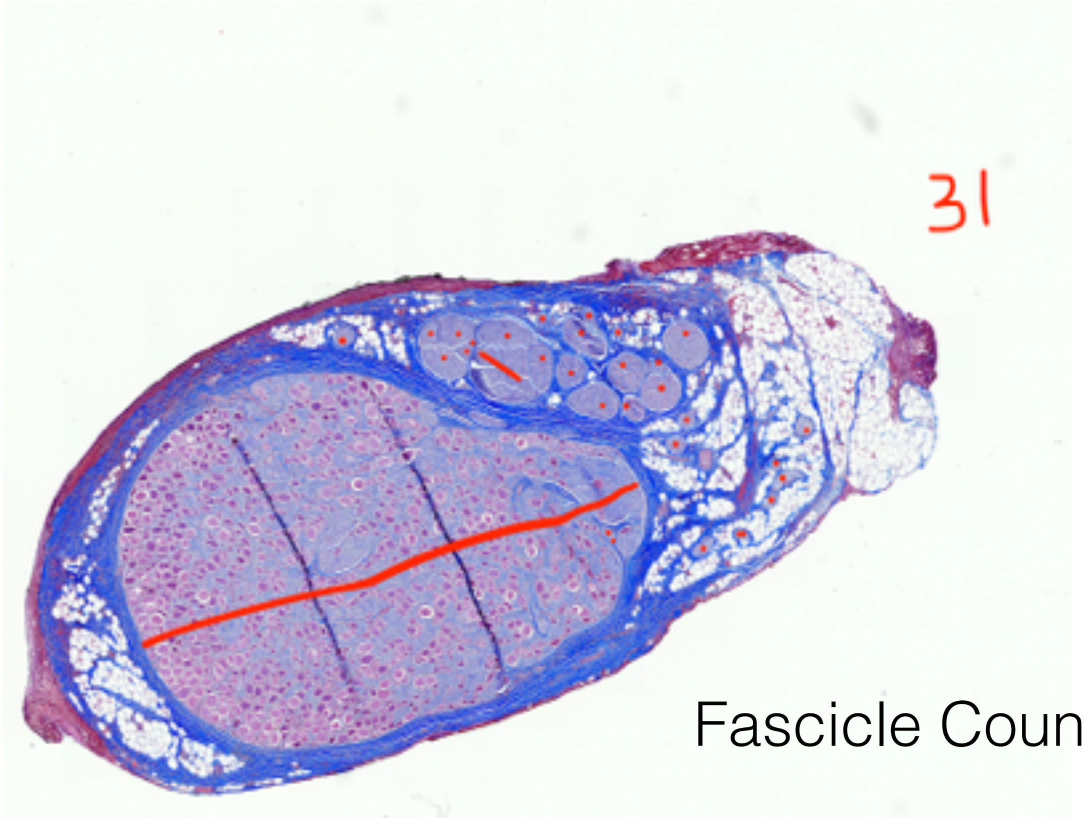

Fascicle Count, 31

Results

rea	Mean	Min	Max	Angle	Length
.210	173.693	3	255	-66.517	5.692
.097	163.632	5	255	25.320	2.607
.112	167.214	6	255	-74.766	3.014

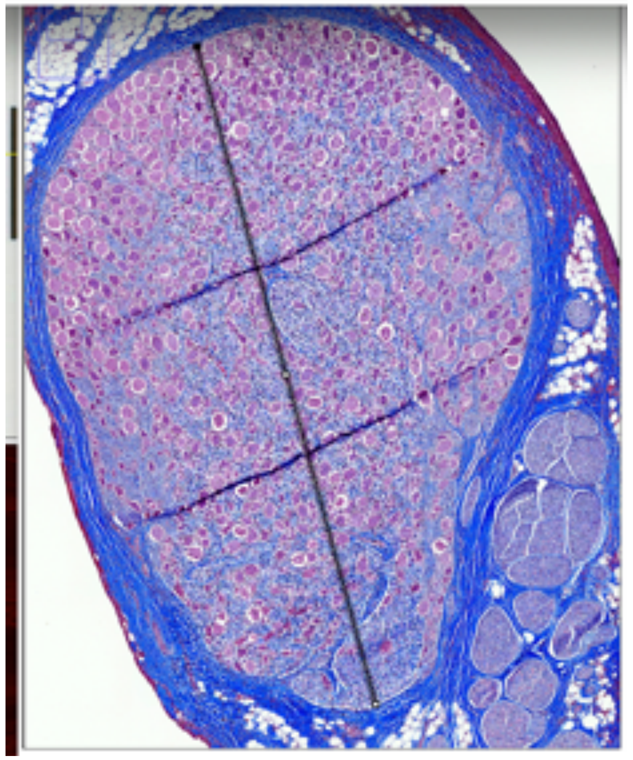

Results

	Area	Mean	Min	Max	Angle	Length
1	0.052	139.610	5	255	21.444	2.010
2	0.033	168.442	11	255	24.969	1.221
3	0.017	164.908	12	255	32.530	0.636

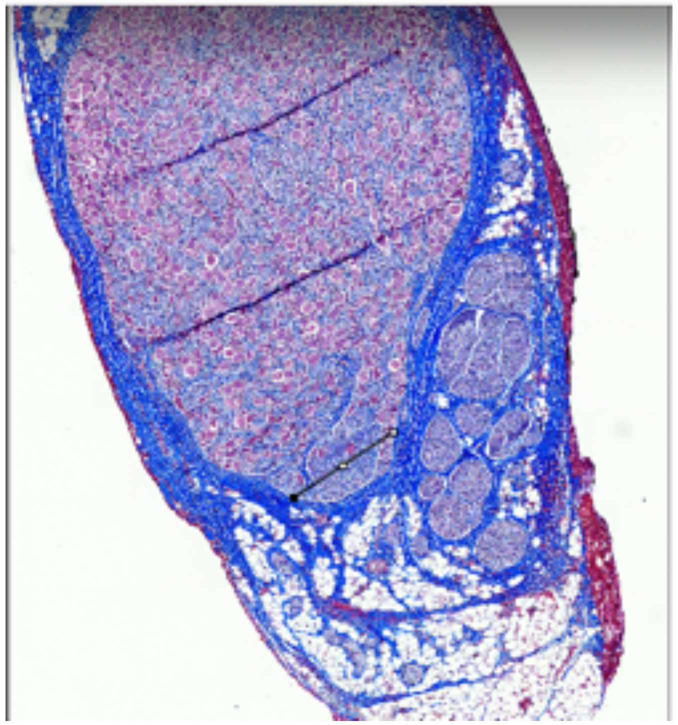

Largest fascicle_widest and narrowest diameters

Subject 4_P833

Nodose

Widest and narrowest diameter

P833_RV1_18, nodose

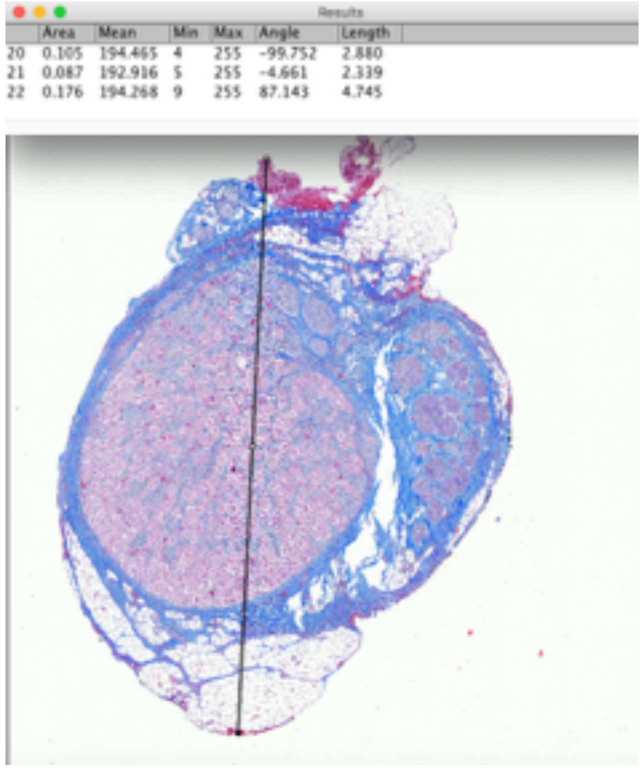

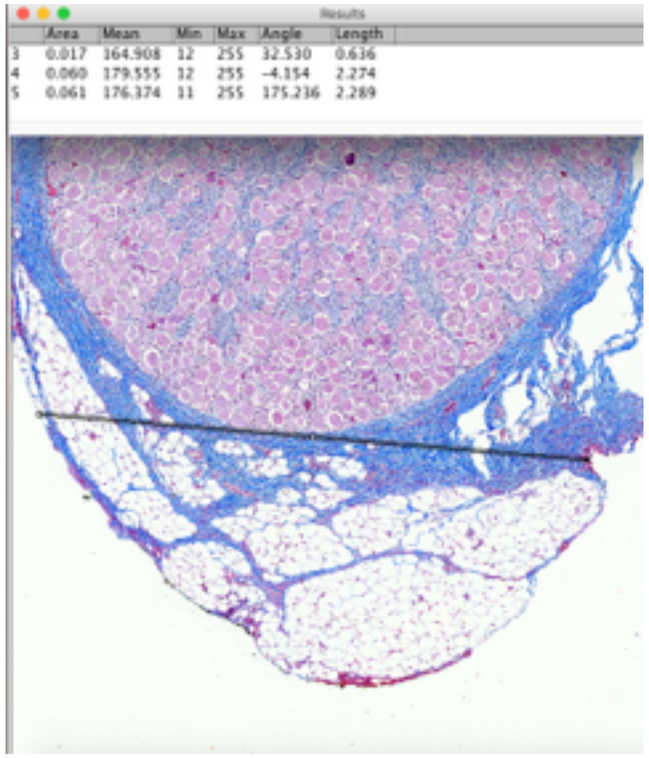

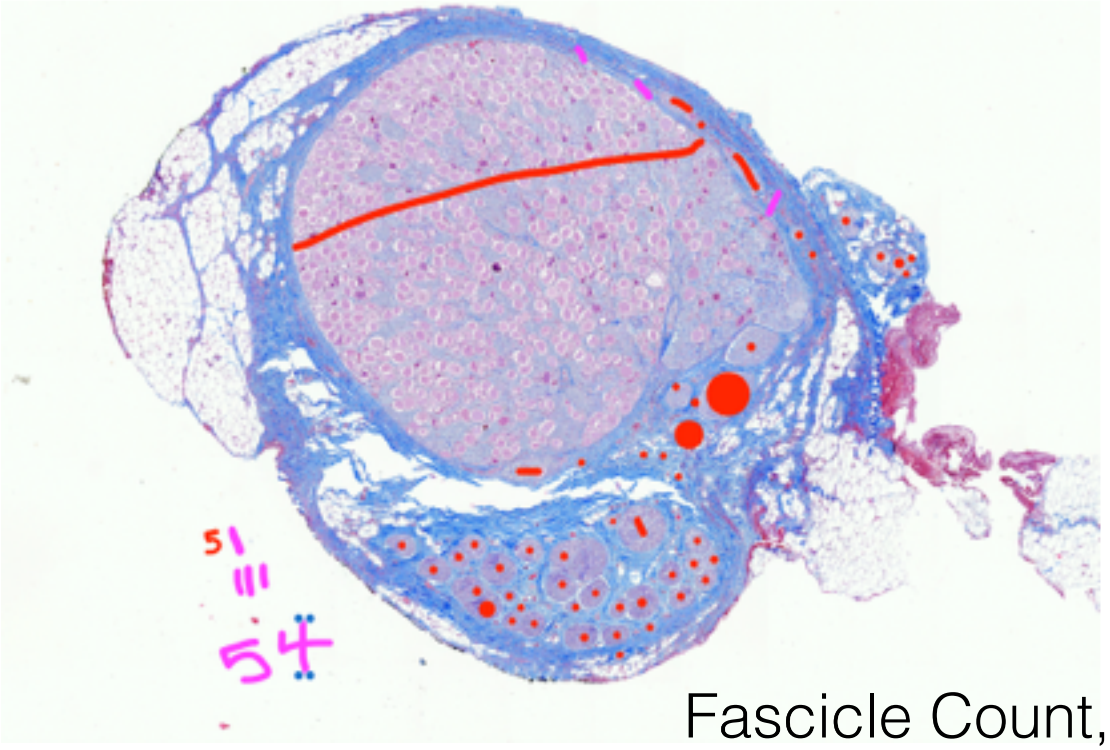

Fascicle Count, 54

Largest fascicle_widest and narrowest diameters

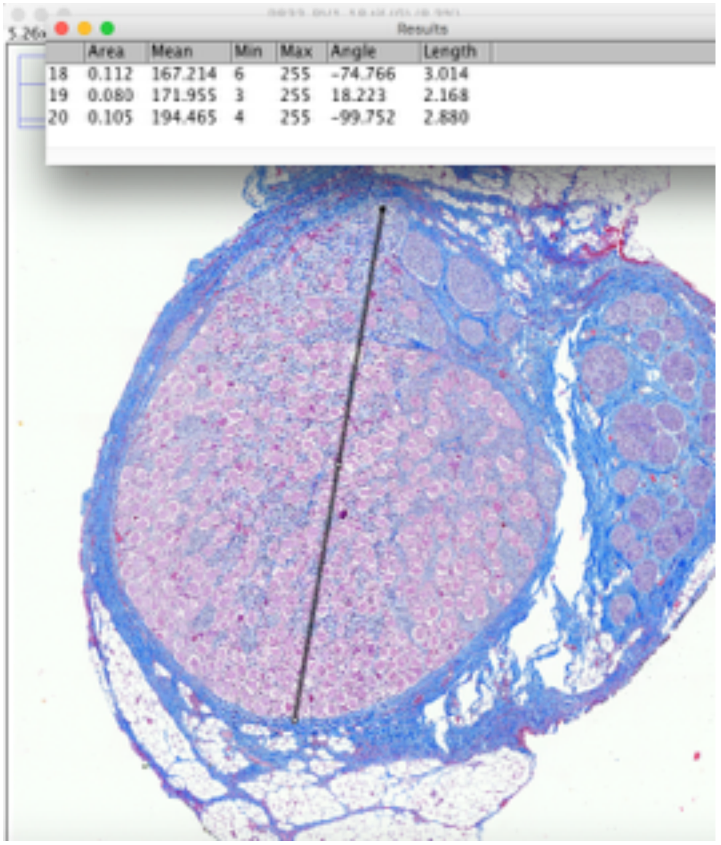

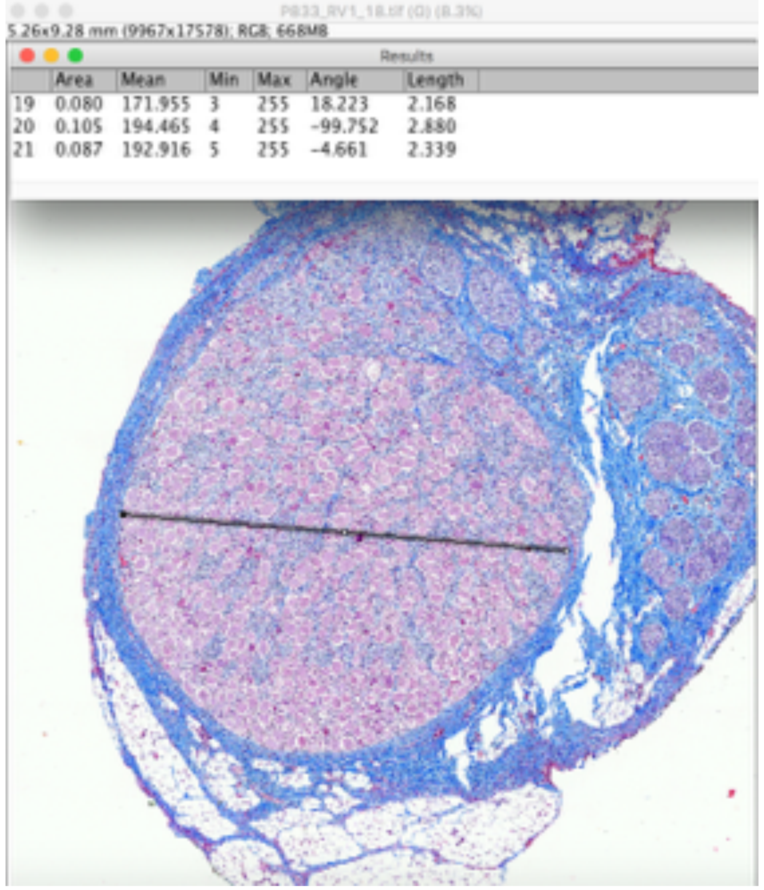

Mid-VN

P833_RV2b_2

Widest and narrowest diameters

Results

	Area	Mean	Min	Max	Angle	Length
22	0.176	194.268	9	255	87.143	4.745
23	0.142	188.352	7	255	9.609	3.796
24	0.110	181.322	5	255	-113.790	2.984

3.15x3.43 mm (5965x6489); RGB; 148MB

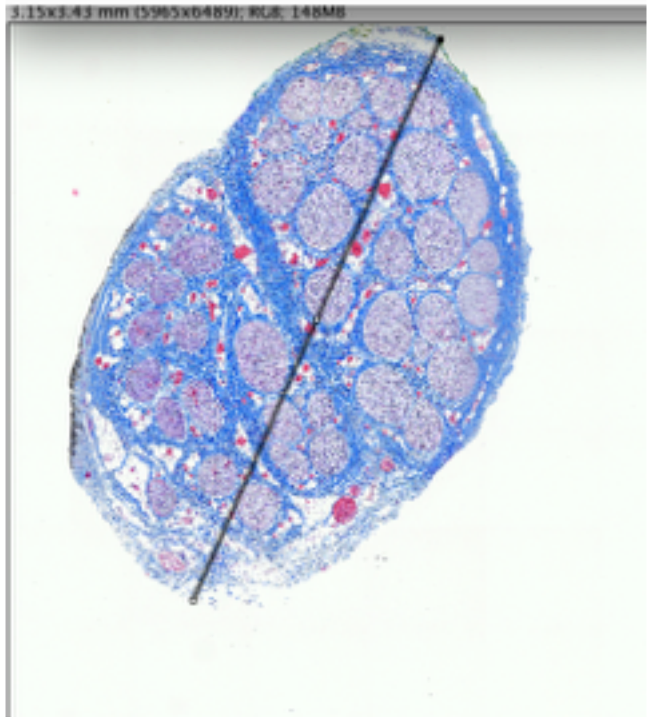

Results

	Area	Mean	Min	Max	Angle	Length
1	0.050	179.520	13	255	-57.894	1.907

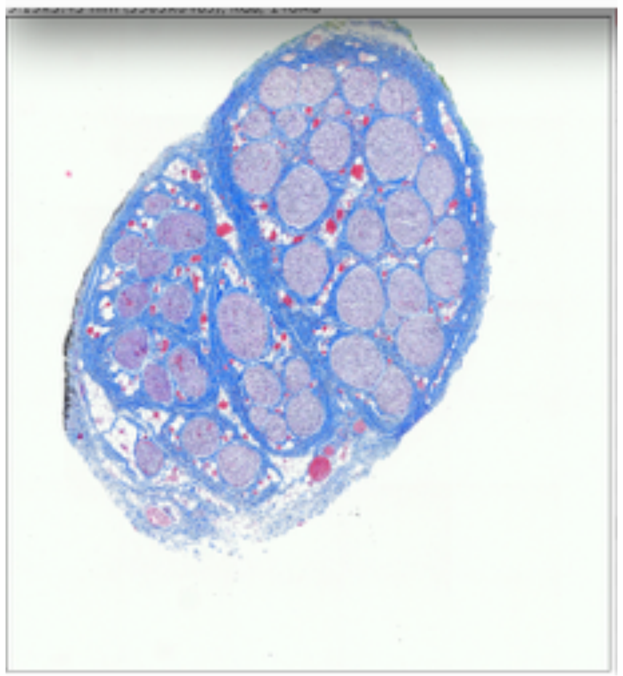

Fascicle Depth

P833_RV2b_2.tif (G) (33.3%)

x3.43 mm (5965x6489); RGB; 148MB

Results

	Area	Mean	Min	Max	Angle	Length
11	0.006	184.584	23	255	142.815	0.115
12	0.004	174.451	1	255	-24.349	0.079
13	0.004	170.578	1	255	-29.168	0.078

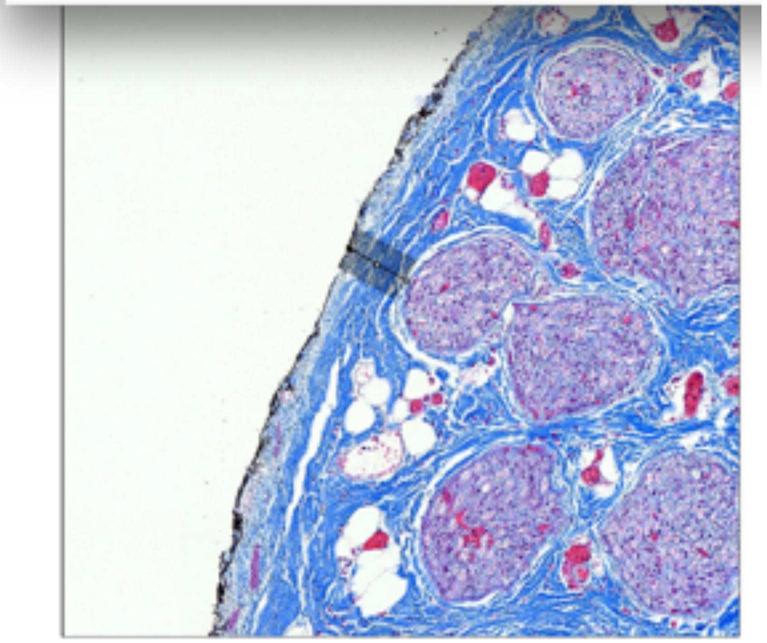

Largest Fascicle_Widest and narrowest diameters

P833_RV2b_2.tif (G) (12.5%)

3.15x3.43 mm (5965x6489); RGB; 148MB

Results

Area	Mean	Min	Max	Angle	Length
0.110	181.322	5	255	-113.790	2.984
0.072	179.130	4	255	-49.179	1.968
0.013	190.754	9	255	-69.085	0.355

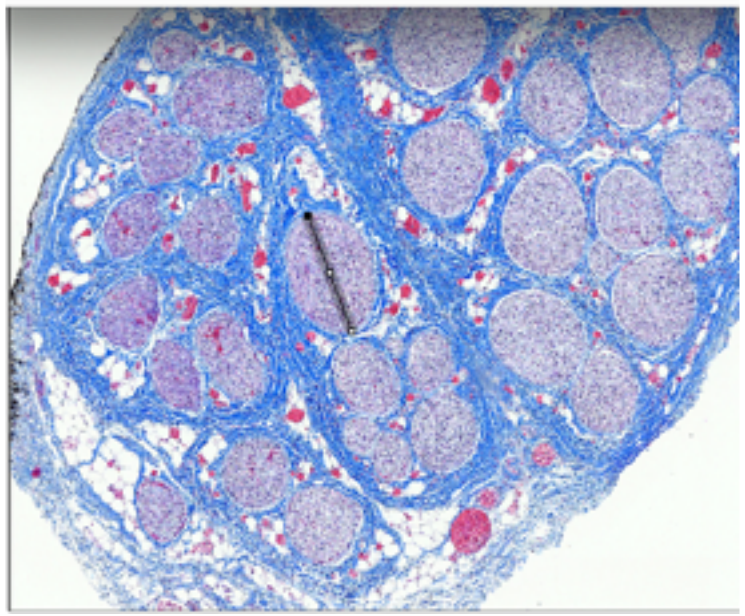

Results

Area	Mean	Min	Max	Angle	Length
0.072	179.130	4	255	-49.179	1.968
0.013	190.754	9	255	-69.085	0.355
0.009	190.395	7	255	27.229	0.245

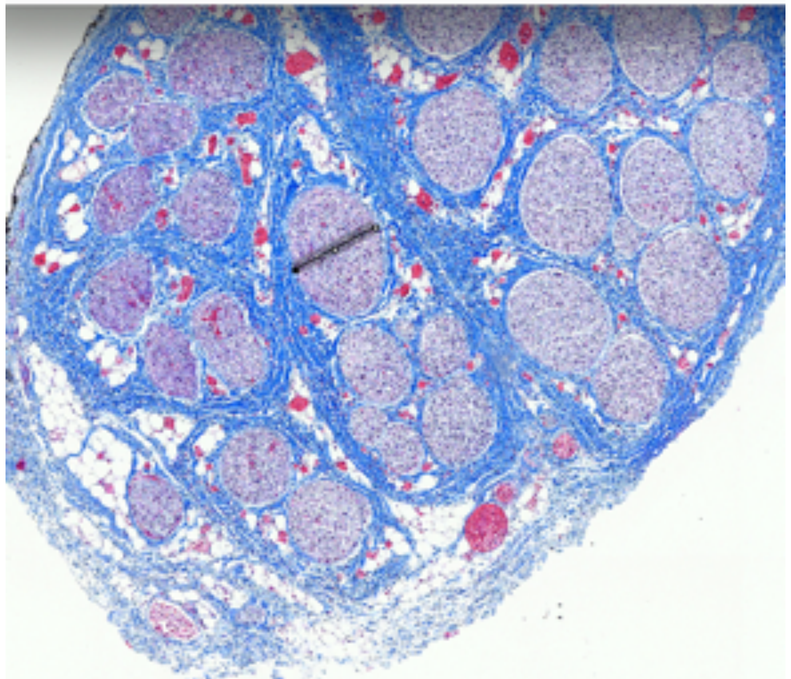

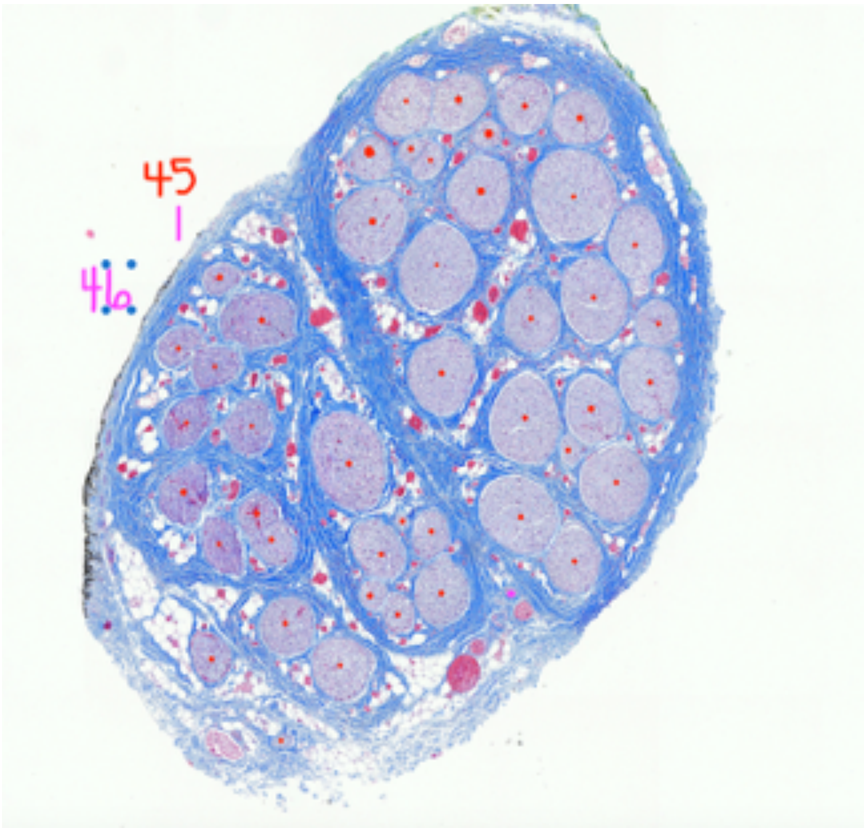

Fascicle count, 46

RL

Widest and narrowest diameters

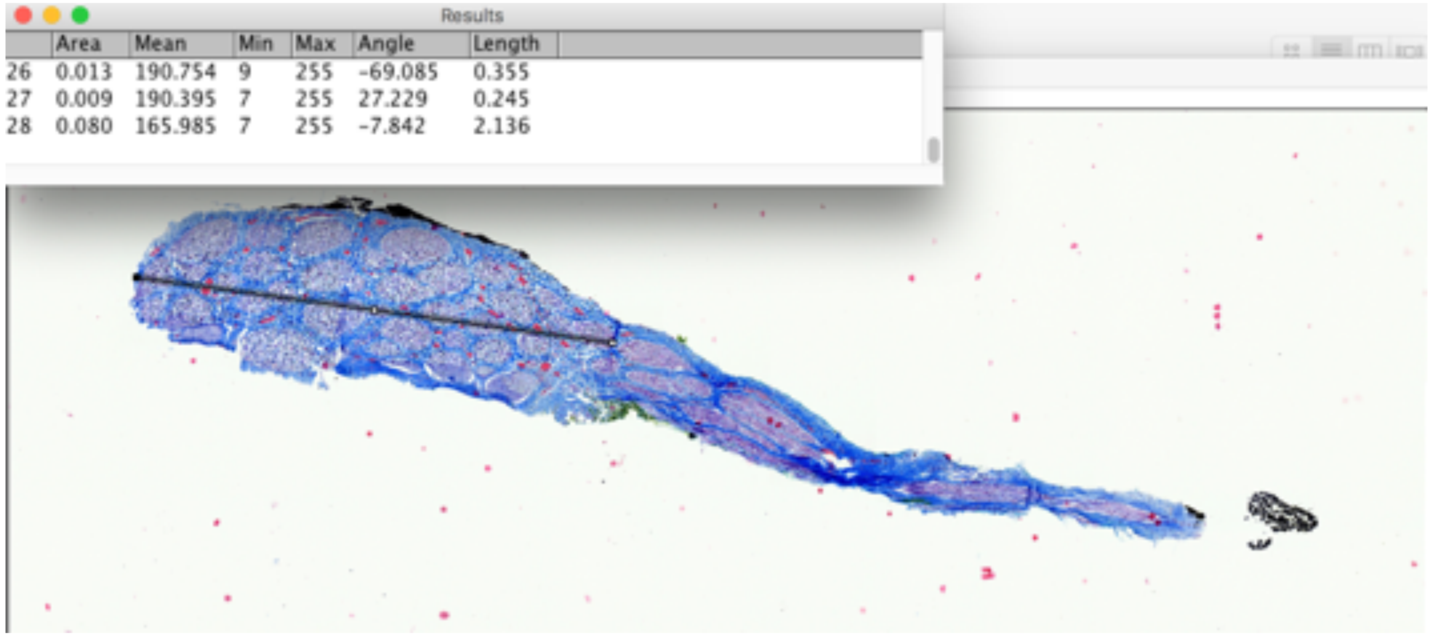

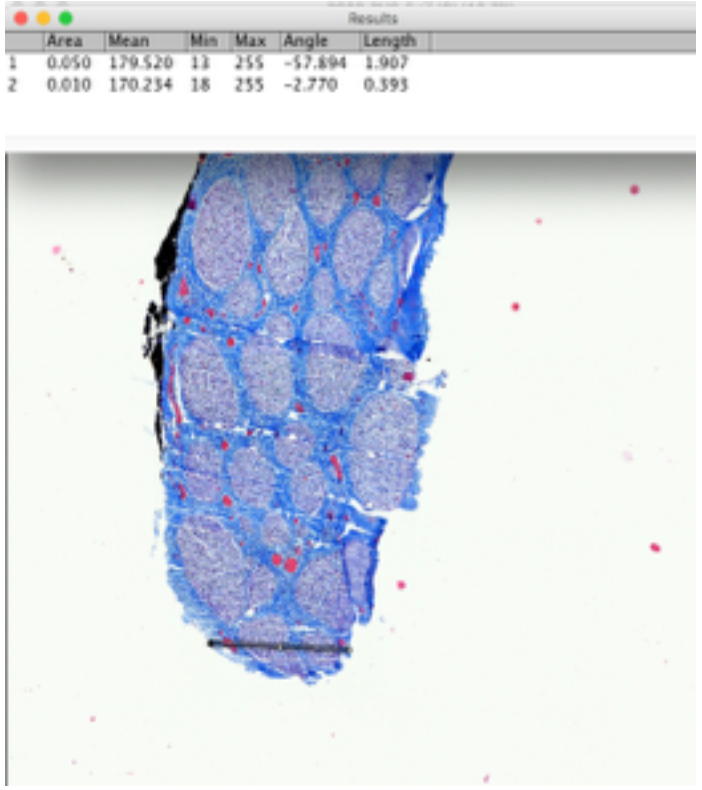

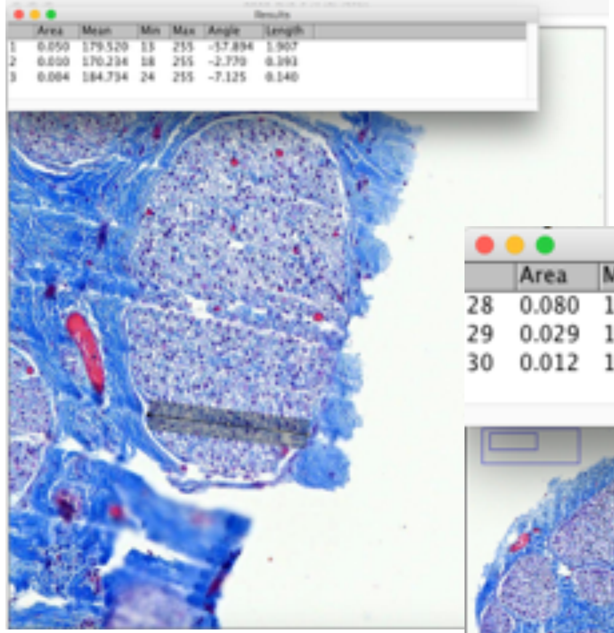

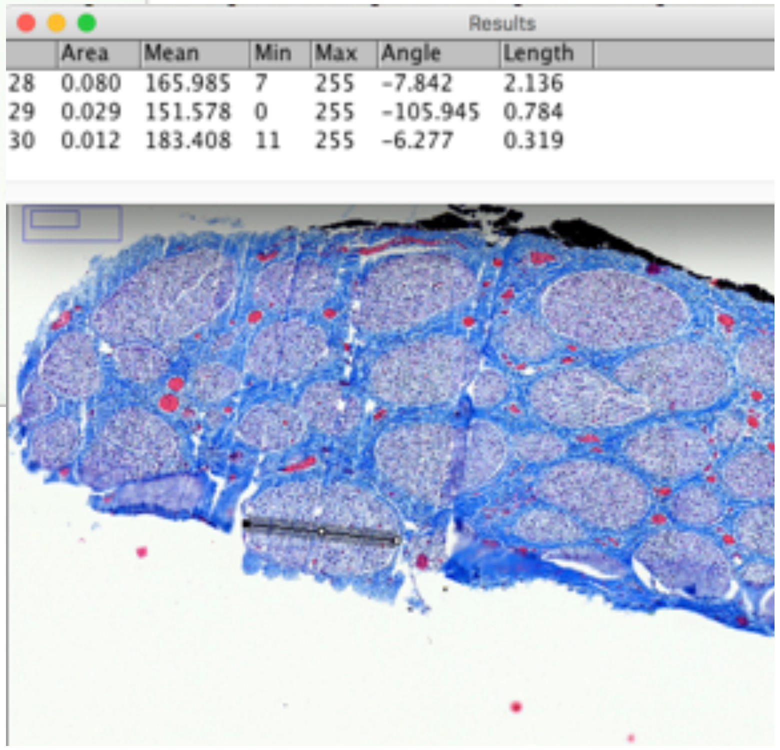

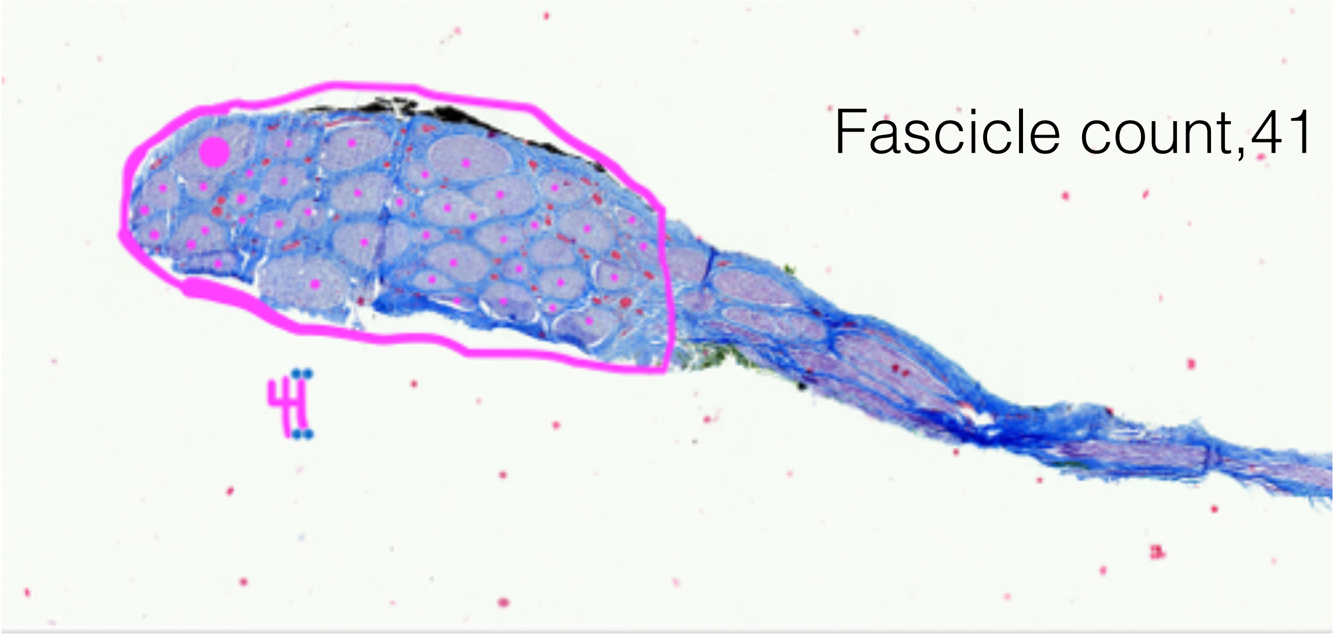

Fascicle count,41

Largest fascicle_widest and narrowest diameter

Subject 5_P836

Nodose

Largest fascicle_widest and narrowest diameter

P836_RV3_41

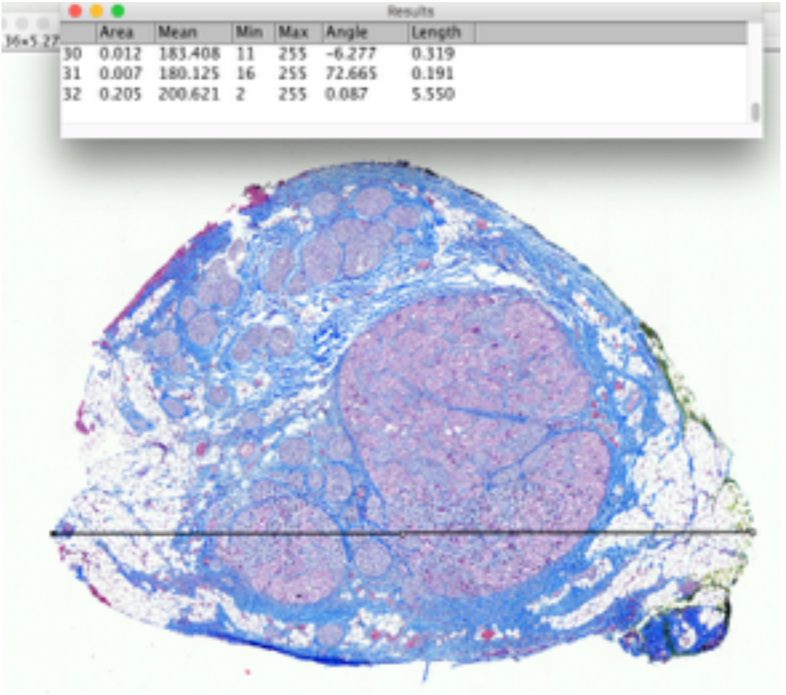

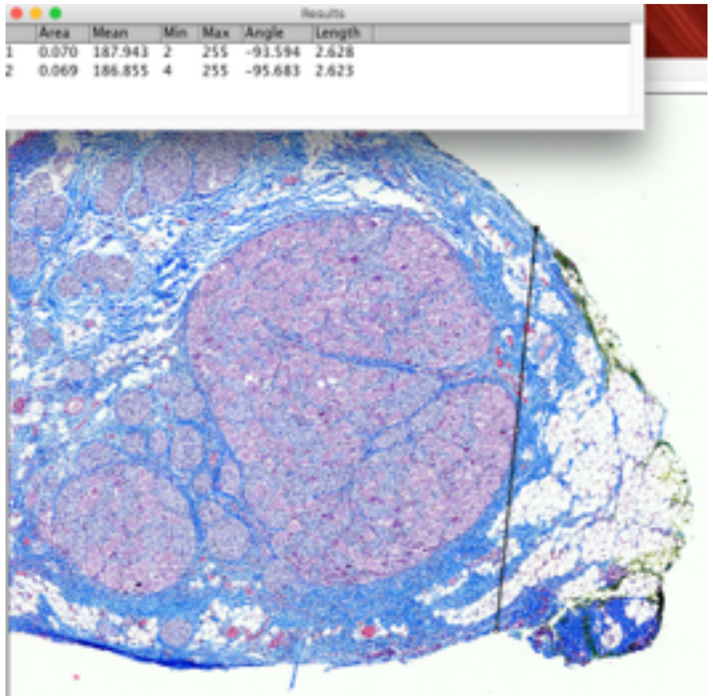

Fascicle Count, 58

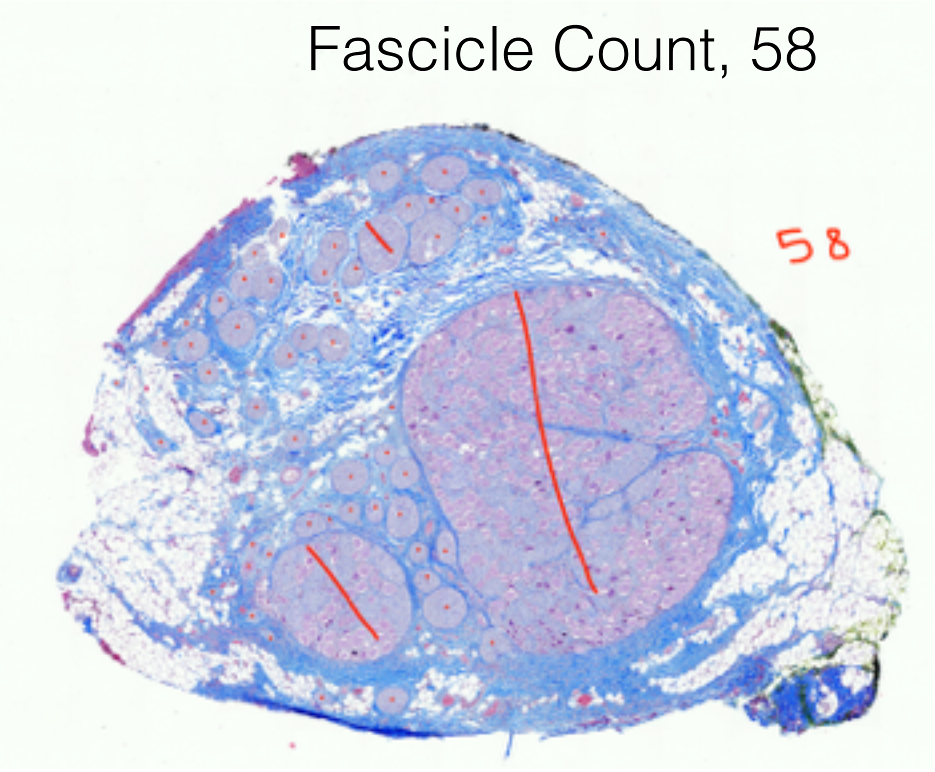

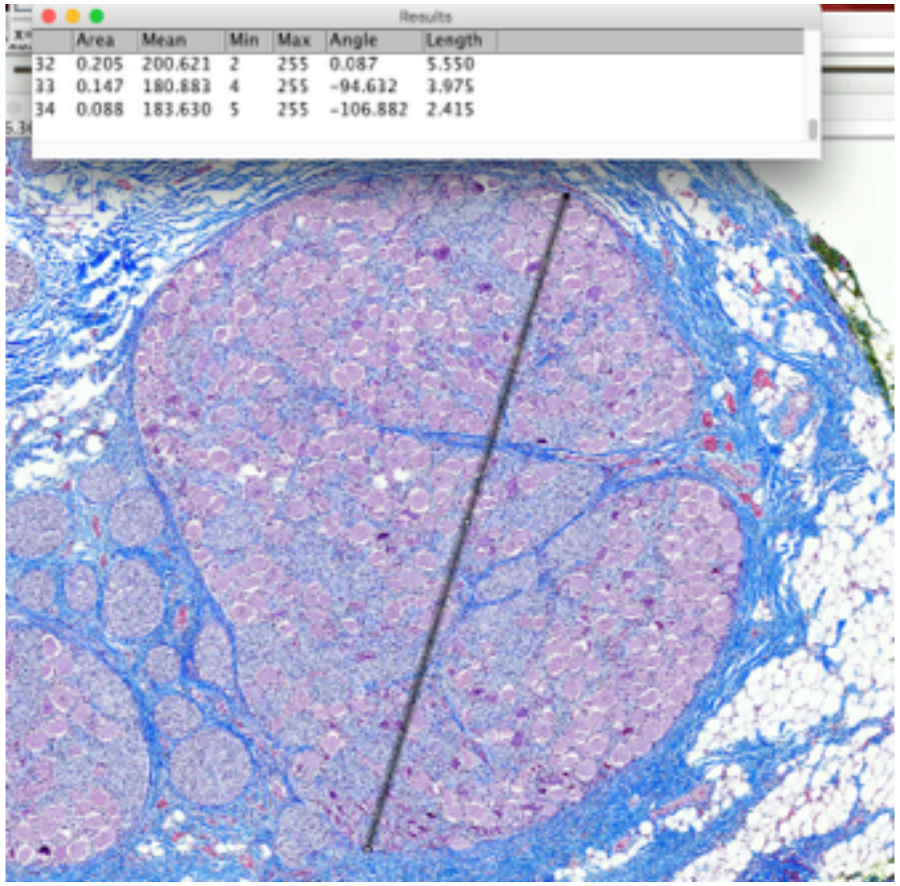

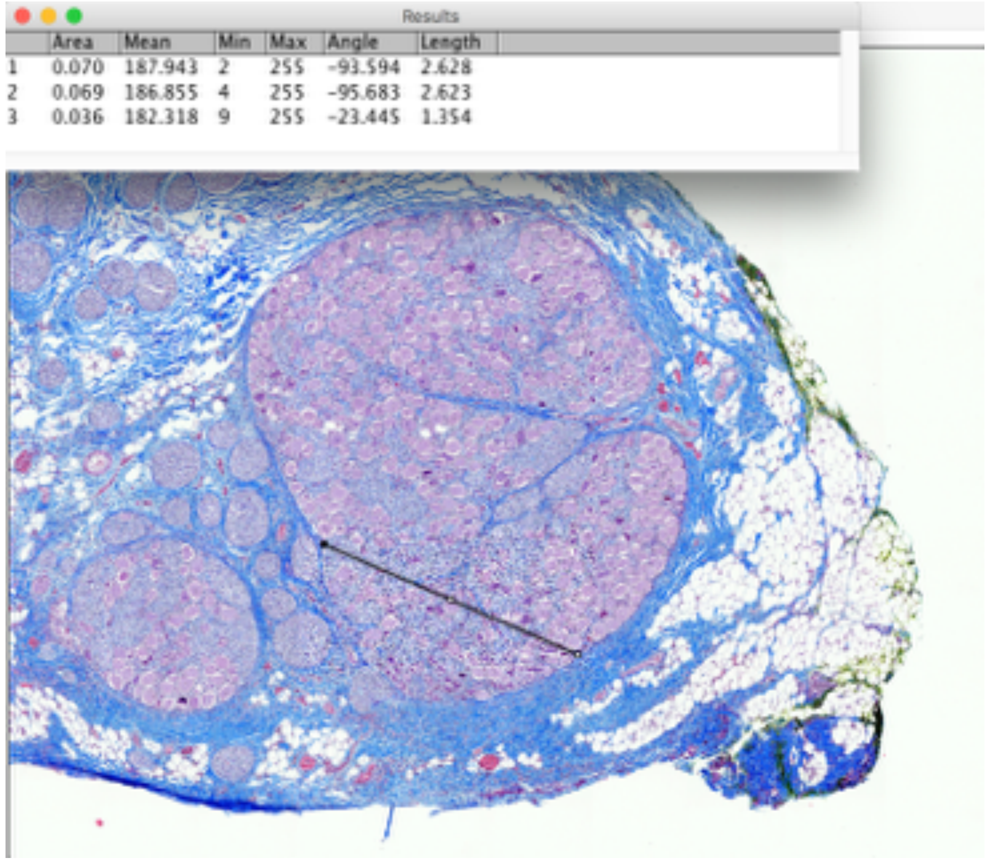

Largest Fascicle_Widest and narrowest diameter

P836_RV1_5

Mid-VN

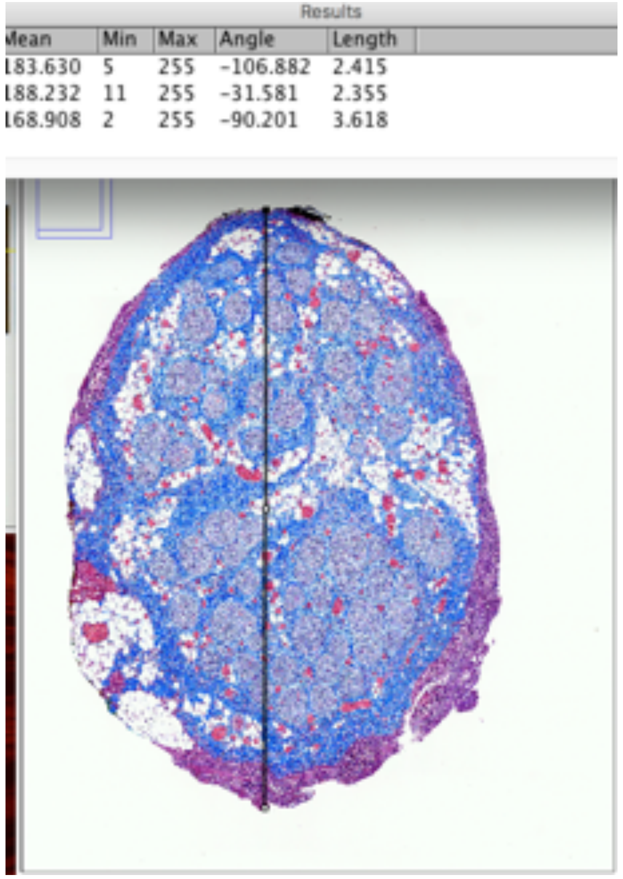

Widest and narrowest diameter

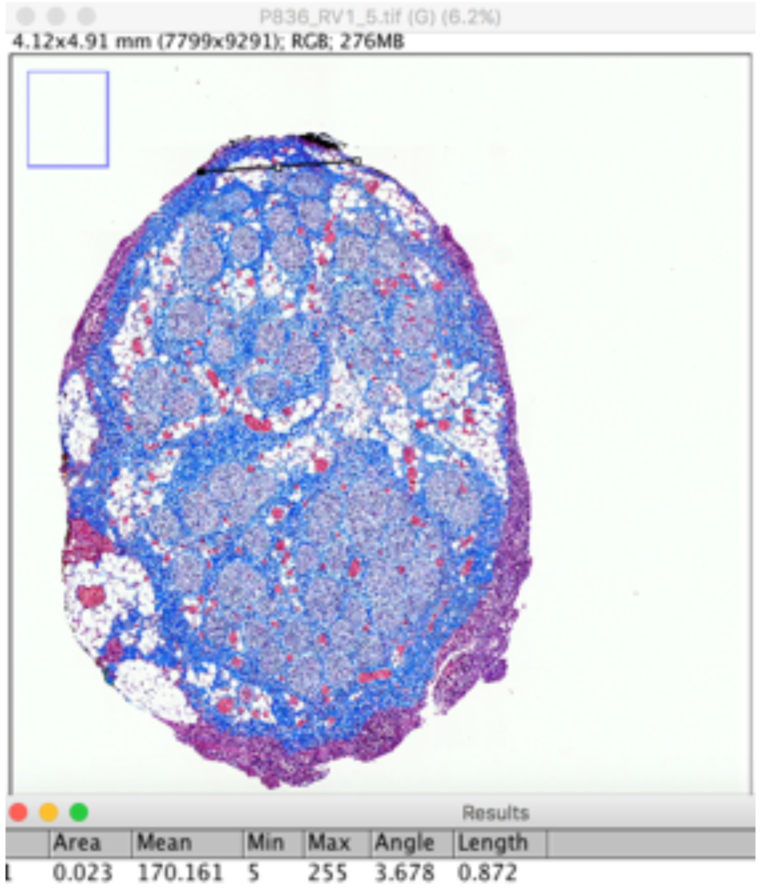

Fascicle depth

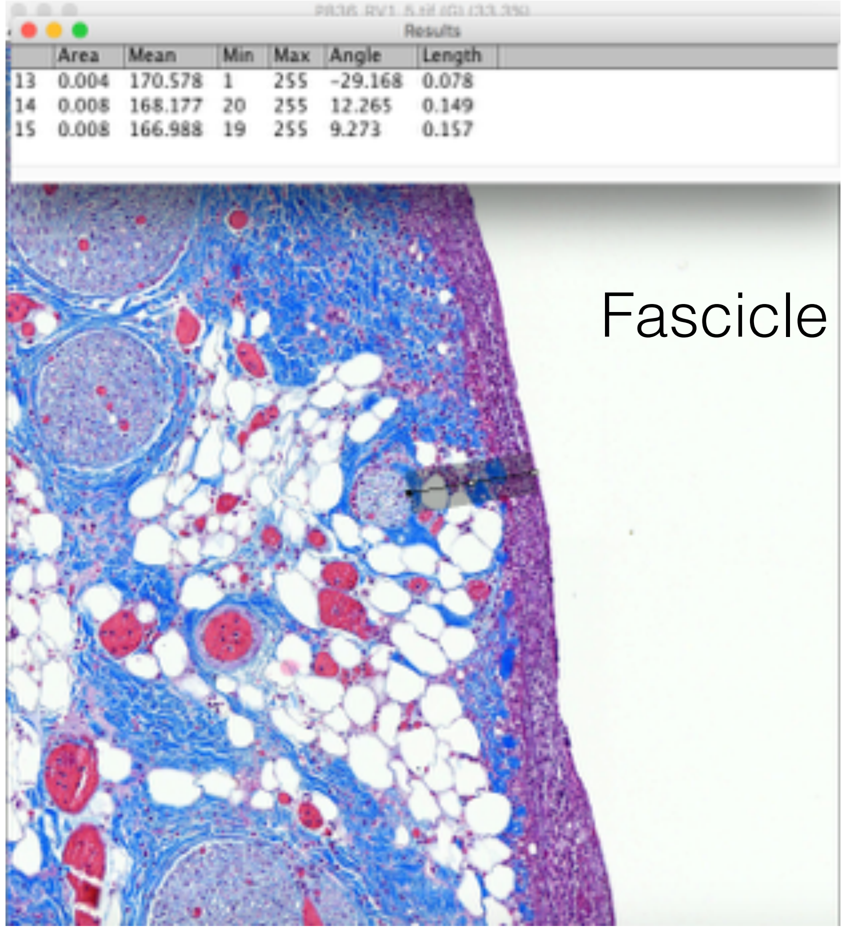

Largest Fascicle_Widest and narrowest diameter

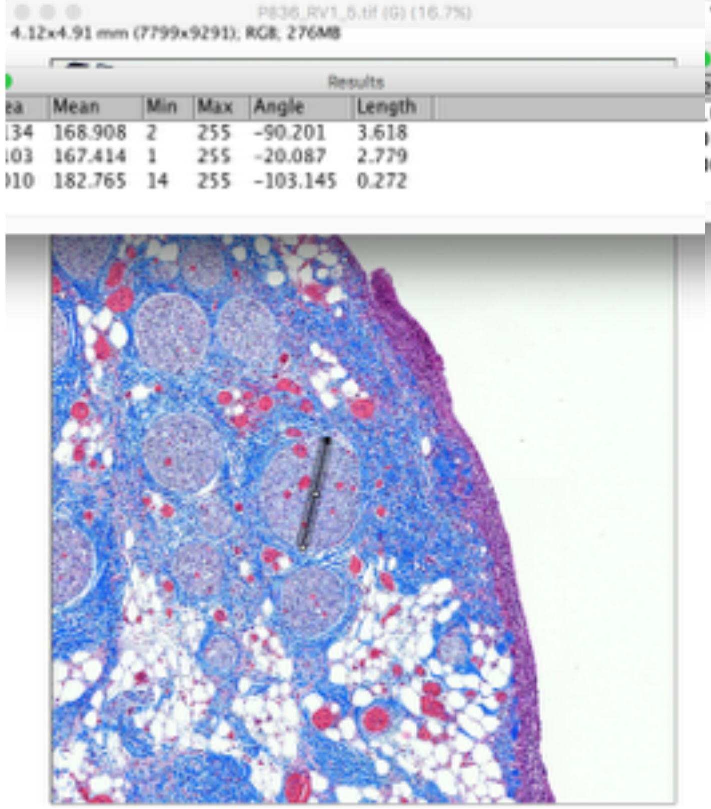

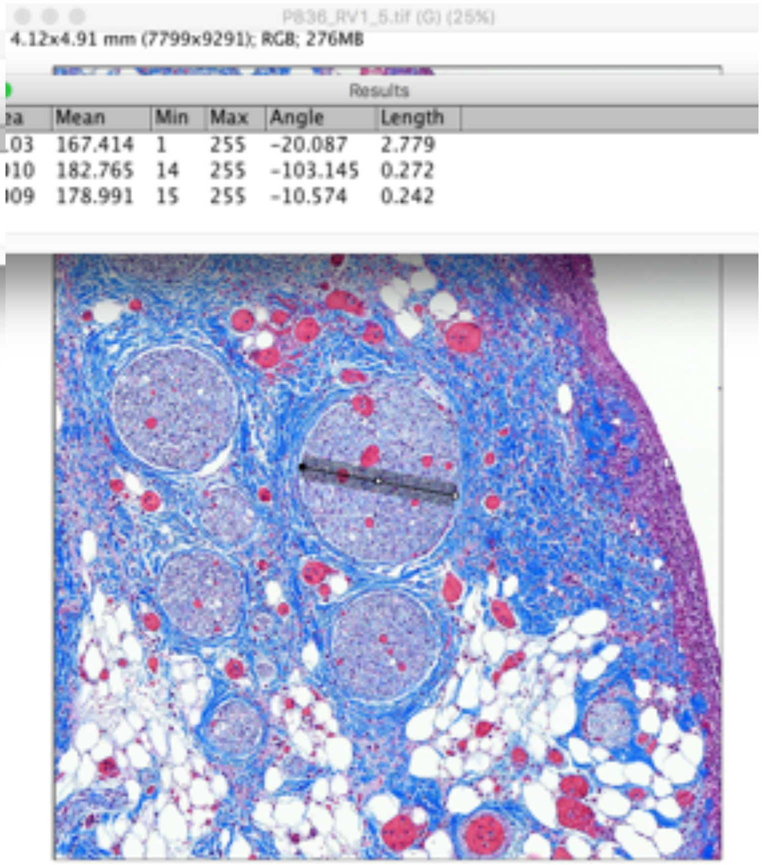

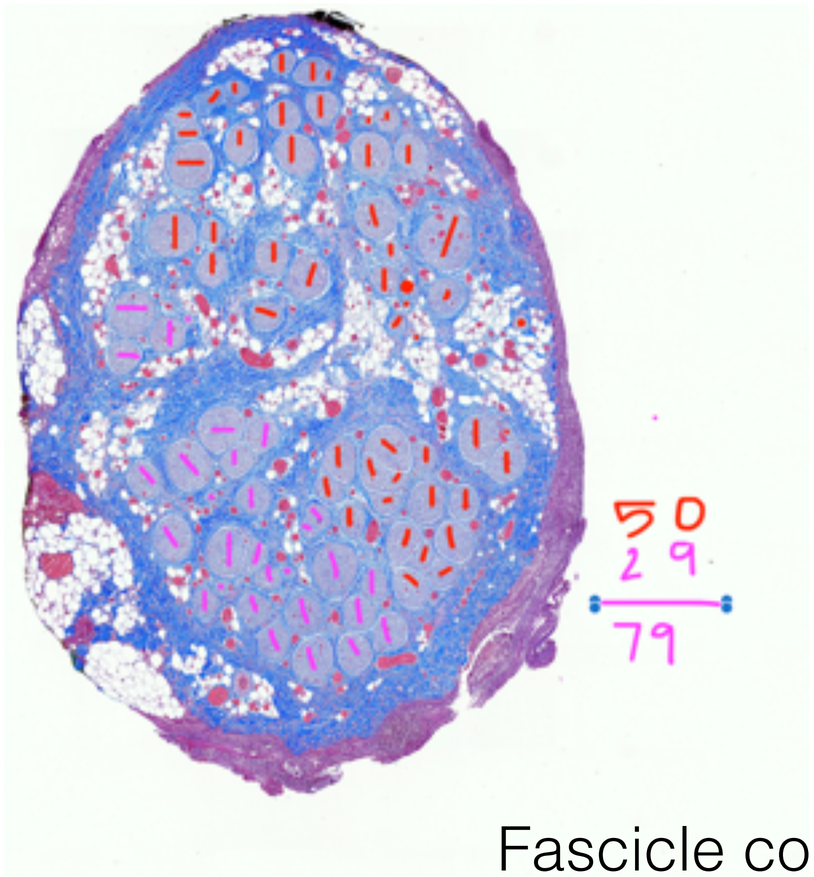

Fascicle count

Subject 8_P858

Pre-nodose

Widest and narrowest diameter

P858_nodose_35

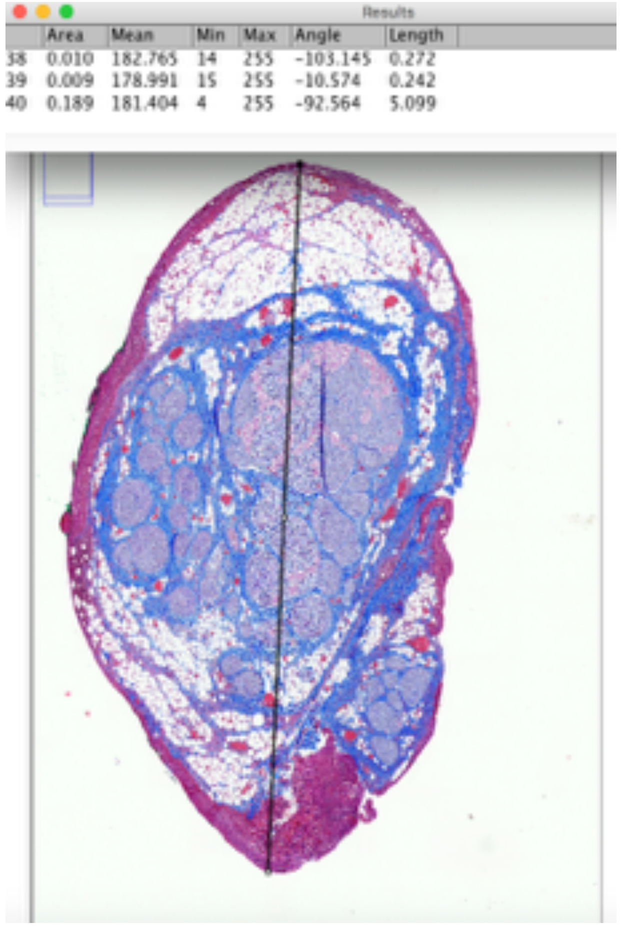

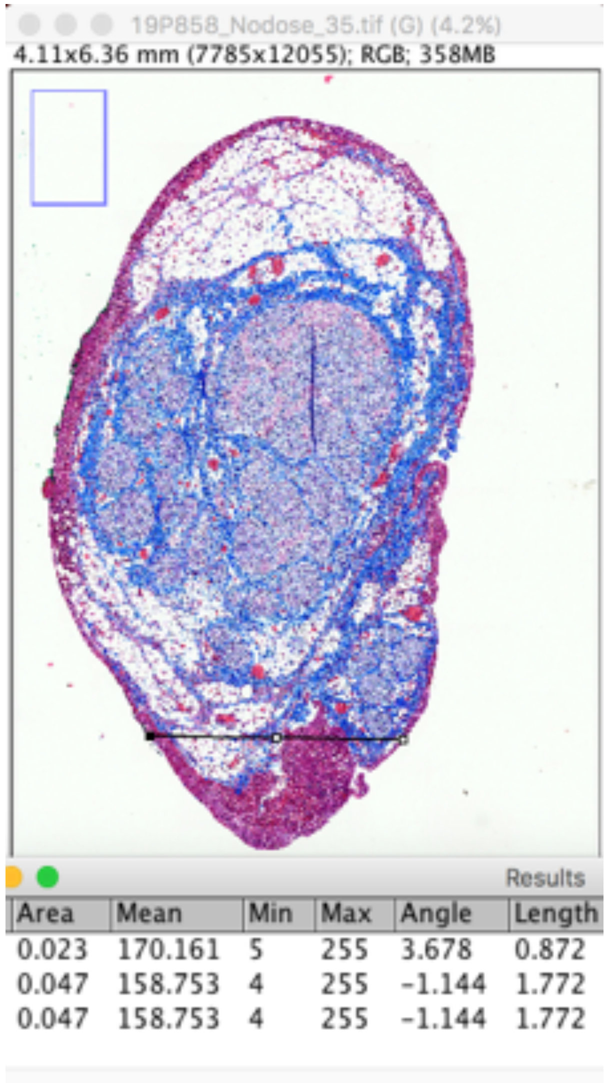

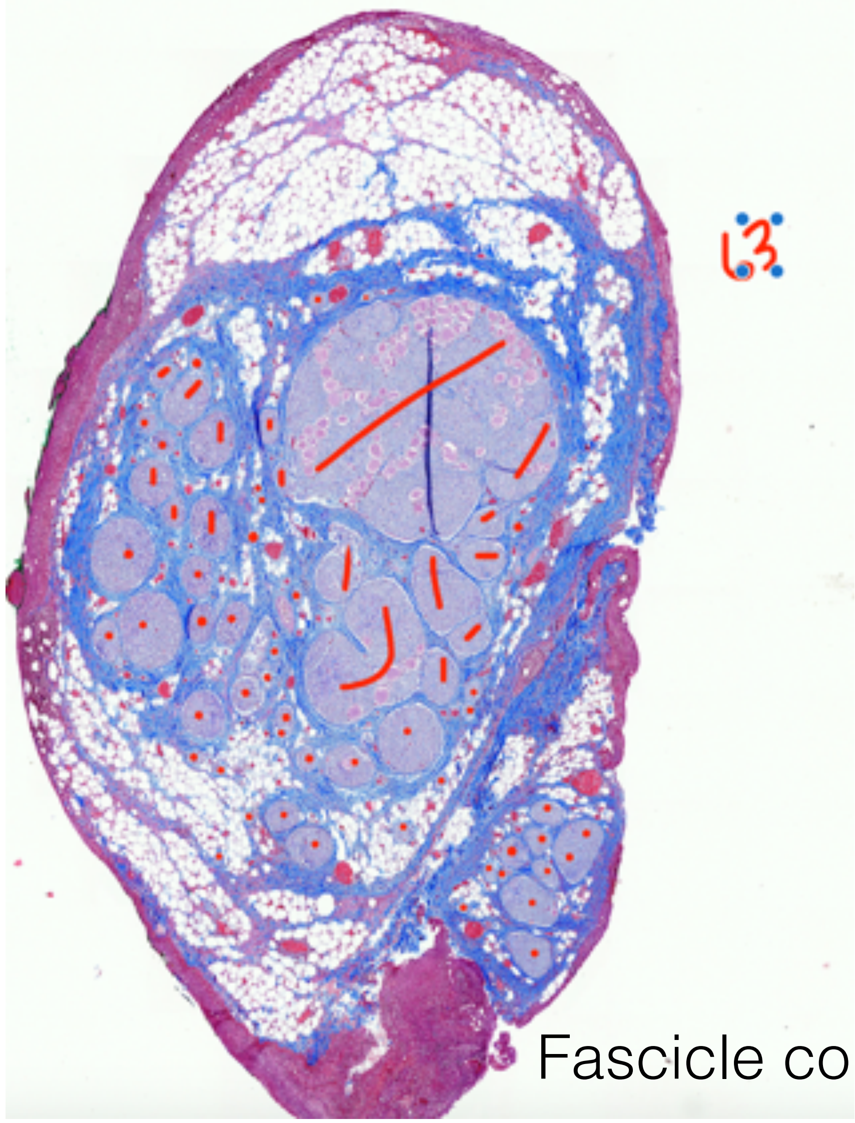

Fascicle count, 63

Largest fascicle_Widest and narrowest diameter

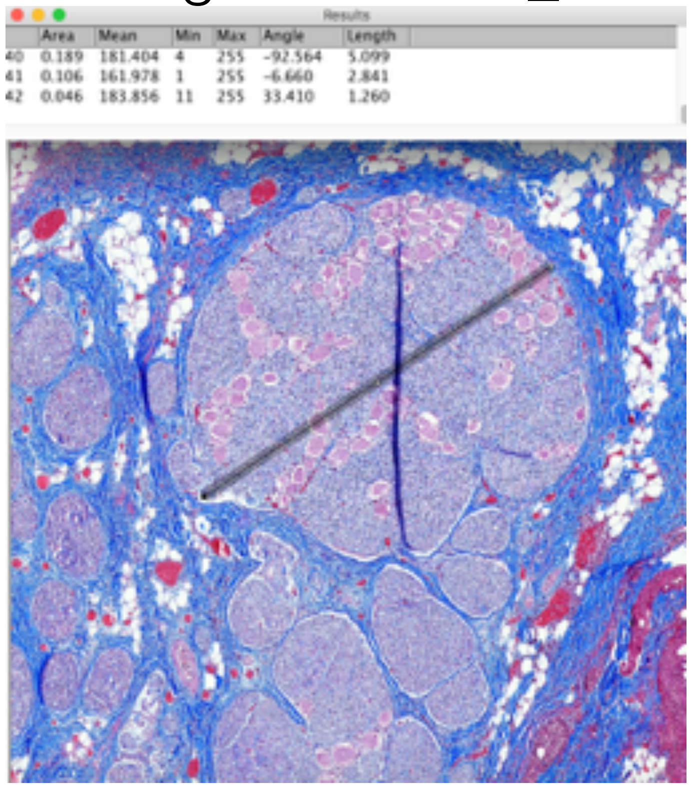

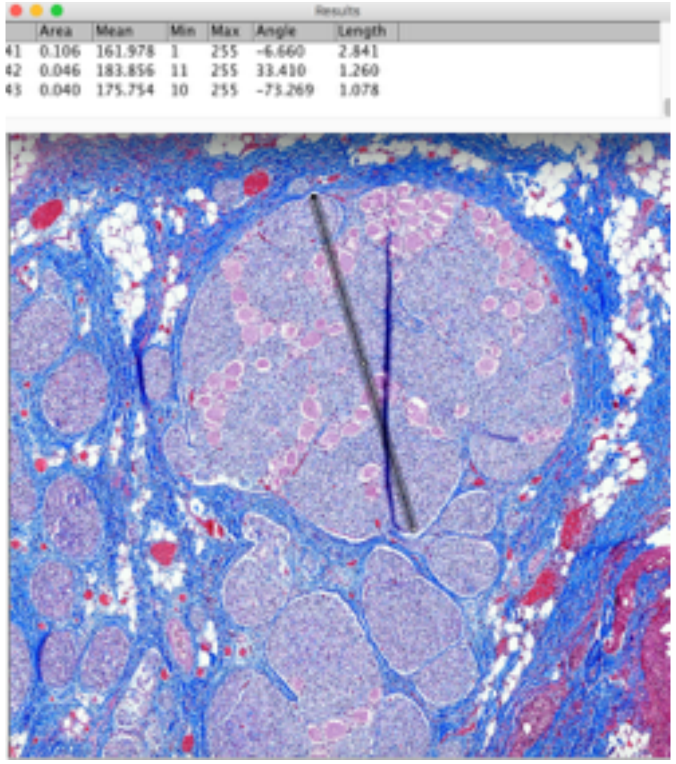

Nodose

Widest and narrowest diameter

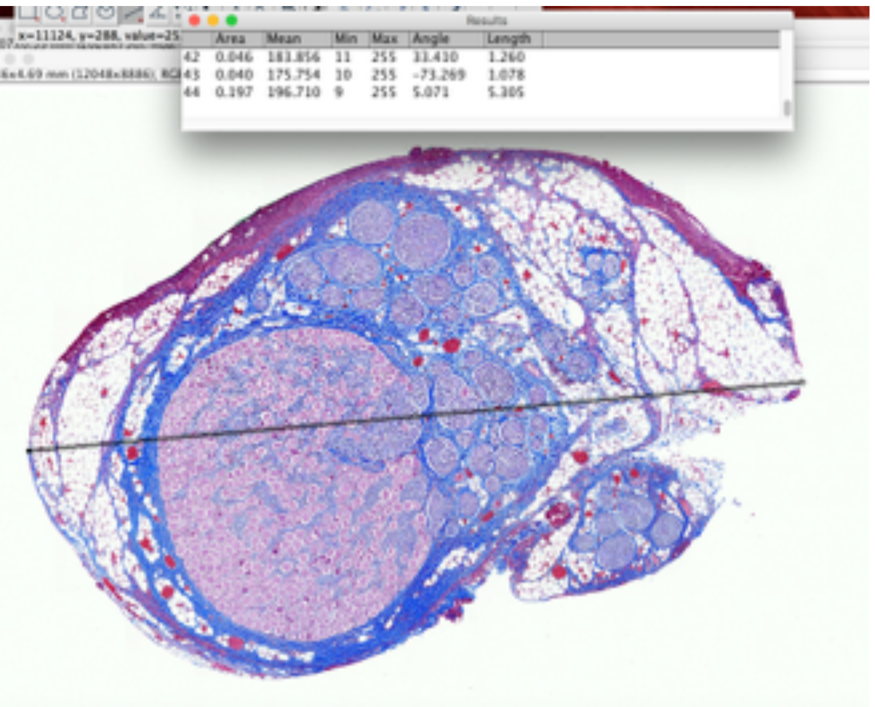

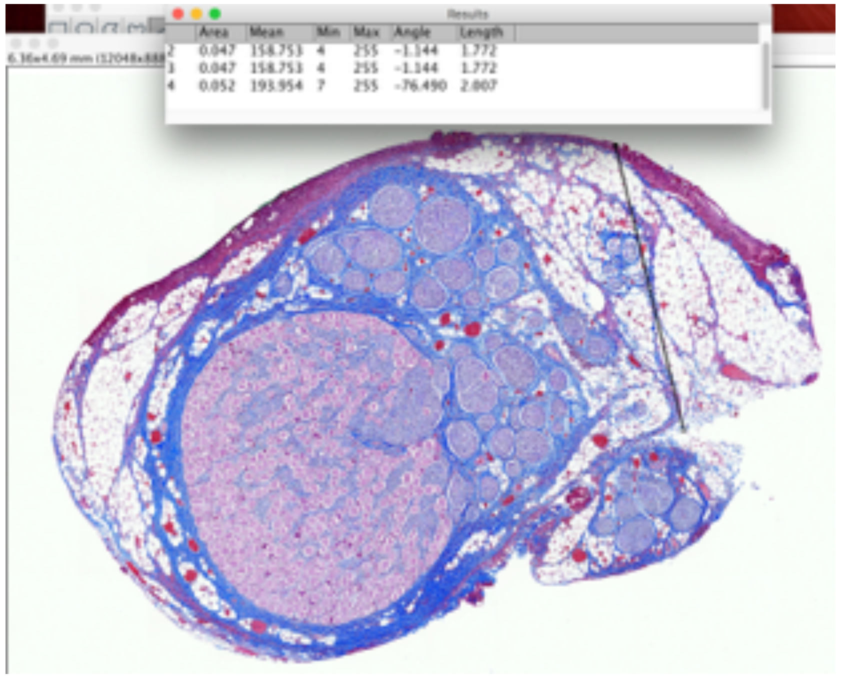

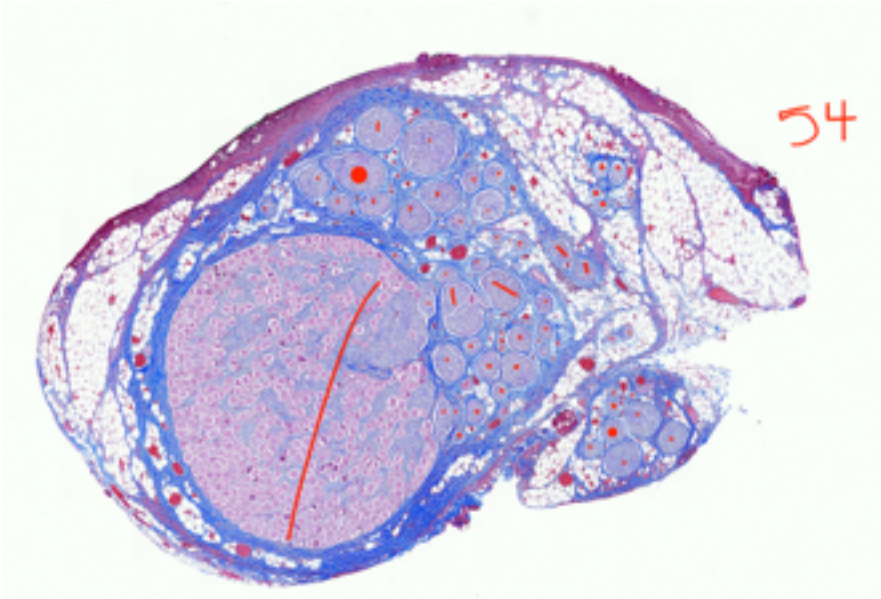

Fascicle count, 54

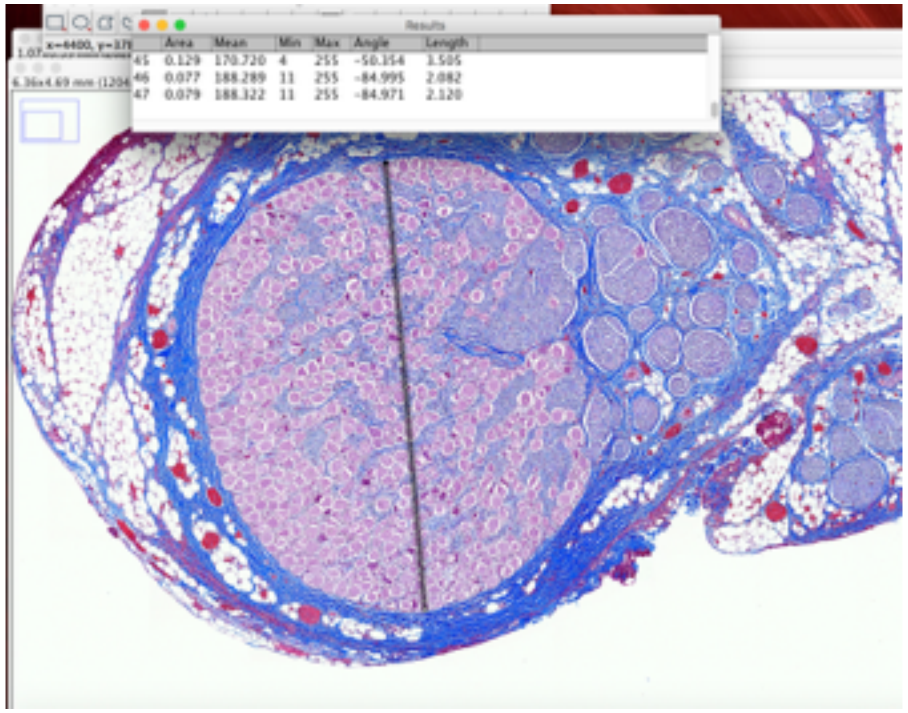

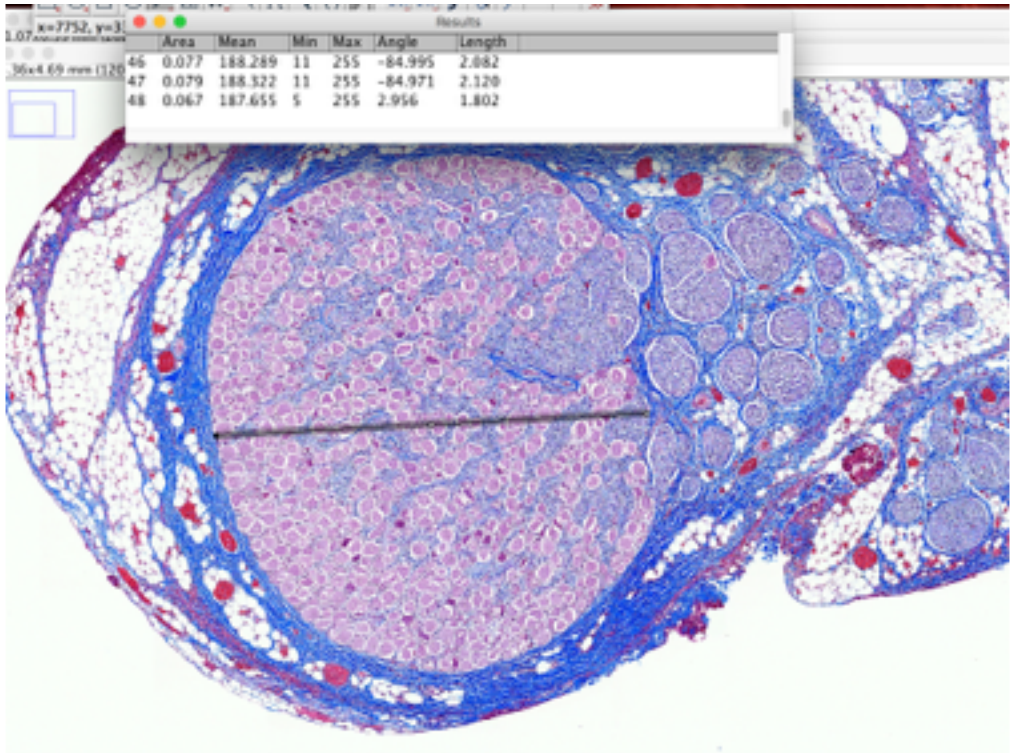

Largest Fascicle_Widest and narrowest diameter

Mid-VN

P858_LivaNova_43

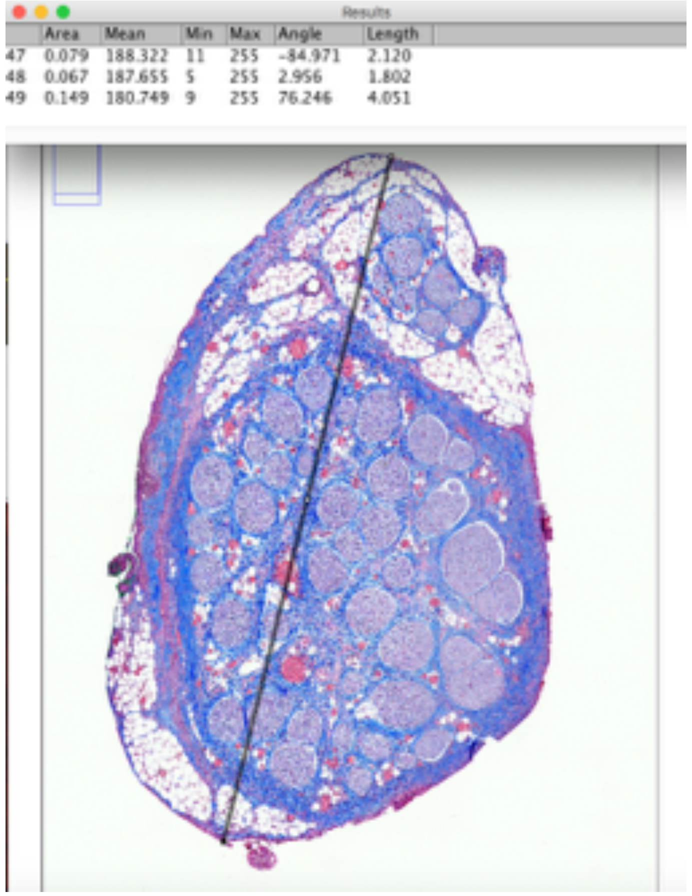

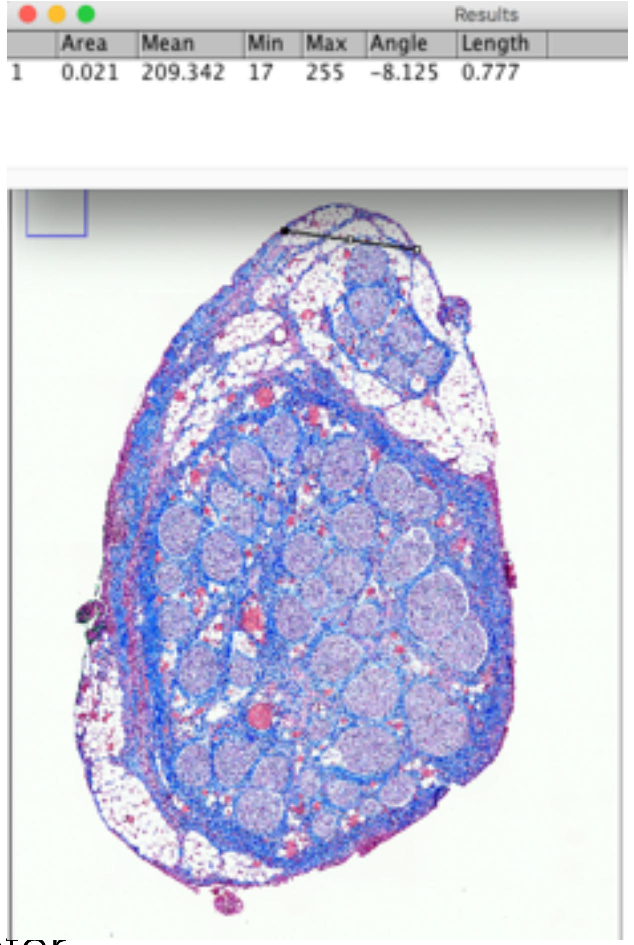

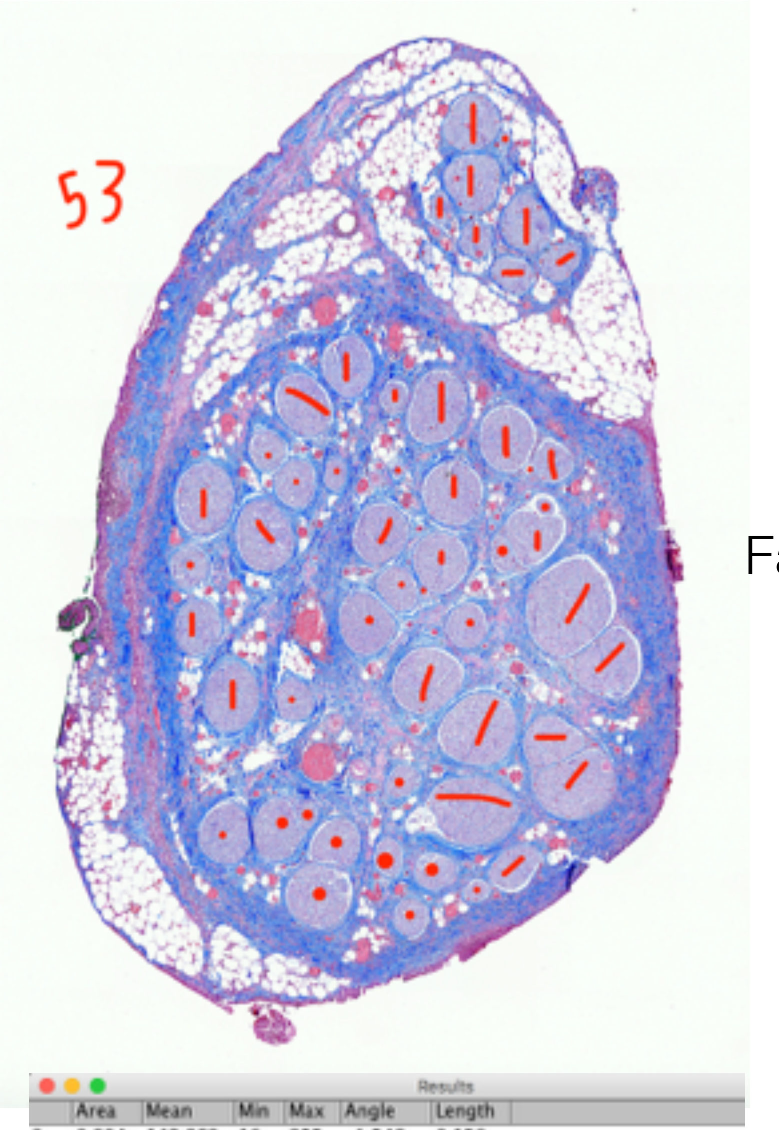

Fascicle count, 53

Widest and narrowest diameter

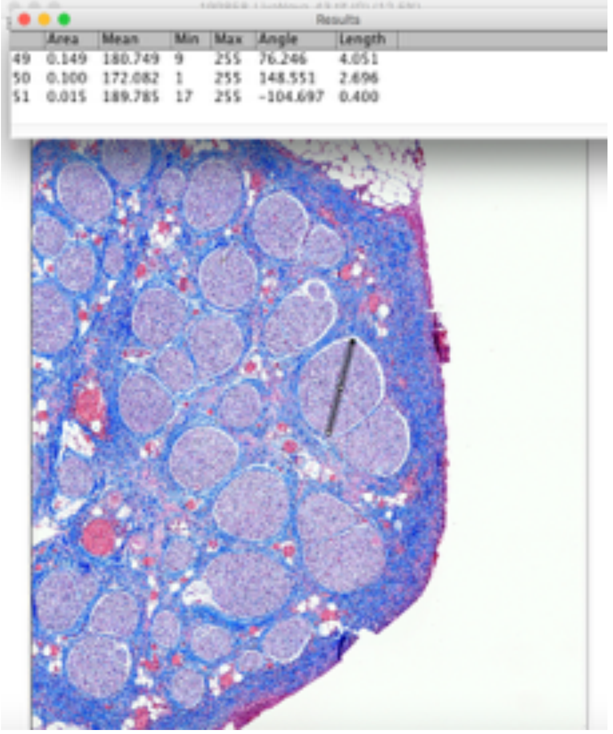

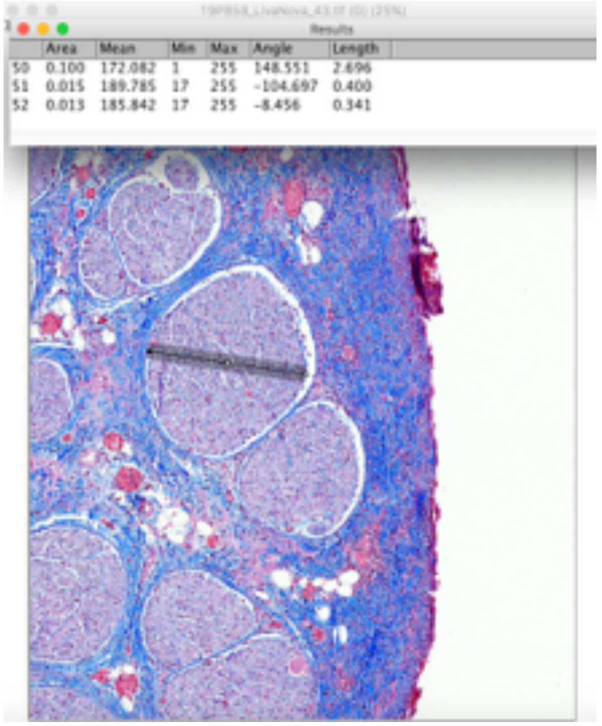

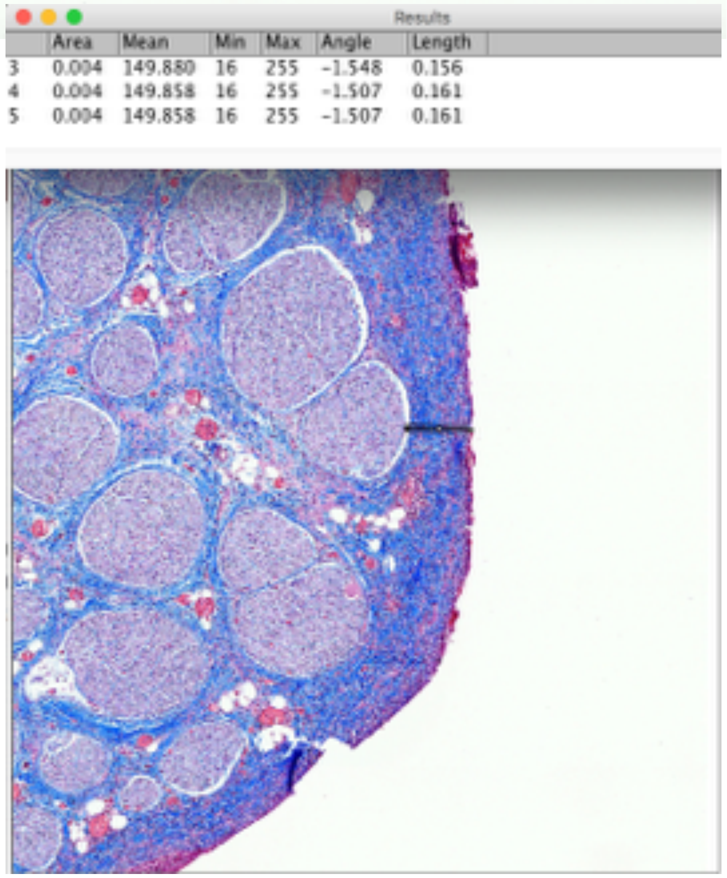

Fascicle depth

Largest Fascicle_Widest and narrowest diameter

Subject 10_P866

Pre-nodose

Widest and narrowest diameter

P866_Nodose_152

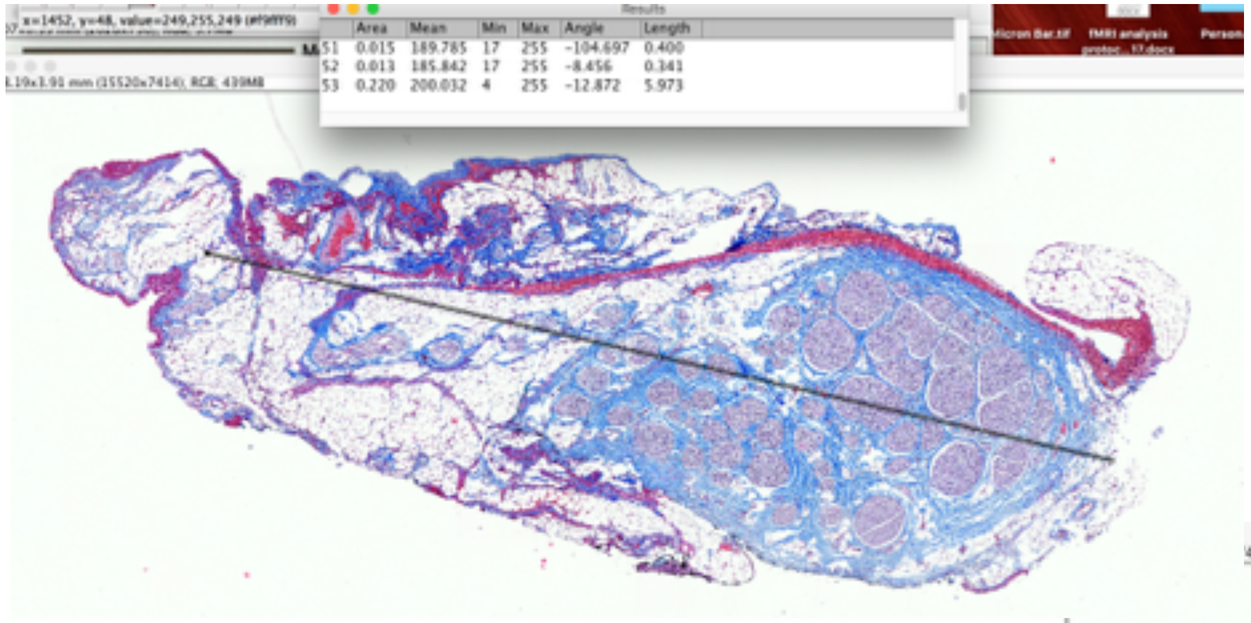

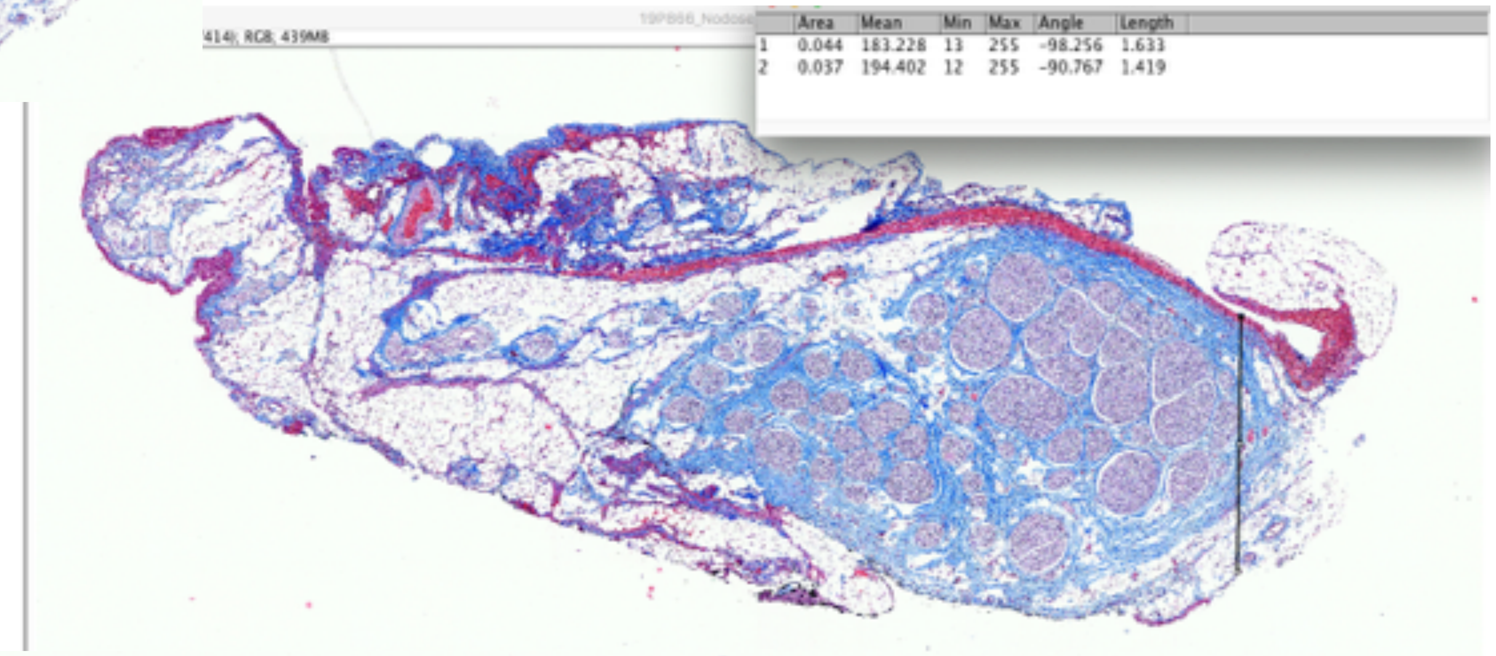

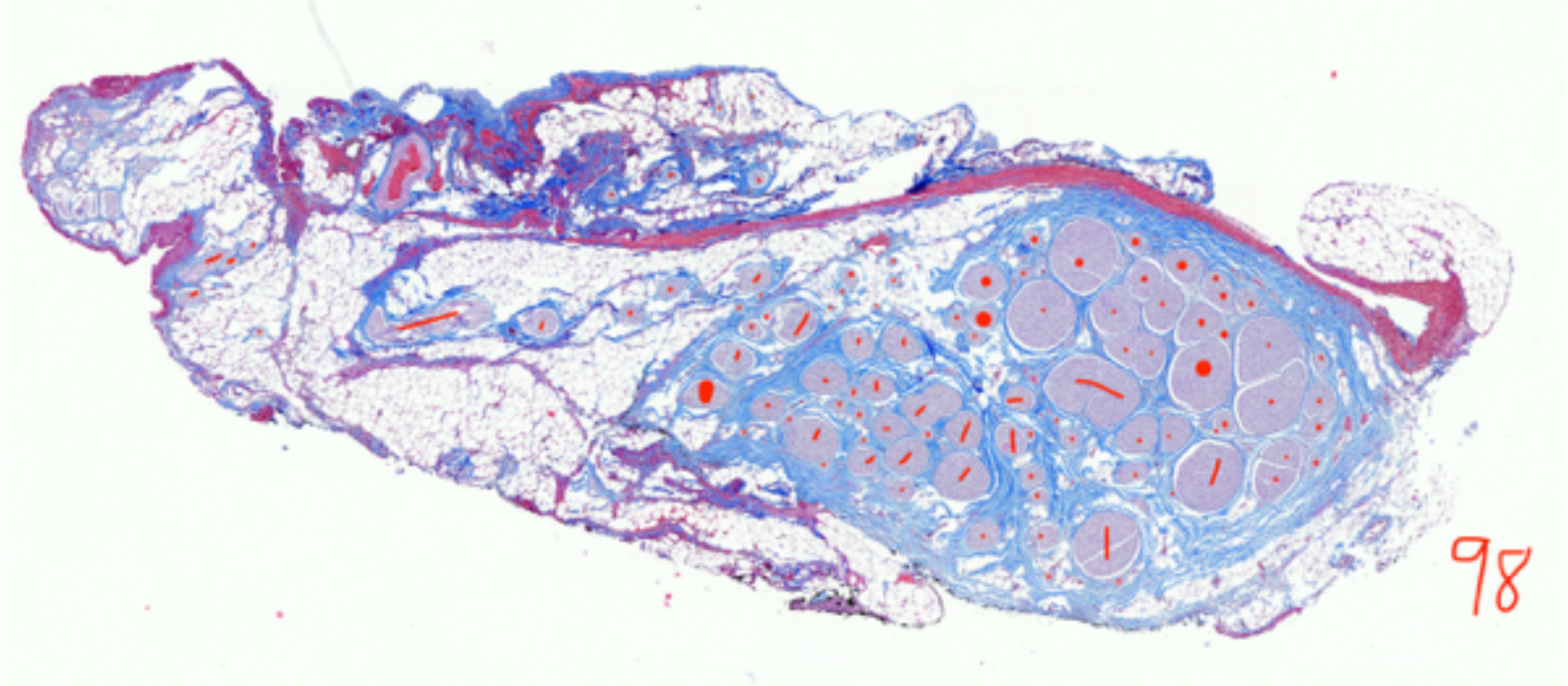

Fascicle count, 98

Pre-nodose

P866_Nodose_152

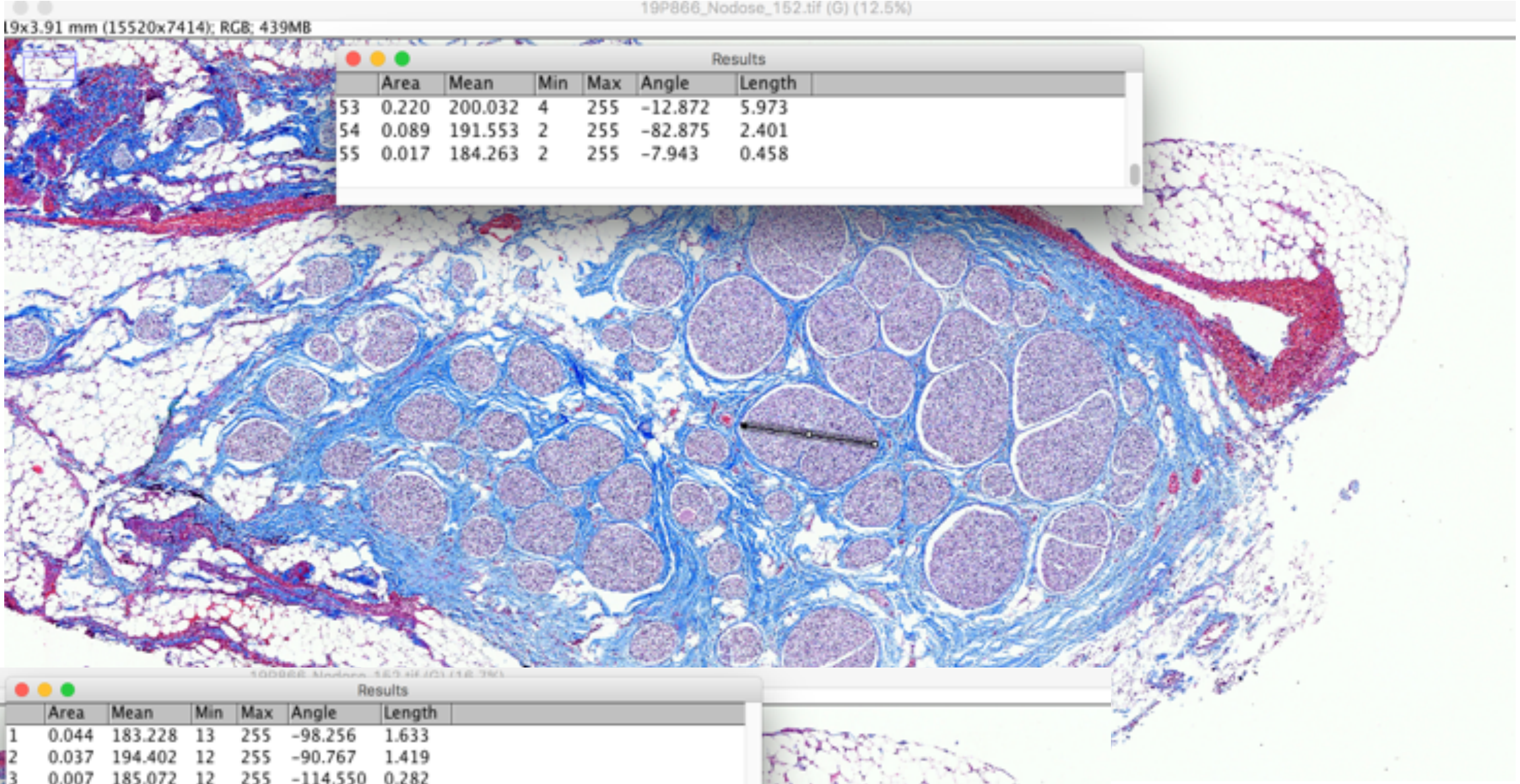

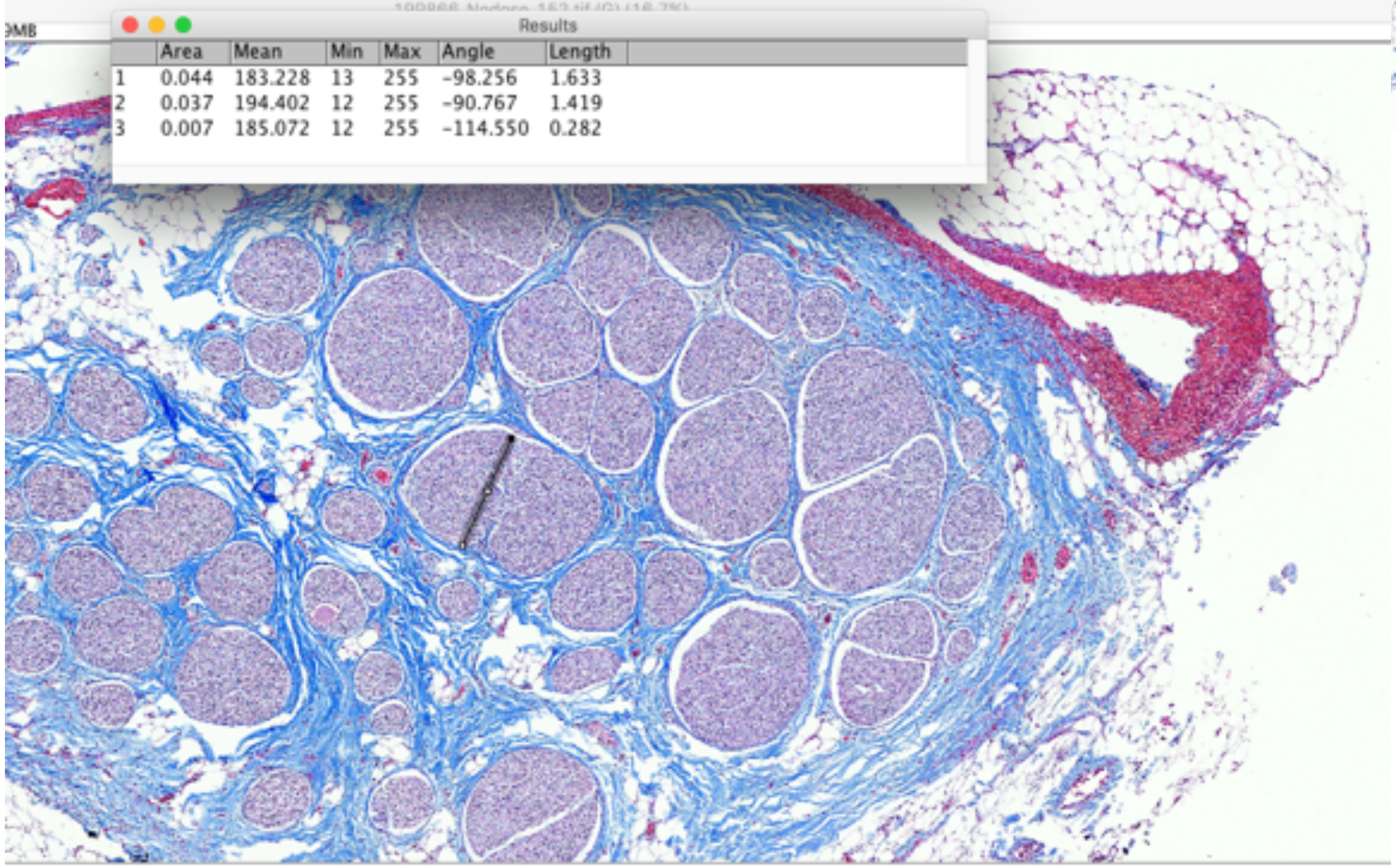

Largest Fascicle_Widest and narrowest diameter

Mid-VN

Widest and narrowest diameter

P866_VN1_A_154_blue

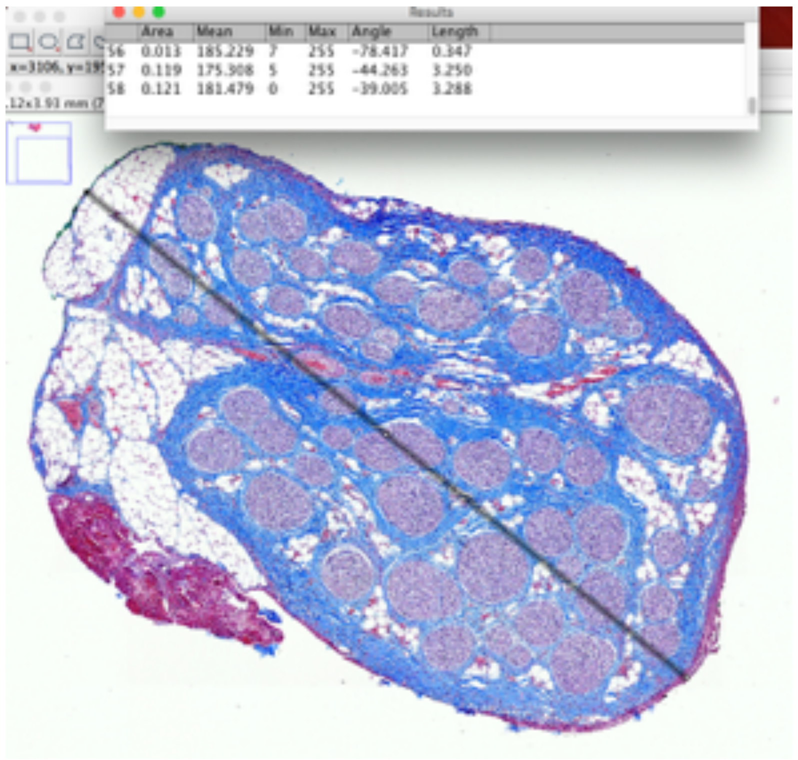

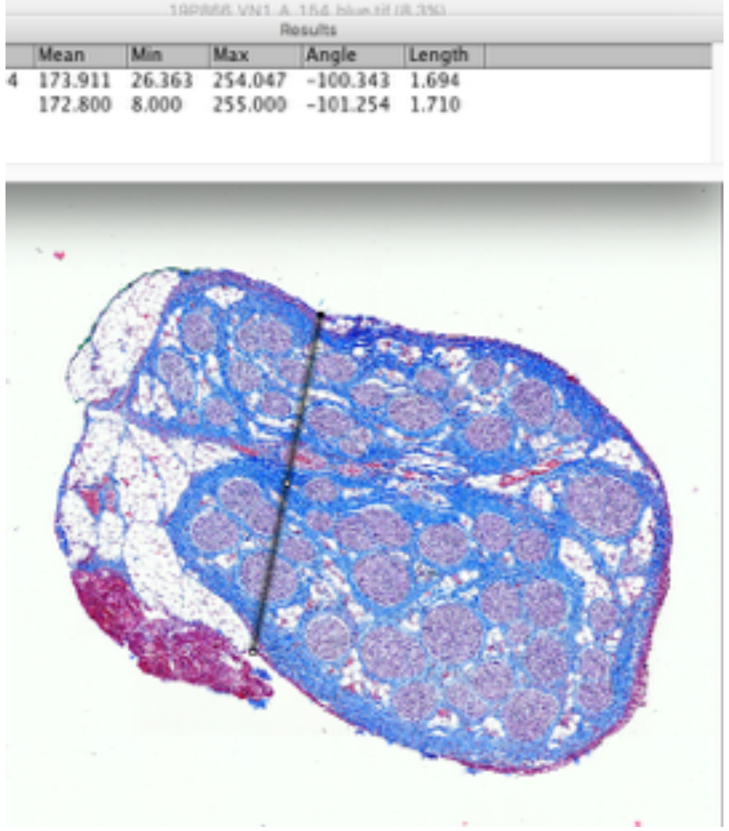

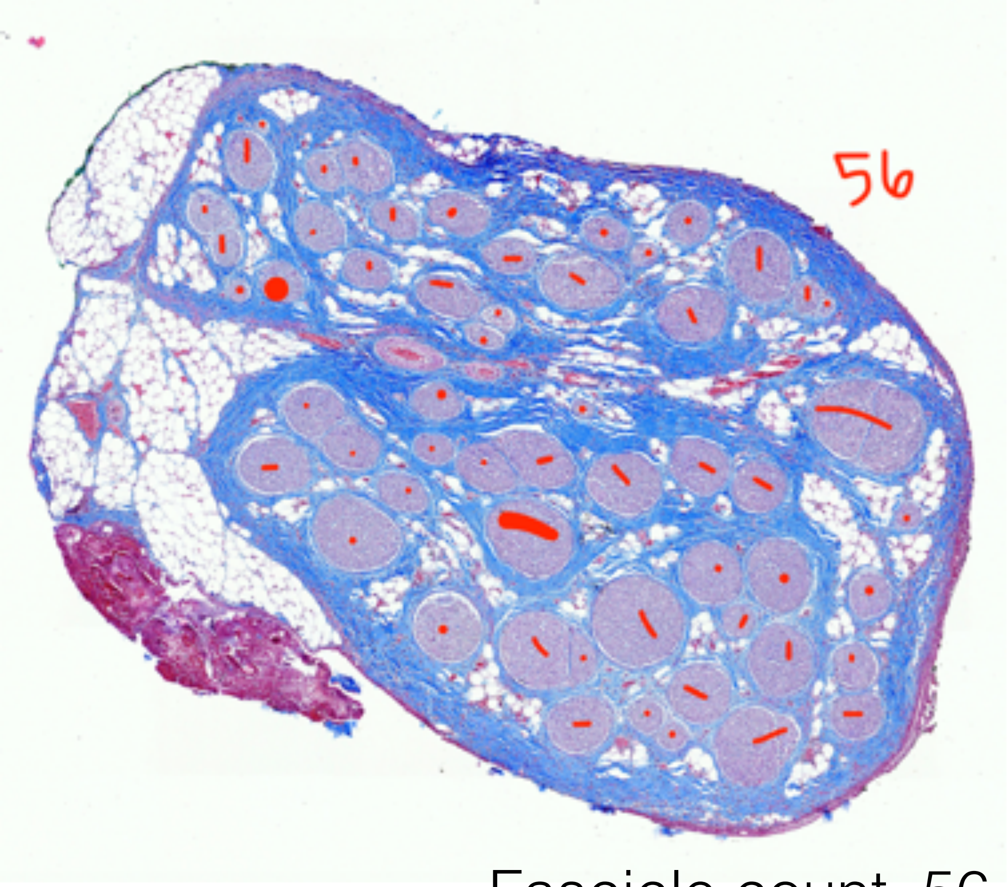

Fascicle count, 56

Largest fascicle_widest and narrowest diameter

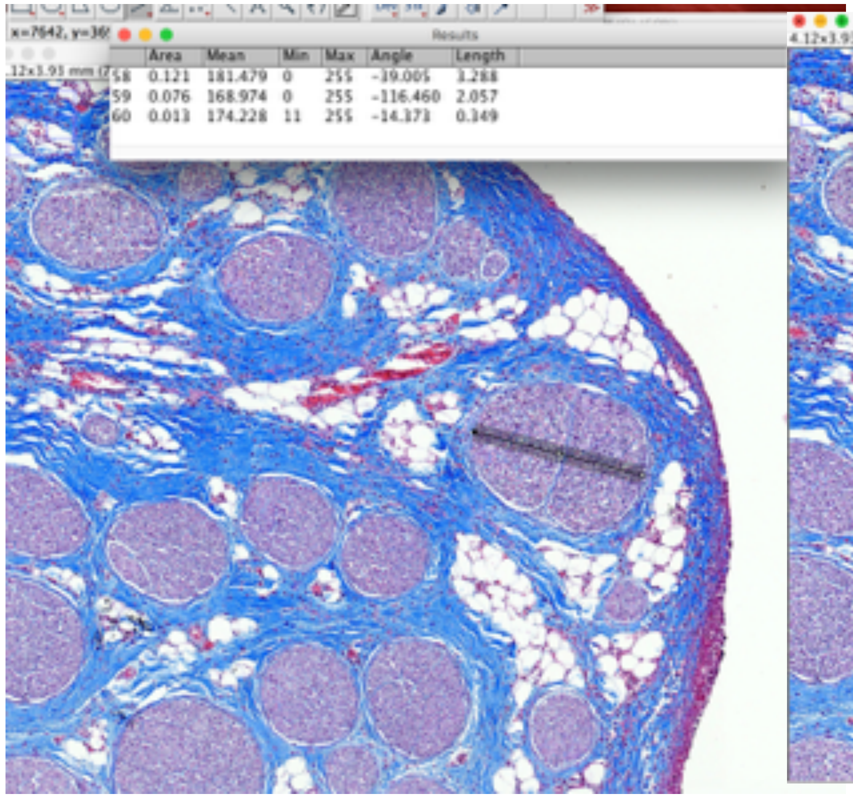

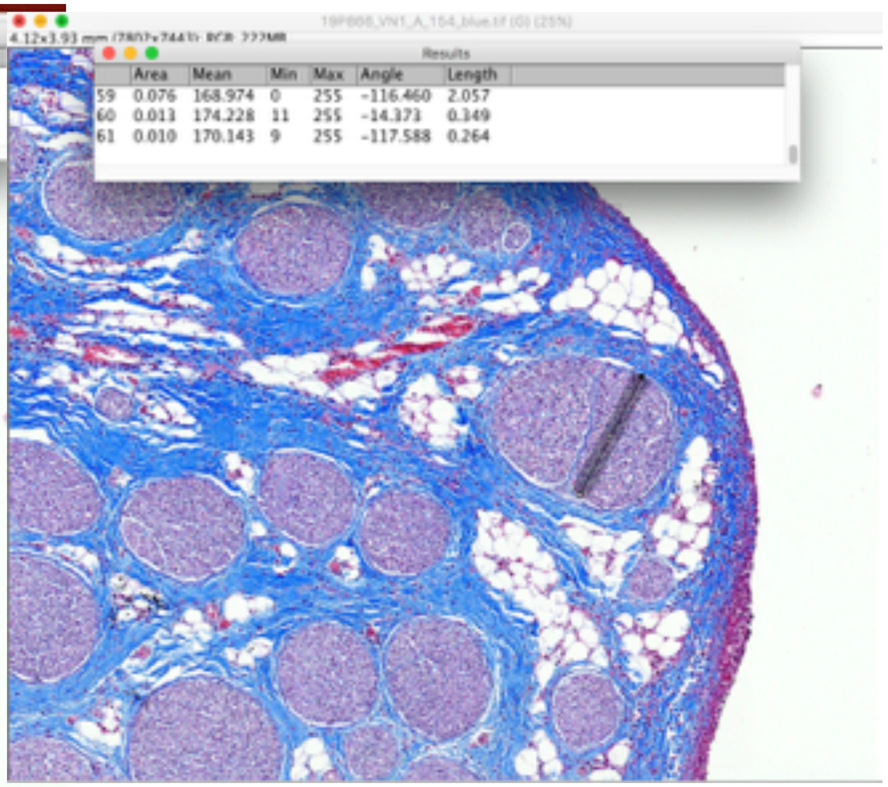

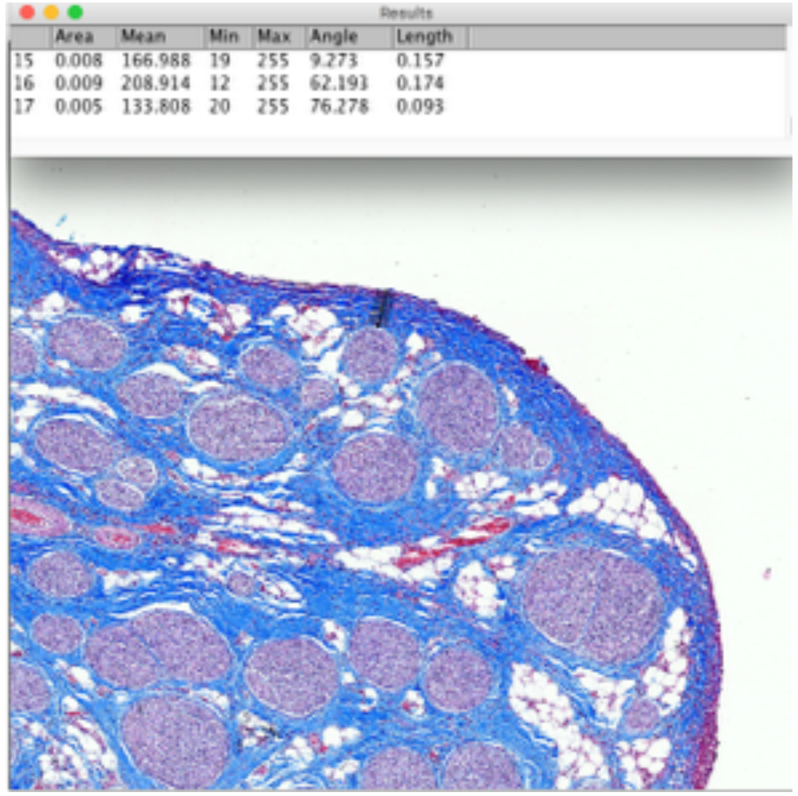

Fascicle depth
